# Global Transcriptomic Analysis of Placentas from Women with Gestational SARS-CoV-2 Infection during the Third Trimester of Pregnancy

**DOI:** 10.3390/ijms25031608

**Published:** 2024-01-28

**Authors:** Yiqun Tang, Nageswara Rao Boggavarapu, Annette Aronsson, Kristina Gemzell-Danielsson, Parameswaran Grace Lalitkumar

**Affiliations:** 1 WHO Collaborating Centre, Division of Neonatology, Obstetrics and Gynecology, Department of Women’s and Children’s Health, Karolinska University Hospital, Karolinska Institutet, SE 17176 Stockholm, Sweden; yiqun.tang@ki.se (Y.T.); nageswara.boggavarapu@ki.se (N.R.B.); annette.aronsson@ki.se (A.A.); kristina.gemzell@ki.se (K.G.-D.); 2 Department of Obstetrics and Gynecology, PEDEGO Research Unit, Medical Research Centre, Oulu University Hospital, University of Oulu, 90220 Oulu, Finland

**Keywords:** SARS-CoV-2, COVID-19, term pregnancy, vertical transmission, placental gene expression, pregnancy complication, molecular pathways

## Abstract

The COVID-19 pandemic has had a significant and enduring influence on global health, including maternal and fetal well-being. Evidence suggests that placental dysfunction is a potential consequence of SARS-CoV-2 infection during pregnancy, which may result in adverse outcomes such as preeclampsia and preterm birth. However, the molecular mechanisms underlying this association remain unclear, and it is uncertain whether a mature placenta can protect the fetus from SARS-CoV-2 infection. To address the above gap, we conducted a transcriptome-based study of the placenta in both maternal and fetal compartments. We collected placental samples from 16 women immediately after term delivery, seven of which had SARS-CoV-2 infection confirmed by PCR before parturition. Notably, we did not detect any viral load in either the maternal or fetal compartments of the placenta, regardless of symptomatic status. We separately extracted total RNA from placental tissues from maternal and fetal compartments, constructed cDNA libraries, and sequenced them to assess mRNA. Our analysis revealed 635 differentially expressed genes when a false discovery rate (FDR ≤ 0.05) was applied in the maternal placental tissue, with 518 upregulated and 117 downregulated genes in the SARS-CoV-2-positive women (*n* = 6) compared with the healthy SARS-CoV-2-negative women (*n* = 8). In contrast, the fetal compartment did not exhibit any significant changes in gene expression with SARS-CoV-2 infection. We observed a significant downregulation of nine genes belonging to the pregnancy-specific glycoprotein related to the immunoglobulin superfamily in the maternal compartment with active SARS-CoV-2 infection (fold change range from −13.70 to −5.28; FDR ≤ 0.01). Additionally, comparing symptomatic women with healthy women, we identified 1788 DEGs. Furthermore, a signaling pathway enrichment analysis revealed that pathways related to oxidative phosphorylation, insulin secretion, cortisol synthesis, estrogen signaling, oxytocin signaling, antigen processing, and presentation were altered significantly in symptomatic women. Overall, our study sheds light on the molecular mechanisms underlying the reported clinical risks of preeclampsia and preterm delivery in women with SARS-CoV-2 infection. Nonetheless, studies with larger sample sizes are warranted to further deepen our understanding of the molecular mechanisms of the placenta’s anti-viral effects in maternal SARS-CoV-2 infection.

## 1. Introduction

The coronavirus disease 2019 (COVID-19) pandemic caused by the severe acute respiratory syndrome coronavirus 2 virus (SARS-CoV-2) was a global health concern until very recently. Despite extensive research, the precise ramifications of this viral infection on pregnancy and its subsequent effects on offspring remain elusive [1]. Generally, pregnant individuals are more susceptible to viral infections compared to the general population [2]. Reports indicated that contracting SARS-CoV-2 during pregnancy can have detrimental implications for maternal well-being by increasing the risks of preeclampsia, preterm delivery, and maternal mortality and impacting neonatal outcomes, with increased incidences of preterm birth, neonatal intensive care unit admission, and neonatal mortality [3]. However, the intricate molecular mechanisms that underline such adverse effects during pregnancy with SARS-CoV-2 infection are not clearly understood.

According to current reports, the SARS-CoV-2 virus relies on two key proteins, angiotensin-converting enzyme II (ACE2) and transmembrane serine protease 2 (TMPRSS2), for cell entry and membrane fusion, respectively [4,5]. It is reported that both ACE2 and TMPRSS2 are enriched in many tissues in addition to the respiratory system, resulting in pathological alterations within local tissues upon SARS-CoV-2 infection. The term placenta is one of the organs in which the above-mentioned entry factors for SARS-CoV-2, along with Furin, are highly expressed. Notably, the expression of ACE2 mRNA in the placenta is similar to that in the lung, the most common site for SARS-CoV-2 infection in humans [6].

The placenta, a remarkable and intricate tissue, plays a vital role in facilitating the communication channel between the mother and developing fetus by transferring nutrients, hormones, and antibodies protecting the growing fetus from immunological threats [7]. Earlier reports suggested a possible vertical transmission of SARS-CoV-2 from the mother to the fetus [8,9]. However, recent studies suggest that the placenta might act as a barrier for transplacental infection since a restriction of SARS-CoV-2 replication was observed in human placental tissue [10]. Nonetheless, controversies persist regarding the possibility of antenatal vertical transmission of SARS-CoV-2.

Hence, the primary objectives of this study were twofold. Firstly, we aimed to investigate the potential vertical transmission of the SARS-CoV-2 virus by examining its presence in both the maternal and placental compartments of the term placenta. Secondly, we aimed to explore differentially expressed genes within placental compartments that are altered in COVID-19 infection during pregnancy, with a particular focus on understanding how these genes and related pathways may contribute to pregnancy-related complications. By investigating these molecular pathways, we aimed to shed light on the underlying mechanisms that could lead to adverse outcomes in pregnant women infected with SARS-CoV-2.

## 2. Results

### 2.1. Clinical Characteristics of SARS-CoV-2-Positive and Healthy Women

In this prospective study, a total of 16 term placental samples collected from women between June 2020 and February 2022 were used. Of these, seven women tested positive for SARS-CoV-2 by qPCR when admitted to hospital during their term pregnancy for delivery; details are given in Table 1. The general inclusion criteria encompassed women admitted for delivery in their third trimester of pregnancy with a normal, healthy, and viable singleton pregnancy. Exclusion criteria included a history of preeclampsia, known reproductive tract infections, immune disorders, and regular medications for chronic illnesses. Briefly, the average maternal age of the women who tested positive for SARS-CoV-2 was 34.14 years (range 31–37 years; median 32 years). The gestational age at delivery averaged 39.1 weeks (range 34.7–41.3 weeks; median 39.6 weeks). Cases 4, 13, 15, and 17 were for cesarean sections due to different indications including the failed induction of labor, social factors, and unexpected vaginal bleeding. All the other cases had a vaginal delivery. Except for case 15, all women had an active infection and tested positive for SARS-CoV-2 within 5 days before their delivery. Case 15 was diagnosed with COVID-19 three months before delivery and had completely recovered at the time of delivery and was thus excluded from the analysis. Cases 13, 14, 16, and 17 had mild symptoms of COVID-19, while the rest were asymptomatic when they tested positive for SARS-CoV-2. The severity of COVID-19 was determined according to the published criteria released by the Society for Maternal-Fetal Medicine and the National Institutes of Health [11]. The general parameters of the healthy women and newborns were comparable. The women in the control group were healthy, had negative SARS-CoV-2 PCR test results, and were free from any symptoms of COVID-19. The ages of the healthy women ranged from 27 to 43 years, and they had no other co-morbidities. The modes of delivery for both groups included both vaginal delivery and cesarian section. All newborns from both COVID-19-positive and healthy mothers were singletons and had normal Apgar scores after birth. None of the newborns were admitted to the neonatal intensive care unit (NICU).

### 2.2. Results from qPCR for SARS-CoV-2 Detection in Term Placenta

The results from a qPCR analysis showed no detectable levels of the SARS-CoV-2 virus in both the maternal and fetal compartments of the samples. The positive control supplied in the kit by the manufacturer and the clinical samples, both positive and negative for COVID-19, showed appropriate results as expected.

### 2.3. Differential Gene Expression Analysis of SARS-CoV-2-Positive and Healthy Women

**Figure 1:**
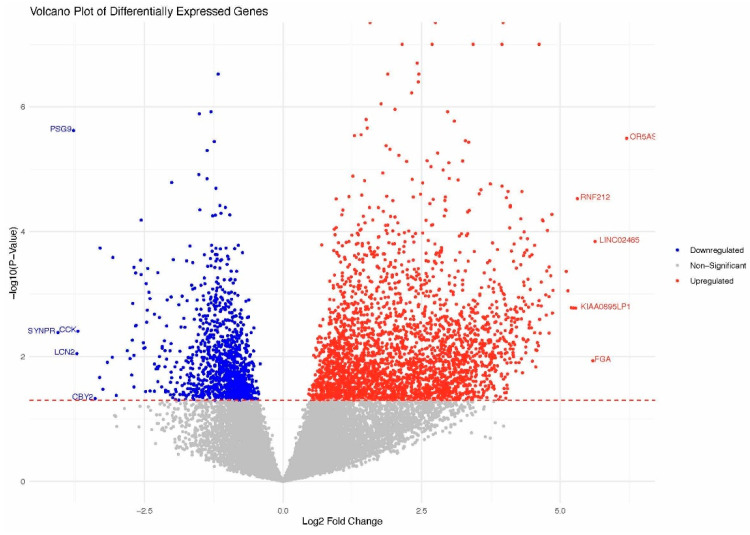
Volcano plot showing differentially expressed genes in the maternal compartment of the placenta with active COVID-19 infection. Up- and downregulated genes are highlighted in red and blue colors, respectively.

**Figure 2:**
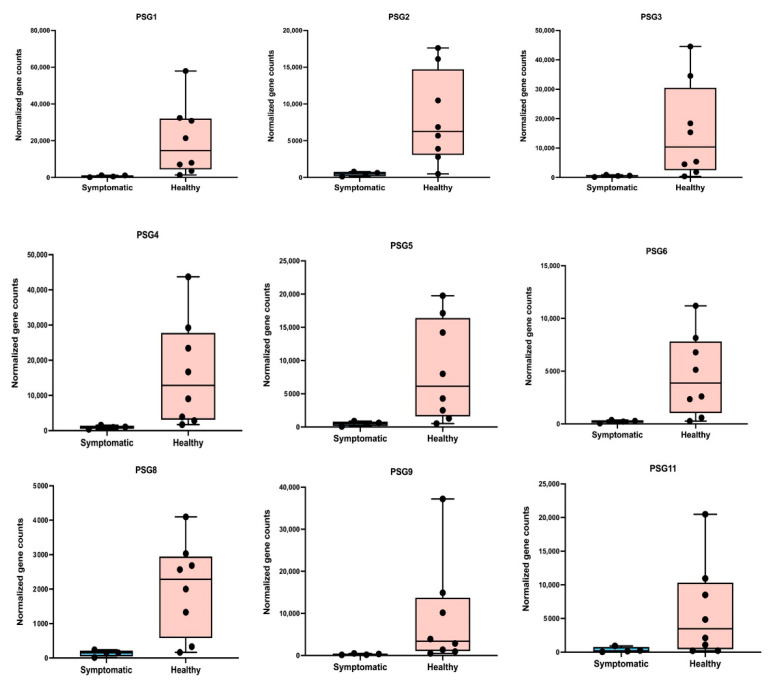
Tukey plots for PSG genes in maternal placental compartments from women with COVID-19 infection. Gene expression at an FDR level of 0.01 shows significant downregulation of the PSGs 2, 3, 4, 5, 6, and 9 genes in the placental maternal compartments of COVID-19-positive women. In addition to the above genes, PSGs 1, 8, and 11 were downregulated in the placentas of women with COVID-19 symptoms.

We obtained an mRNA and small RNA sequencing data set from 32 placental samples (16 maternal compartment samples + 16 fetal compartment samples) after performing quality control and batch effect correction (COVID-19 positive maternal (M) compartment, *n* = 7; COVID-19 negative maternal compartment, *n* = 9; COVID-19 positive fetal (F) compartment, *n* = 7; COVID-19 negative fetal compartment, *n* = 9). We excluded samples 10F and 10M due to potential fecal contamination. Data from 13F and 8F were filtered out due to low alignment rates with the human genome. Data from case 15 were excluded from the analysis due to a long interval from infection to delivery. To identify the impact of COVID-19 on placental function, we further analyzed the data using the bioinformatics software Partek^®^ Flow^®^ software, v9.0 (Chesterfield, MO, USA), to identify the differentially expressed genes (DEGs) in both the maternal and fetal placental compartments of the women with COVID-19. We did not find any differentially expressed genes in the fetal compartment. However, in the maternal compartment of the placenta, a total of 635 DEGs were identified in the COVID-19-positive group compared with the healthy group. In this, 518 genes were upregulated and 117 were downregulated, as displayed in the volcano plot (Figure 1, Supplementary Table S1). The top 10 DEGs are shown in Table 2. Among the maternal DEGs, seven genes belonging to the pregnancy-specific glycoprotein (PSG, Figure 2) family showed significant downregulation with active SARS-CoV-2 infection (fold change range from −13.70 to −5.28; false discovery rate (FDR) ≤ 0.01). Olfactory Receptor Family 5 Subfamily AS Member 1, Ring Finger Protein 212, Olfactory Receptor Family 51, 13, 10, Claudin 18, Neurotrophic Receptor Tyrosine Kinase 3, Cyclin and CBS Domain Divalent Metal Cation Transport Mediator1, Collagen Type XIII Alpha 1 Chain, and Leucine-Rich Repeat Containing 38 were upregulated. Meanwhile, Chorionic Gonadotropin Subunit Betas 5 and 8, Epstein–Barr Virus-Induced 3, Tissue Factor Pathway Inhibitor 2, Corticotropin-Releasing Hormone, Growth Differentiation Factor 15, KiSS-1 Metastasis Suppressor, Pregnancy-Specific Beta-1-Glycoproteins 1 and 9, and Placental Alkaline Phosphatase were downregulated. Further, we conducted a KEGG pathway enrichment analysis to identify the major altered pathway, and the results revealed that oxidative phosphorylation is the prominent pathway that showed significant alterations. To gain further insights into the functional implications of the altered pathway, we performed a gene enrichment analysis. Table 3 displays the results of this analysis. In brief, the following pathways were altered in the maternal compartments of the placentas from women with COVID-19: Oxidative phosphorylation, insulin secretion, cortisol synthesis and secretion, estrogen signaling, antigen processing and presentation, GnRH secretion, endocrine and other factor-regulated calcium reabsorption, oxytocin signaling, fatty acid elongation, GnRH signaling, apelin signaling, and growth hormone synthesis, secretion, and action.

### 2.4. Differentially Expressed Genes in Symptomatic Women Compared with Healthy Women

**Figure 3:**
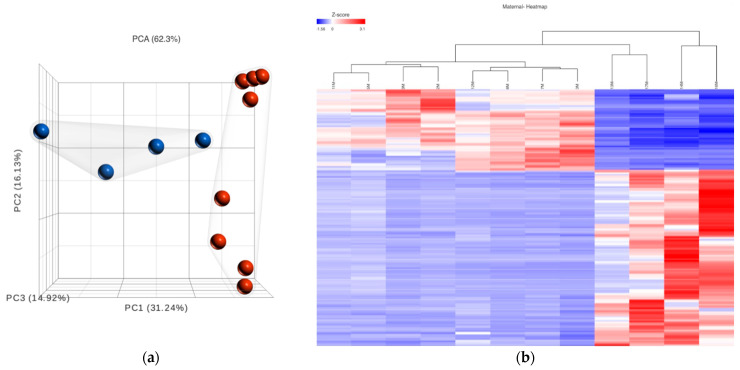
Principal component analysis (PCA) and heatmap visualization of differentially expressed genes (DEGs). (**a**) The PCA plot of DEGs (1788 genes) reveals a tight clustering of healthy placenta samples (depicted by red clustered bubbles), indicating homogeneity within the healthy placenta group. In contrast, the gene expression profile of the placental samples from individuals with COVID-19 (represented by blue bubbles) exhibits a distinct pattern that differs from the healthy group. (**b**) The heatmap provides a visual representation of the expression levels of the DEGs, effectively highlighting their varying expression patterns across maternal placental samples derived from SARS-CoV-2-positive and symptomatic women (depicted in the four panels on the right: 13M, 17M, 14M, and 16M) and samples from healthy women (displayed in the eight panels on the left: 11M, 5M, 2M, 9M, 12M, 8M, 7M, and 3M).

To study the impact of COVID-19 on the maternal placental compartments of women with symptoms of COVID, we compared maternal placental gene expression between women symptomatic for COVID-19 at the time of delivery (*n* = 4) with healthy women (*n* = 8). Using Partek, we identified a total of 1788 differentially regulated genes (FDR < 0.01), and the complete list of these genes can be found in Supplementary Table S2. A principal component analysis (PCA) and heatmap of the DEGs showed a clear demarcation in the gene expression pattern in the maternal compartment of the placenta between women with symptomatic COVID-19 and healthy women (Figure 3a,b). Among the DEGs, PSG1 was significantly downregulated by more than 300 folds in the symptomatic cases, while IGF2 showed a substantial upregulation. Additionally, other PSG genes, *PSG3*, *PSG4*, *PSG6*, *PSG8*, *PSG9*, and *PSG11*, were highly downregulated in the symptomatic cases. Furthermore, the expression of the inflammatory cytokine 6 (*IL-6*) ranged from 2.2- to 22.56-fold higher in the symptomatic patients compared to the healthy women. We further confirmed the findings of the RNA-seq using a real-time PCR and found the levels of expression for *OR4K13*, *OR5M1*, *PSG3*, and *PSG9* were similar to our RNA-seq results (Supplementary Figure S1). Though the real-time PCR results followed the trend of the RNA-seq results, they did not achieve any statistical significance, and this can be explained well by the low sample size and high degree of variation in the symptoms of the COVID-19-positive women.

### 2.5. Comparison of Pathway Enrichment Analysis between Symptomatic and Healthy Women

**Figure 4:**
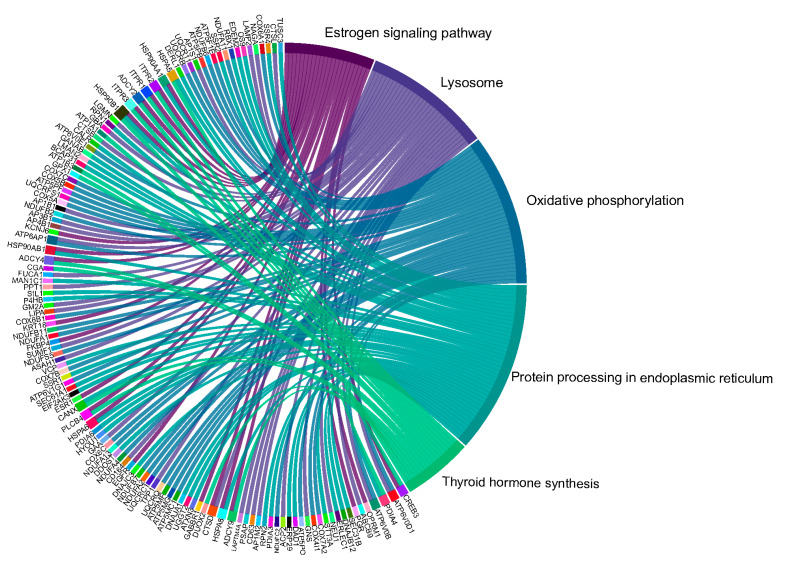
Circos plot showing DEGs and their relevant pathways. The maternal placental compartment in COVID-19-infected and symptomatic women at the time of delivery show alterations in the estrogen signaling pathway, lysosome, oxidative phosphorylation, protein processing in the endoplasmic reticulum, and thyroid hormone synthesis pathways.

**Figure 5:**
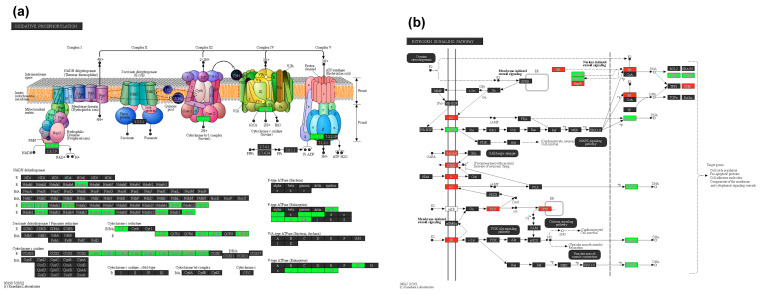

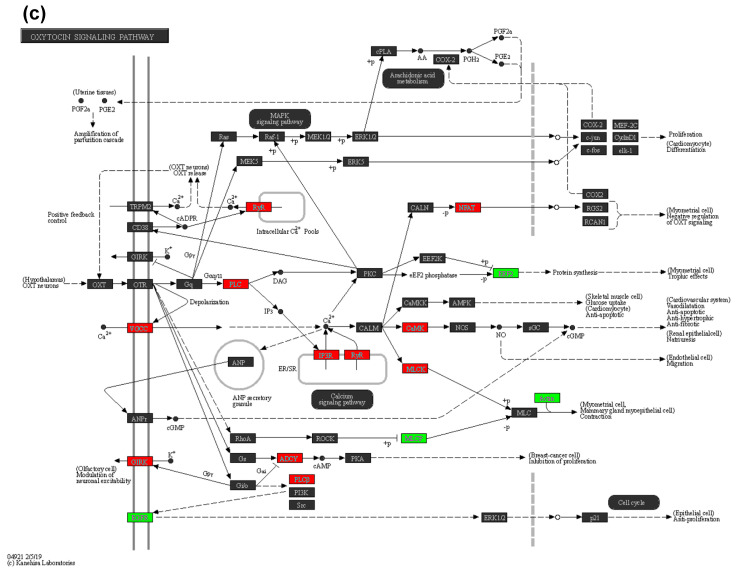
Oxidative phosphorylation (**a**), estrogen (**b**), and oxytocin (**c**) signaling pathways are altered with COVID-19. A KEGG analysis explains the pathways relevant to the clinical symptoms of COVID-19 during pregnancy. Alterations in oxidative phosphorylation (please note all the DEGs are downregulated). Genes marked in red and green are, respectively, up- and downregulated > 2 folds.

Upon conducting a further deep analysis, we identified several notable results from a comparison of pathway enrichment analyses performed between the maternal placentas of women symptomatic for COVID-19 and healthy women. The important pathways that are altered are presented in Table 3, and a Circos plot (Figure 4) presents the DEGs related to the important pathways relevant to placental function. This includes the oxidative phosphorylation (FDR; *p* < 0.001, Figure 5a), insulin secretion (FDR; *p* < 0.001), cortisol synthesis and secretion (FDR < 0.002; *p* < 0.05), antigen processing and presentation (FDR < 0.002; *p* < 0.05, Supplementary Figure S2), estrogen signaling (FDR < 0.002; *p* < 0.05, Figure 5b), GnRH secretion (FDR < 0.01), and oxytocin signaling pathways (*p* < 0.05; Figure 5c).

## 3. Discussion

Our primary aim was to investigate the potential vertical transmission of the SARS-CoV-2 virus from the mother to the fetus through the placenta. We conducted RT-PCR analyses separately on the maternal and fetal compartments of the placenta with the goal of detecting any presence of the SARS-CoV-2 virus. Our findings revealed that there were no detectable levels of the SARS-CoV-2 virus in either the maternal or fetal compartments. Furthermore, our study delved into a comprehensive examination of the impact of COVID-19 on the human placenta, focusing on global transcriptomics with the aim of studying the molecular pathways relevant to clinical reports. We specifically designed the study to analyze gene expression and pathways within the maternal and fetal placental compartments separately. Our pathway enrichment analysis revealed that SARS-CoV-2 had a substantial impact, affecting key pathways such as the oxidative phosphorylation, insulin secretion, estrogen signaling, antigen processing and presentation, and oxytocin pathways relevant to the clinical presentations specific to COVID-19, its pregnancy-related complications, and preeclampsia [12,13].

In our study, we could not detect notable viral loads in the maternal and fetal compartments of the placentas of women who tested positive for COVID-19 via a nasal swab and had mild-to-moderate symptoms. Similar findings using the whole placenta have been published by different studies and thus show a remote possibility of vertical transmission in such groups of patients [14,15]. Though the exact mechanism is not known, there have been studies showing the activation of placental T cells and myeloid cells [15,16]. Considering the complexity of the human placenta, we speculate that another potential mechanism of placental resistance to the SARS-CoV-2 virus may be due to its intrinsic property of a high level of RNase activity. Thus, a high level of RNase activity may offer protection against invading viruses including the SARS-CoV-2 virus, which is a single-stranded RNA genome [17]. This specific characteristic reflects a more complex view of the host defense system and the protective effect of the human placenta toward the fetus. In the context of the presented research, despite the absence of a detectable viral presence within the placental tissue, a significant impact of the virus was discernible at the transcriptional scale, culminating in the functional debilitation of cellular activities in the maternal placenta. A noteworthy alteration observed was the diminished expression of human chorionic gonadotropin (hCG) in placental cells subjected to viral infection. This phenomenon aligns with the findings delineated by Chen et al. [18], which were corroborated through in vitro experimentation.

A meta-analysis shows that pregnant women with SARS-CoV-2 infection face a greater risk of maternal morbidity compared to those without the infection due to vascular dysfunction, namely preeclampsia, thromboembolic disease, and hypertensive disorders of pregnancy [19]. However, few studies focused on the molecular alterations that occur in different compartments of the placenta which are responsible for the above pathophysiological conditions with SARS-CoV-2. Here, we systematically explored the gene expression and pathways affected by SARS-CoV-2 in the maternal compartment of the placenta around the time of delivery. Upon comparing gene expression in the maternal compartment of the placenta between symptomatic women and healthy women, we found that pathways related to estrogen, insulin and cortisol secretion, growth hormone synthesis, and oxytocin were affected. This finding of altered molecular pathways may provide some understanding as to the molecular mechanisms related to clinical symptoms reported, such as preeclampsia and preterm birth, in women with COVID-19 infection [19]. The asymptomatic women in our study did not show many changes in their placental gene expression compared with the healthy group. It can be speculated that this may be attributed to the low severity of the infection, reflecting less pronounced changes in molecular expression that were not enough to cause clinical symptoms. Moreover, within the symptomatic patients, we observed a spread of overall gene expression, as indicated by a PCA analysis, as they had different degrees of symptoms. Our pathway enrichment analysis indicated oxidative phosphorylation as the leading altered regulated pathway, with 37 out of 82 genes differentially regulated, with an enrichment score of 24.5. To our surprise, we found all the 37 DEGs were downregulated and mainly belonged to the ATP synthase, COX, NDUFA4, and UQCRFS1 families. The placenta produces about 5 μmol of ATP/gram of placental tissue/minute [20] as there is a great demand for energy to meet its high metabolic activity. Compromised oxidative phosphorylation may lead to mitochondrial dysfunction [21]. Under similar conditions, COX expression is reduced, as we report in this study [22].

In our study, we found that 20 genes related to the estrogen signaling pathway were differentially expressed, and this includes the expression of estrogen receptor (ESR) and ESR-related receptor beta (ESRRB), with changes in upregulation of 7.5 and 20 folds, respectively. It has been observed that the mRNA and protein for ESR are over-expressed during preeclampsia [13]. A change in ER signaling may lead to increased levels of inflammatory cytokines, affecting the hypoxia-related signaling pathway [23].

Here, we show that the antigen processing and presentation pathway is significantly upregulated in the maternal compartments of the placentas of symptomatic women. This may explain the immune response of the placenta in protecting the fetus from the infection, in line with [24]. The MHC class 1-mediated antigen-presenting pathway has an important role in antiviral immunity. Our study supports the report that MHC class 1 is targeted by the SARS-CoV-2 virus as noticeable downregulation of it during infection occurs [25]. Also, with inhibition, CD74, which belongs to the above pathway, may have an adverse effect on pregnancy [24]. The oxytocin pathway plays a pivotal role in normal pregnancy and in our study, we observed a significant enrichment score for this pathway (*p* < 0.03) in the maternal placental compartments of the COVID-19-symptomatic women, though it did not attain significance with the FDR. Several key molecules in this pathway, namely ACTG1, calcium voltage-gated channel genes (CACNAs), EEF2, EGFR, ITPRs, PLCB4, RYR, and PPP1CA, are differentially regulated.

Placenta-specific glycoproteins (PSGs) regulate placental immune function. The expression of PSGs in the placenta increases along with the gestational week and reaches its peak in late pregnancy [26]. Low maternal levels of PSGs are seen during preeclampsia [27]. Our study showed a decrease in PSG (PSG1-6, 7, 8, 9, 11) levels with COVID-19 in the maternal placenta. This may explain some of the causes of the observed clinical risks of pregnancy-related complications. Particularly noteworthy is case 16, who was clinically reported for PIH and had a moderate COVID-19 infection with symptoms. It must be noted that this woman had a 22.5-fold increase in IL-6 expression in the maternal compartment of the placenta. Our findings on the downregulation of PSGs in placentas of women with SARS-CoV-2 are in line with the first-time report by Gao et al. [28]. They further explained the cause for the altered expression of PSGs and showed that the dysregulation of enhancers originating from Long Terminal Repeat 8B (LTR8B) may be a pivotal factor in the suppression of PSG expression within the syncytiotrophoblasts of COVID-19 patients. A high-throughput chromosome conformation capture analysis substantiated the presence of robust higher-order chromatin interactions between these retrotransposons and multiple PSG promoters, suggesting the existence of potential regulatory modules for PSGs.

A recent report suggests that fetal demise tends to occur a few days after maternal infection with SARS-CoV-2, leading to the development of placental inflammatory lesions connected to the viral infection [22]. Studies also report that unique inflammatory responses in the placenta initiated by SARS-CoV-2 infection can eventually lead to harmful consequences for the mother and neonate [22]. In relation to the above, we observed high levels of inflammatory cytokines belonging to the IL, TNF, TGF, IGF, and IGFBP families, in the maternal placental compartments of individuals with SARS-CoV-2 infection.

It is reported that chorionic gonadotropin peptides are derived from trophoblast cells and play a role in normal pregnancy [29]. Chorionic gonadotropin subunits CGB5 and GCB8 both exhibited significant downregulation in the COVID-19-positive women. Corticotropin-Releasing Hormone, another vital hormone for normal pregnancy, sharply increases toward the end of pregnancy [30]. Low Tissue Factor Pathway Inhibitor 2 is observed in preterm labor [31], and high levels of Growth Differentiation Factor 15 are typical in normal pregnancy [32]. In our study, the above molecules are markedly downregulated in the maternal compartments of placentas from women with symptoms of COVID-19. Olfactory Receptor Family genes (*ORSAS1*, *OR51M1*, and *OR4K13*) are among the top DEGs. While OR5 was previously reported in the female reproductive system [33], a comprehensive literature search failed to find a clear link between the placental expression of olfactory receptors and COVID-19. This intriguing finding warrants further exploration.

Though there are many studies including single-cell RNA sequencing conducted to understand the impact of COVID-19 in term placentas [34], this is the first study performed systematically to understand the impact of COVID-19 on the maternal and fetal compartments separately. Though this study has limitations with a sample size of four in the symptomatic group, we overcame this by applying a stringent FDR cutoff (0.01) and a statistical test based on the Wald test, making the data robust. We find the molecular and pathway-based results in this study to be well aligned with reported clinical observations. In order to reinforce the robustness of our findings obtained from the RNA-seq analysis, we performed real-time PCR validation for four pivotal genes, namely *OR4K13*, *OR5M1*, *PSG3*, and *PSG9*. The selection of these genes was based on their significant differential expression levels, as identified in our study. Our real-time PCR validation substantiates the consistency of the results obtained through the RNA-seq analysis. We also found all the 742 DEGs present in the maternal placentas of the COVID-19-positive women were also part of the DEGs in the symptomatic women. Moreover, the results from our study are in line with those of a very recently published single-nuclear-transcriptome study of placental cells which showed significant downregulations of immune-modulating proteins, PSGs, and other cytokines affecting vascular function [28].In our study, we see a 1.74-fold upregulation in the expression of *VEGFA*. Nevertheless, it is important to acknowledge certain disparities in the findings between the two studies. These variances may stem from differences in the methodology used, the specific placental cell types studied, the extent of infection, and the immune response to infection.

The observed gene expression patterns exhibited notable parallels with those seen in preeclampsia, a condition known to be associated with heightened risk during pregnancy in the context of COVID-19 [12,13,22,26]. This correlation is underscored by the fact that within the study cohort, one patient was diagnosed with gestational diabetes mellitus, while another experienced pregnancy-induced hypertension. Both of these conditions have been linked to a more severe maternal response to COVID-19 [35,36]. Furthermore, these patients demonstrated elevated levels of inflammatory markers, notably interleukin-6 (IL-6). This finding underscores the necessity of conducting more extensive studies, incorporating a larger sample size with such confounding factors. Such research is vital to the development of targeted, patient-specific treatments for COVID-19, especially in cases complicated by concurrent conditions like gestational diabetes and pregnancy-induced hypertension.

The biological pathways derived from the in silico analysis may require further validation with functional assays. A study with a larger number of samples with information about COVID-19 severity, duration of infection, viral load, pregnancy-related diseases, and other co-morbidities will be important as they affect the transcriptomic signature of the placenta and alter biological functions to a different extent. This information will be essential in managing COVID-19 in pregnant women with specific disease conditions.

In summary, apart from supporting the earlier finding that SARS-CoV-2 is not detectable in the term placentas of women with low or medium symptoms, our study sheds more light on understanding the effect of COVID-19 on the placenta at molecular level and its related functional pathways, explaining the clinical challenges faced in instances of COVID-19 during pregnancy. We observe that the identified molecular changes are predominantly in the maternal placental compartments of women with symptoms of COVID-19. This information may provide important information for the clinical management of pregnant women with SARS-CoV-2 infection.

## 4. Materials and Methods

### 4.1. Study Population

This study was approved by the National Ethics Authority (EPM) in Sweden (Dnr: 2020-01938). In total, 16 pregnant women in the 3rd trimester of pregnancy were recruited after providing informed consent. The study’s general inclusion criteria consisted of women admitted for delivery in their 3rd trimester of pregnancy who had a normal, healthy, and viable singleton pregnancy. The exclusion criteria encompassed a history of preeclampsia, any known reproductive tract infections, known immune disorders, and any regular medications for long-term illness. Specific inclusion criteria for healthy controls were a negative PCR test report for COVID-19, and the exclusion criteria were a positive COVID-19 test during the last 3 months of pregnancy. Conversely, for the COVID-19-positive group, the inclusion criteria encompassed a positive PCR test for COVID-19 within four days of delivery. All participants were recruited before vaccination was available. None of the subjects were vaccinated for SARS-CoV-2. Before delivery, 7 women were positive for SARS-CoV-2 infection, while 9 women were negative for SARS-CoV-2 during the 3rd trimester of pregnancy. COVID-19 infection was confirmed with a nasal swab via an RT-PCR. Clinical data for all positive women were collected. This study was conducted in accordance with the Declaration of Helsinki, and this protocol was approved by the local Ethics Committee. No connection to the patients’ identities was conceivable because all the data were anonymized.

### 4.2. Sample Collection

Fresh placental tissues were collected within 30 min of delivery according to the previous literature [37]. All placenta weights were within a normal range. Potential contamination with feces in sample 10 was observed at the time of collection; therefore, sample 10 was excluded. For each placental sample, one piece of the maternal compartment (approximate dimensions of 1 cm × 0.5 cm × 0.5 cm) was dissected from the middle of the parenchyma. The amniotic membrane was removed from the fetal side, and another piece of placental tissue was cut from the fetal compartment. To remove blood, the tissue cubes were washed with sterile phosphate-buffered saline (Gibco, Carlsbad, CA, USA) and divided into three parts. The first part of the placental sample was placed into a cryovial, immediately snap-frozen with dry ice, and stored in liquid nitrogen for further processing. The second part of the sample was placed into a 2 mL cryotube with 1.5 mL RNAlater (Invitrogen, Carlsbad, CA, USA) and stored at −80 °C. To prepare a formalin-fixed paraffin-embedded tissue block, the third part of each sample was fixed with a 4% paraformaldehyde solution (Sigma, Darmstadt, Germany) in a 5-mL Eppendorf tube for 24 h; the process continued with serial dehydration using ethanol, clearing, paraffin infiltration, and embedding as previously described [38], and the samples were later stored at 4 °C for future experiments.

### 4.3. RNA Extraction

A Quick-RNA Microprep Kit (Zymo Research, Irvine, CA, USA, Catalogue No: R1050) was used to extract total RNA from the snap-frozen placental tissues, following the manufacturer’s protocol, with a proteinase K treatment [39]. Briefly, approximately 3 mg of placental tissue was chopped into 0.2 mm^3^ pieces on ice and transferred to a tube containing 600 µL of an RNA lysis buffer with proteinase K reaction mix added. After incubating the tube for one hour at room temperature, the tissue pieces in the RNA lysis buffer were homogenized in a 2 mL ZR BashingBead Lysis Tube (Zymo Research, Irvine, CA, USA) using a Disruptor Genie^®^ (Zymo Research, Irvine, CA, USA) for 30 min. All the samples were DNAse-treated to remove genomic DNA contamination in the downstream application. RNA quantification was measured using a Qubit Flex Fluorometer (Thermo Fisher Scientific, Waltham, MA, USA), using a Qubit high-sensitivity RNA assay kit (Thermo Fisher Scientific, Waltham, MA, USA).

### 4.4. qPCR for SARS-CoV-2 Detection

A real-time fluorescent RT-PCR kit for detecting SARS-CoV-2 (Beijing Genomics Institute, Shenzhen, China, MFG030015) was used to detect the ORF1ab and N genes of SARS-CoV-2 using a reverse-transcription PCR. According to the manufacturer’s instructions, 10 µL of extracted RNA from each sample was added to the reaction system and mixed with 18.5 µL of a reaction mix and 1.5 µL of an enzyme mix. The total reaction volume was 30 µL. RT-qPCR cycling was conducted on a Bio-Rad CFX Connect PCR system (Bio-Rad, Hercules, CA, USA) as follows: 50 °C for 20 min, 95 °C for 5 min, and then 45 cycles of 15 s at 95 °C and 30 s at 60 °C. The testing result was interpreted as positive if the standard curves were in an S-shape with cycle threshold (Ct) values ≤ 40. The specimen was determined to be negative if standard curves were not in an S-shape and had no Ct values or Ct > 40. The BGI assay has a limit of detection of 100 copies/mL.

### 4.5. mRNA Library Preparation and Sequencing

DNA libraries for next-generation sequencing were constructed from all the placental sample RNA using the Smart-seq2 protocol [40] with 30 ng of total RNA for each sample. The enzymatic fragmentation and tagmentation of DNA were performed using the Nextera XT kit (Illumina Inc., San Diego, CA, USA) along with IDT^®^ for Illumina^®^ DNA Unique Dual Index barcodes. The final amplified libraries were purified with AMPure XP beads at a ratio of 1:1 (sample versus beads). The final cDNA library was quantified using a Qubit 1X HS DNA assay kit (Thermo Fisher Scientific, Waltham, MA, USA), and the quality of the libraries was analyzed using a 2100 Bioanalyzer system (Agilent, Santa Clara, CA, USA) with a high-sensitivity DNA chip (Agilent, Santa Clara, CA, USA). An equimolar concentration of 5 ng from each library was pooled. Sequencing was performed on the Illumina NovaSeq 6000 sequencing platform (Novogene, Cambridge, UK) with 2 × 150 bp read setup.

### 4.6. Sequencing Data Processing and Bioinformatic Analysis

#### mRNA Data Analysis

Raw sequencing data processing and analysis for both mRNA and small RNA transcriptomes was conducted using the Partek^®^ Flow^®^ platform (version 10.0.22.1005). A raw data quality check was performed using the FastQC (version 0.11.9) toolkit in the Partek^®^ Flow^®^ platform. Briefly, for mRNA sequencing, the Nextera adapters were trimmed, and the trimmed reads were aligned to hg38 using a STAR aligner with default parameters. Sixty percent of the alignment rate was taken as the cutoff value. After alignment, samples 8F and 13F were removed due to low alignment rates (8F, 57.29%; 13F, 39.44%). The aligned reads were quantified to Ensembl Transcripts release 107. Mitochondrial and ribosomal reads were filtered out afterward, and genes with a count of <1 in at least 80% of the samples were filtered out. The data were normalized using the median ratio method to avoid variations in the read counts in the samples (Supplementary Figure S3). For the downstream analysis, we split the data by placental surface (maternal and fetal). On the maternal side, 6 samples from the SARS-CoV-2-positive group and 8 samples from the healthy group were included. On the fetal side, 6 samples from the SARS-CoV-2-positive group and 7 samples from the healthy group were included in the final analysis. A differential expression analysis of the filtered counts was performed using the DESeq2 tool (version 3.5). We excluded genes with low levels of expression in all samples that had less than 10 counts. A false discovery rate (FDR) of ≤0.01 and fold changes (FCs) of <−2 or >2 were considered statistically significant. The KEGG (Kyoto Encyclopedia of Genes and Genomes) database [41] was used to perform a pathway analysis of DEGs, and *p* < 0.05 was considered significant for enriched biological signaling pathways.

### 4.7. Validation of RNAseq Data by qPCR

The RNA extracted from the snap-frozen tissues was utilized to validate the differential gene expression through a real-time PCR. cDNA synthesis was carried out using the SuperScript VILOTM kit (Invitrogen, Thermo Fisher Scientific, Waltham, MA, USA). Subsequently, triplicate qPCR reactions were performed with the TaqMan expression probes IL-6 and PSG3 (Thermo Fisher Scientific), employing 10 ng of cDNA. Real-time PCR experiments were conducted on a StepOne Plus Instrument (Applied Biosystems, Foster City, CA, USA), utilizing TaqMan Universal PCR Master Mix (Applied Biosystems). Each sample was run in triplicate, and 18s rRNA was selected as the housekeeping gene for normalization purposes. The ΔCt value was calculated by normalizing the target gene expression to the levels of 18s rRNA. Subsequently, fold-change values in the COVID-19-positive maternal placental samples were determined by comparing the results with the healthy maternal placental samples, using the standard formula 2^−ΔΔCT^.

## 5. Conclusions

In summary, this study provides further evidence that SARS-CoV-2 infection with mild symptoms may not result in a detectable placental virus load in either the maternal or fetal compartment. This further supports the belief that mild infection is unlikely to transmit the virus during late pregnancy. More importantly, this study sheds light on the possible molecular regulation and pathways associated with an increased clinical risk of preeclampsia and preterm birth with COVID-19 during pregnancy. The findings from this study offer valuable insights that could be further explored clinically in managing pregnant women with COVID-19.

## Figures and Tables

**Table 1:** Clinical characteristics of SARS-CoV-2-positive women (*n* = 7).

Case	Maternal Age (Years)	Gestational Age (Weeks + Days)	Delivery Mode	Maternal Co-Morbidities	Long-Term Medication, If Any	COVID-19 Status	Time from SARS-CoV-2-Positive Result to Delivery (Days)	Severity of COVID-19 at the Time of Delivery	Placental Pathology	Gender	Newborn Apgar Score (1, 5, 10 min)	Neonate Symptoms/ Complications
1	32	40 + 3	Vaginal	GDM ^a^	Metformin	Active ^c^	0	Asymptomatic	Normal	Female	10-10-10	Healthy
4	41	41 + 2	C-section	-	-	Active ^c^	1	Asymptomatic	Normal	Male	08-08-10	Respiratory distress syndrome
13	31	34 + 5	C-section	GDM, Placenta accreta	-	Active ^c^	4	Mild: cough	Placenta accreta	Male	09-09-09	Healthy
14	35	39 + 4	Vaginal	-	-	Active ^c^	0	Mild: cough	Normal	Male	09-10-10	Healthy
15 *	32	37 + 5	C-section	-	-	Past ^d^	110	Asymptomatic	Normal	Female	09-10-10	Respiratory distress syndrome
16	31	40 + 5	Vaginal	-	-	Active ^c^	1	Mild: cough; chills	Normal	Male	08-09-10	Healthy
17	37	39 + 3	C-section	PIH ^b^, Polycystic kidney	-	Active ^c^	4	Mild: cough; wheezing	Normal	Male	09-10-10	Healthy

^a^ GDM = gestational diabetes mellitus; ^b^ PIH = pregnancy-induced hypertension; ^c^ active = active SARS-CoV-2 infection (≤5 days since diagnosis) at the time of the delivery; ^d^ past = active SARS-CoV-2 infection (>5 days since diagnosis) at the time of the delivery; * Case 15 was excluded from further study due to a long interval from infection to delivery.

**Table 2:** Top 10 up- and down-regulated DEGs (protein coding) between maternal placentas of women symptomatic for COVID-19 and healthy women.

Gene Symbol	Gene Name	Fold Change	FDR
*OR5AS1*	Olfactory Receptor Family 5 Subfamily AS Member 1	108.7	5.92 × 10^−6^
*RNF212*	Ring Finger Protein 212	58.26	3.27 × 10^−5^
*OR51M1*	Olfactory Receptor Family 51 Subfamily M Member 1	53.85	2.71 × 10^−4^
*OR4K13*	Olfactory Receptor Family 4 Subfamily K Member 13	52.39	1.98 × 10^−4^
*CLDN18*	Claudin 18	44.05	3.95 × 10^−3^
*NTRK3*	Neurotrophic Receptor Tyrosine Kinase 3	42.44	2.21 × 10^−5^
*CNNM1*	Cyclin And CBS Domain Divalent Metal Cation Transport Mediator 1	41.80	5.04 × 10^−4^
*COL13A1*	Collagen Type XIII Alpha 1 Chain	41.45	1.02 × 10^−3^
*OR10G3*	Olfactory Receptor Family 10 Subfamily G Member 3	40.21	1.58 × 10^−3^
*LRRC38*	Leucine-Rich Repeat Containing 38	39.64	1.27 × 10^−5^
*CGB5*	Chorionic Gonadotropin Subunit Beta 5	−53.67	3.78 × 10^−3^
*EBI3*	Epstein–Barr Virus-Induced 3	−49.80	7.93 × 10^−7^
*CGB8*	Chorionic Gonadotropin Subunit Beta 8	−38.49	5.67 × 10^−3^
*TFPI2*	Tissue Factor Pathway Inhibitor 2	−35.59	2.72 × 10^−7^
*CRH*	Corticotropin-Releasing Hormone	−34.22	1.51 × 10^−5^
*GDF15*	Growth Differentiation Factor 15	−33.29	5.33 × 10^−7^
*KISS1*	KiSS-1 Metastasis Suppressor	−32.69	6.5 × 10^−3^
*PSG9*	Pregnancy-Specific Beta-1-Glycoprotein 9	−31.00	2.11 × 10^−6^
*PSG1*	Pregnancy-Specific Beta-1-Glycoprotein 1	−28.37	1.47 × 10^−7^
*ALPP*	Alkaline Phosphatase, Placental	−27.94	1.8 × 10^−5^

FDR = false discovery rate.

**Table 3:** Significantly altered pathways in the maternal placentas of women with symptomatic COVID-19 compared to healthy women based on FDR and functional importance.

Description	Enrichment Score	*p*-Value	FDR
Oxidative phosphorylation	24.48	<0.001	<0.001
Insulin secretion	11.01	<0.001	<0.001
Cortisol synthesis and secretion	6.44	0.002	0.028
Estrogen signaling pathway	6.35	0.002	0.029
Antigen processing and presentation	5.97	0.003	0.039
GnRH secretion	4.81	0.008	0.109
Endocrine and other factor-regulated calcium reabsorption	3.82	0.022	0.218
Oxytocin signaling pathway	3.60	0.027	0.257
Fatty acid elongation	3.30	0.037	0.320
GnRH signaling pathway	3.24	0.039	0.320
Apelin signaling pathway	3.01	0.049	0.364

## Data Availability

Sequence data have been deposited in the NCBI’s Gene Expression Omnibus and are accessible through the GEO Series accession number GSE233557 (https://www.ncbi.nlm.nih.gov/geo/query/acc.cgi?acc=GSE233557, accessed on 4 July 2023).

## Supplementary Materials

The following supporting information can be downloaded at: https://www.mdpi.com/article/10.3390/ijms25031608/s1.
