# Supplementary material for: Global Transcriptomic Analysis of Placentas from Women with Gestational SARS-CoV-2 Infection during the Third Trimester of Pregnancy
Source: Int J Mol Sci. 2024 Jan 28;25(3):1608. doi: 10.3390/ijms25031608 (PMC10855544; doi:10.3390/ijms25031608)
Supplement: Supplementary file 1 [file ijms-25-01608-s001.zip › Supplementary figures.pdf]

## *Supplementary figures*

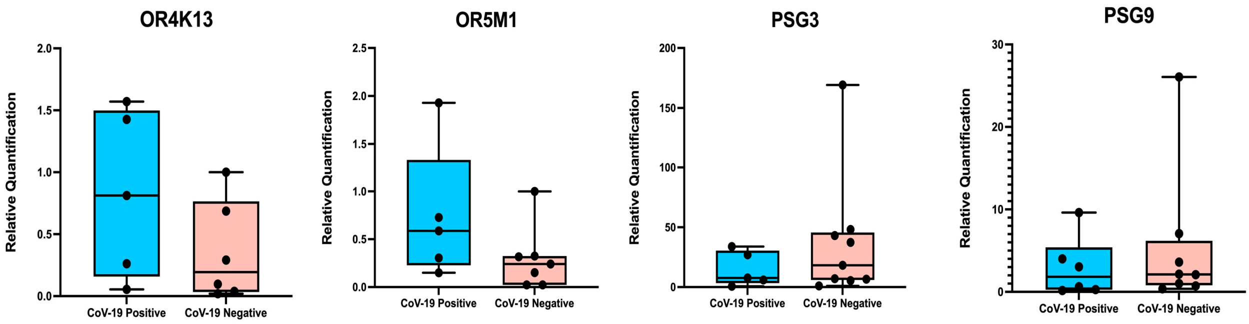


**Figure S1. Validation of RNAseq Data by Real-Time PCR Analysis.** The gene expression levels of OR4K13, OR5M1, PSG3 and PSG9 were analyzed using real-time PCR to confirm the reliability of the RNAseq data. The results demonstrate a concordant expression pattern between the two methods, providing validation for the RNAseq-based gene expression analysis.

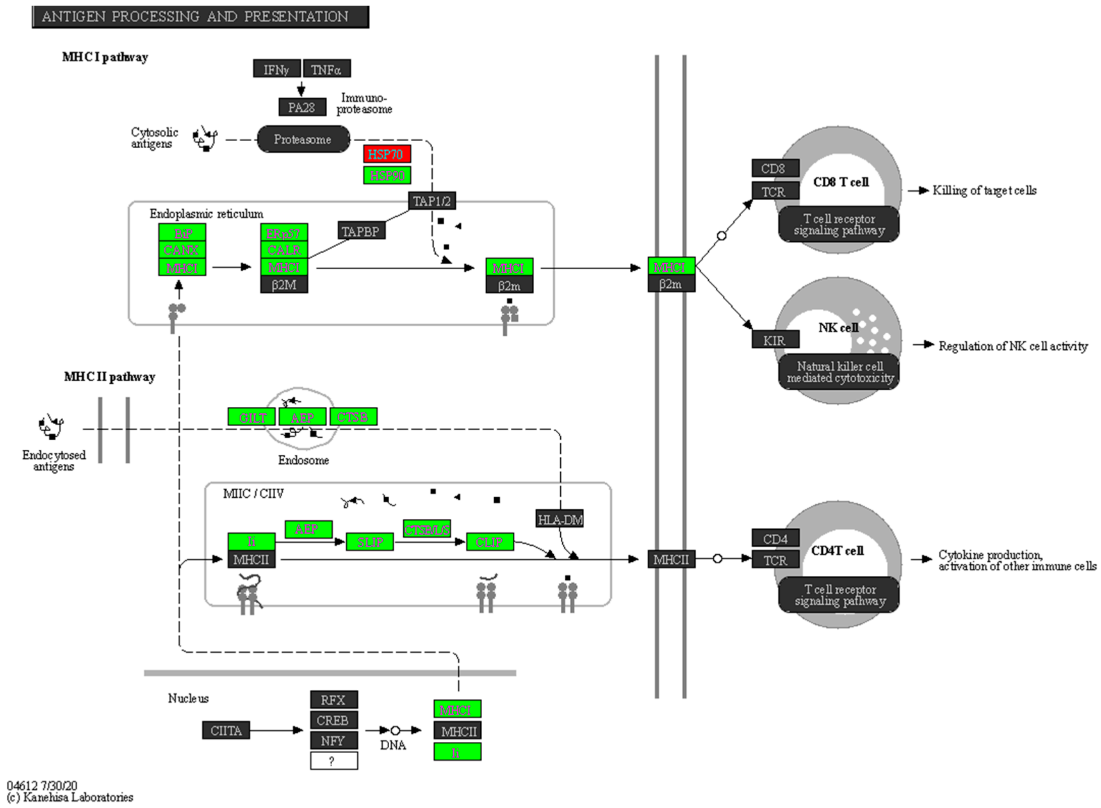


**Figure S2. Antigen processing and presentation signaling pathway is altered with COVID-19**. KEGG analysis explains the pathways relevant to the clinical symptoms of COVID-19 during pregnancy. It is to be noted that most of the DEG are down regulated > 2 folds (in green) in maternal compartment of placenta with Covid-19.

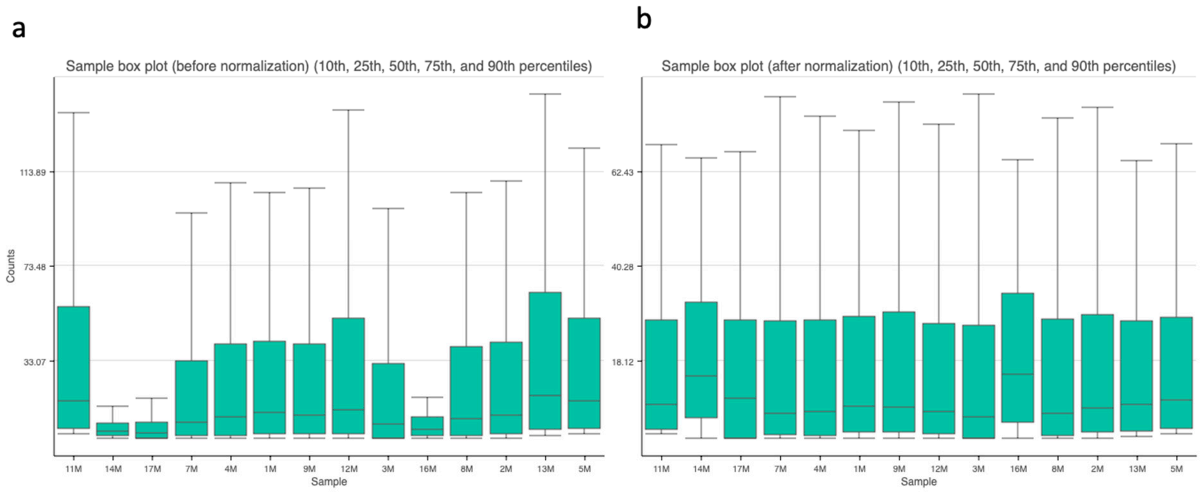


**Figure S3.** Samples with low reads (**a**) were normalised using median range normalization method using Partek. Post normalisation (**b**), the normalized read counts demonstrate a consistent and uniform distribution.
