# Supplementary material for: Global Transcriptomic Analysis of Placentas from Women with Gestational SARS-CoV-2 Infection during the Third Trimester of Pregnancy
Source: Int J Mol Sci. 2024 Jan 28;25(3):1608. doi: 10.3390/ijms25031608 (PMC10855544; doi:10.3390/ijms25031608)
Supplement: Supplementary file 1 [file ijms-25-01608-s001.zip › Supplementary Table S1- positive vs healthy.pdf]

Supplementary table

Differentially Expressed Genes in the Maternal Placental Compartment among Women with COVID-19 Compared to Healthy Controls. Analysis Utilized Stringent DEG Criteria with FDR of 0.01. Genes are Presented in Ascending Order of Fold Change

Gene name	Gene biotype	P-value (Positive vs Negative)	FDR step up (Positive vs Negative)	Fold change (Positive vs Negative)
PSG9	protein_coding	2.38E-06	1.63E-03	-1.37E+01
ALPP	protein_coding	1.83E-04	1.70E-02	-9.88E+00
PSG3	protein_coding	2.59E-04	1.88E-02	-8.40E+00
MAL	protein_coding	1.41E-03	4.06E-02	-6.54E+00
NDUFC2-KCTD14	protein_coding	3.74E-04	2.18E-02	-6.46E+00
PSG6	protein_coding	4.60E-04	2.40E-02	-6.34E+00
PSG2	protein_coding	2.86E-04	1.95E-02	-5.92E+00
PSG10P	transcribed_unprocessed_pseudogene	4.87E-04	2.47E-02	-5.90E+00
GPC3	protein_coding	6.54E-05	1.06E-02	-5.88E+00
CSH2	protein_coding	6.80E-04	2.90E-02	-5.54E+00
PSG5	protein_coding	1.81E-03	4.46E-02	-5.48E+00
VNN2	protein_coding	3.89E-04	2.23E-02	-5.42E+00
HSPB1	protein_coding	9.38E-04	3.30E-02	-5.34E+00
PSG4	protein_coding	1.18E-03	3.71E-02	-5.28E+00
GH1	protein_coding	2.10E-03	4.82E-02	-4.99E+00
PPID	protein_coding	4.54E-04	2.39E-02	-4.77E+00
PEX10	protein_coding	1.24E-03	3.81E-02	-4.26E+00
S100P	protein_coding	1.11E-03	3.60E-02	-4.04E+00
FRZB	protein_coding	1.63E-05	5.24E-03	-4.01E+00
VSIR	protein_coding	2.81E-04	1.95E-02	-3.95E+00
LINC02967	lncRNA	1.48E-03	4.09E-02	-3.69E+00
CTSD	protein_coding	1.08E-03	3.56E-02	-3.54E+00
MAP1LC3B2	protein_coding	2.93E-04	1.95E-02	-3.35E+00
SCCPDH	protein_coding	1.28E-03	3.88E-02	-3.31E+00
H4C3	protein_coding	1.70E-04	1.64E-02	-3.21E+00
LGMN	protein_coding	7.22E-04	2.97E-02	-3.13E+00
SLC39A6	protein_coding	8.33E-04	3.13E-02	-3.10E+00
COX6A1P2	processed_pseudogene	3.14E-04	1.98E-02	-3.09E+00
SQSTM1	protein_coding	8.06E-04	3.10E-02	-3.01E+00

HSPB8	protein_coding	1.75E-03	4.38E-02	-2.94E+00
SVBP	protein_coding	1.25E-03	3.83E-02	-2.93E+00
ATOX1	protein_coding	1.22E-05	4.53E-03	-2.86E+00
ECM1	protein_coding	2.14E-03	4.87E-02	-2.84E+00
S100A6	protein_coding	1.30E-06	1.06E-03	-2.84E+00
GABARAPL1	protein_coding	4.48E-05	8.78E-03	-2.84E+00
DNAJB9	protein_coding	1.75E-03	4.38E-02	-2.80E+00
CCDC47	protein_coding	7.23E-04	2.97E-02	-2.78E+00
CHID1	protein_coding	1.07E-03	3.53E-02	-2.72E+00
CSF1R	protein_coding	2.26E-03	4.98E-02	-2.71E+00
COX6C	protein_coding	1.24E-03	3.81E-02	-2.70E+00
KDELR1	protein_coding	2.34E-04	1.80E-02	-2.68E+00
ATP6V0B	protein_coding	5.52E-04	2.63E-02	-2.68E+00
H2AZ1	protein_coding	2.23E-03	4.96E-02	-2.66E+00
HTATIP2	protein_coding	6.83E-04	2.90E-02	-2.65E+00
DEPP1	protein_coding	1.42E-05	4.93E-03	-2.59E+00
HACD3	protein_coding	5.02E-06	2.52E-03	-2.58E+00
LITAF	protein_coding	2.23E-03	4.96E-02	-2.58E+00
SURF4	protein_coding	3.59E-04	2.14E-02	-2.57E+00
SLC39A7	protein_coding	1.47E-03	4.09E-02	-2.54E+00
EMC7	protein_coding	9.56E-04	3.34E-02	-2.53E+00
PERP	protein_coding	1.71E-03	4.36E-02	-2.51E+00
LRPAP1	protein_coding	1.84E-03	4.49E-02	-2.51E+00
ORMDL3	protein_coding	2.70E-04	1.94E-02	-2.48E+00
DDX49	protein_coding	1.20E-03	3.76E-02	-2.47E+00
TAX1BP3	protein_coding	1.12E-03	3.60E-02	-2.47E+00
TKT	protein_coding	1.21E-06	1.03E-03	-2.46E+00
AP2M1	protein_coding	3.53E-04	2.14E-02	-2.46E+00
PLD3	protein_coding	1.38E-03	4.01E-02	-2.46E+00
TAPBP	protein_coding	1.66E-04	1.64E-02	-2.45E+00
NDUFA4	protein_coding	2.06E-04	1.79E-02	-2.43E+00
PBXIP1	protein_coding	8.29E-04	3.13E-02	-2.41E+00
ATP5F1E	protein_coding	5.60E-05	9.75E-03	-2.40E+00
ARL6IP5	protein_coding	1.49E-03	4.10E-02	-2.37E+00
LAMP2	protein_coding	1.29E-03	3.91E-02	-2.36E+00
ARPC5	protein_coding	3.59E-06	2.03E-03	-2.36E+00
IARS2	protein_coding	3.75E-04	2.18E-02	-2.35E+00
TXNRD1	protein_coding	1.59E-03	4.23E-02	-2.35E+00
SYNGR2	protein_coding	2.18E-03	4.92E-02	-2.34E+00

DAD1	protein_coding	1.25E-03	3.83E-02	-2.34E+00
AKR1B1	protein_coding	1.12E-03	3.60E-02	-2.33E+00
RHOG	protein_coding	2.32E-04	1.80E-02	-2.33E+00
REEP5	protein_coding	1.75E-03	4.38E-02	-2.33E+00
SCAMP2	protein_coding	5.45E-05	9.70E-03	-2.32E+00
C1GALT1	protein_coding	1.35E-03	3.98E-02	-2.32E+00
FIS1	protein_coding	2.96E-04	1.95E-02	-2.32E+00
BLOC1S1	protein_coding	1.85E-04	1.70E-02	-2.31E+00
TMED4	protein_coding	1.58E-03	4.23E-02	-2.31E+00
RNASEK	protein_coding	2.02E-05	6.06E-03	-2.31E+00
CD164	protein_coding	1.03E-03	3.43E-02	-2.31E+00
C8orf76	protein_coding	1.32E-03	3.94E-02	-2.31E+00
APMAP	protein_coding	8.87E-04	3.25E-02	-2.30E+00
CD59	protein_coding	1.56E-03	4.21E-02	-2.30E+00
OAZ1	protein_coding	6.39E-04	2.81E-02	-2.29E+00
SCAMP3	protein_coding	8.02E-04	3.10E-02	-2.29E+00
ATP1B3	protein_coding	2.34E-04	1.80E-02	-2.28E+00
GPAA1	protein_coding	2.74E-04	1.95E-02	-2.28E+00
APEH	protein_coding	1.20E-03	3.76E-02	-2.28E+00
ELOB	protein_coding	5.50E-04	2.63E-02	-2.27E+00
COX7A2	protein_coding	2.84E-07	4.40E-04	-2.26E+00
LCP1	protein_coding	6.79E-04	2.90E-02	-2.25E+00
TMEM141	protein_coding	1.53E-03	4.15E-02	-2.22E+00
NDUFA1	protein_coding	3.85E-04	2.21E-02	-2.21E+00
ANXA6	protein_coding	1.38E-03	4.01E-02	-2.20E+00
APOL1	protein_coding	3.83E-05	8.38E-03	-2.20E+00
HSP90AA1	protein_coding	1.89E-04	1.72E-02	-2.19E+00
PISD	protein_coding	7.65E-04	3.07E-02	-2.19E+00
COX7C	protein_coding	2.91E-04	1.95E-02	-2.18E+00
ITFG1	protein_coding	1.91E-03	4.60E-02	-2.18E+00
NAPA	protein_coding	1.12E-03	3.60E-02	-2.18E+00
EIF1	protein_coding	5.09E-05	9.45E-03	-2.17E+00
ATP6V1E1	protein_coding	1.79E-03	4.43E-02	-2.17E+00
LRRC42	protein_coding	2.82E-04	1.95E-02	-2.16E+00
FAU	protein_coding	1.16E-03	3.69E-02	-2.15E+00
GABARAP	protein_coding	8.50E-04	3.16E-02	-2.13E+00
RPA3	protein_coding	1.23E-03	3.81E-02	-2.12E+00
TMEM254	protein_coding	1.65E-03	4.29E-02	-2.11E+00
PARK7	protein_coding	1.65E-03	4.29E-02	-2.10E+00

COPE	protein_coding	5.90E-04	2.73E-02	-2.09E+00
TM9SF1	protein_coding	9.31E-04	3.29E-02	-2.07E+00
BTF3	protein_coding	1.75E-03	4.38E-02	-2.06E+00
COX4I1	protein_coding	1.77E-03	4.40E-02	-2.06E+00
UQCRFS1	protein_coding	1.43E-03	4.07E-02	-2.06E+00
PTGES3	protein_coding	4.10E-05	8.59E-03	-2.05E+00
ATP6V0D1	protein_coding	1.83E-04	1.70E-02	-2.04E+00
BCAP29	protein_coding	1.57E-03	4.23E-02	-2.04E+00
UQCR11	protein_coding	3.34E-04	2.08E-02	-2.01E+00
TUBB4B	protein_coding	2.48E-04	1.84E-02	-2.00E+00
MAPKAPK5	protein_coding	2.08E-03	4.80E-02	2.00E+00
MPHOSPH8	protein_coding	1.51E-04	1.62E-02	2.01E+00
NBPF1	protein_coding	7.68E-04	3.07E-02	2.01E+00
KANSL3	protein_coding	2.45E-04	1.84E-02	2.02E+00
FAM193B	protein_coding	2.19E-03	4.92E-02	2.03E+00
SPG7	protein_coding	3.08E-04	1.98E-02	2.05E+00
CENATAC	protein_coding	9.02E-04	3.27E-02	2.05E+00
ZSCAN30	protein_coding	7.64E-04	3.07E-02	2.07E+00
LARS1	protein_coding	1.46E-03	4.09E-02	2.08E+00
OGT	protein_coding	5.37E-05	9.68E-03	2.09E+00
LUC7L2	protein_coding	5.17E-04	2.55E-02	2.09E+00
ITSN2	protein_coding	1.01E-03	3.41E-02	2.09E+00
TUT4	protein_coding	5.92E-04	2.73E-02	2.10E+00
DMD	protein_coding	4.58E-04	2.40E-02	2.10E+00
ZNF224	protein_coding	1.68E-04	1.64E-02	2.10E+00
KDM2B	protein_coding	1.75E-03	4.38E-02	2.11E+00
CHD9	protein_coding	4.52E-05	8.78E-03	2.12E+00
SREK1	protein_coding	9.66E-04	3.35E-02	2.13E+00
TAF1D	protein_coding	1.12E-03	3.61E-02	2.13E+00
TEP1	protein_coding	1.83E-03	4.49E-02	2.14E+00
ITPR2	protein_coding	1.11E-03	3.60E-02	2.14E+00
PNISR	protein_coding	9.41E-04	3.30E-02	2.16E+00
NCBP3	protein_coding	2.58E-04	1.88E-02	2.16E+00
HERC1	protein_coding	6.96E-04	2.94E-02	2.16E+00
ERC1	protein_coding	1.32E-03	3.94E-02	2.16E+00
PSD3	protein_coding	2.22E-03	4.96E-02	2.17E+00
RPAP2	protein_coding	1.42E-03	4.06E-02	2.17E+00
TET2	protein_coding	2.78E-04	1.95E-02	2.18E+00
ZBTB40	protein_coding	6.37E-04	2.81E-02	2.20E+00

BEND7	protein_coding	1.70E-03	4.36E-02	2.21E+00
NEMF	protein_coding	1.49E-04	1.62E-02	2.21E+00
SEPSECS	protein_coding	1.43E-03	4.07E-02	2.22E+00
ARID4A	protein_coding	3.69E-04	2.18E-02	2.25E+00
CEP63	protein_coding	1.51E-03	4.12E-02	2.25E+00
KLF8	protein_coding	5.64E-04	2.67E-02	2.26E+00
THADA	protein_coding	1.45E-03	4.09E-02	2.27E+00
PSMG4	protein_coding	1.58E-03	4.23E-02	2.27E+00
MYSM1	protein_coding	1.62E-03	4.24E-02	2.27E+00
FRMD4A	protein_coding	2.75E-05	7.01E-03	2.29E+00
WDR59	protein_coding	1.44E-04	1.61E-02	2.29E+00
PILRB	protein_coding	3.38E-04	2.08E-02	2.30E+00
HERC2P2	transcribed_unprocessed_pseudogene	6.23E-04	2.79E-02	2.32E+00
AFDN	protein_coding	7.27E-04	2.97E-02	2.34E+00
CROCCP2	transcribed_unprocessed_pseudogene	5.31E-04	2.58E-02	2.35E+00
PI4KAP2	transcribed_unitary_pseudogene	1.14E-04	1.40E-02	2.37E+00
DDX10	protein_coding	1.11E-03	3.60E-02	2.37E+00
SIPA1	protein_coding	1.41E-03	4.05E-02	2.37E+00
PDE8A	protein_coding	9.06E-04	3.27E-02	2.40E+00
PARP6	protein_coding	1.29E-05	4.68E-03	2.40E+00
LRCH3	protein_coding	1.48E-03	4.09E-02	2.41E+00
HACE1	protein_coding	1.97E-03	4.68E-02	2.41E+00
ZFP90	protein_coding	1.46E-03	4.09E-02	2.42E+00
CEP170	protein_coding	7.62E-04	3.07E-02	2.42E+00
FAM219B	protein_coding	2.03E-03	4.74E-02	2.43E+00
PRPF38B	protein_coding	1.98E-03	4.68E-02	2.44E+00
CCNL2	protein_coding	2.85E-06	1.80E-03	2.44E+00
TBCK	protein_coding	2.42E-04	1.82E-02	2.45E+00
CSPP1	protein_coding	4.05E-04	2.27E-02	2.45E+00
PHF14	protein_coding	1.35E-03	3.98E-02	2.46E+00
TNK2	protein_coding	9.87E-04	3.37E-02	2.47E+00
MAGI1	protein_coding	1.87E-03	4.55E-02	2.47E+00
JARID2	protein_coding	4.69E-04	2.42E-02	2.48E+00
BCL6	protein_coding	1.71E-03	4.37E-02	2.49E+00
LINC-PINT	lncRNA	4.30E-04	2.32E-02	2.50E+00
FHIP1B	protein_coding	1.99E-03	4.70E-02	2.50E+00

PDE4D	protein_coding	1.59E-04	1.62E-02	2.50E+00
ZNF44	protein_coding	2.58E-04	1.88E-02	2.50E+00
PABPC1L	protein_coding	2.01E-03	4.72E-02	2.52E+00
LRRFIP1	protein_coding	1.06E-04	1.37E-02	2.53E+00
GSDMB	protein_coding	7.70E-04	3.07E-02	2.55E+00
ZNF37A	protein_coding	1.42E-03	4.06E-02	2.55E+00
ANKRD26	protein_coding	1.54E-04	1.62E-02	2.55E+00
DST	protein_coding	2.28E-04	1.80E-02	2.56E+00
NPIPB5	protein_coding	1.12E-04	1.39E-02	2.56E+00
H19	lncRNA	7.50E-04	3.04E-02	2.57E+00
ARHGEF7	protein_coding	7.79E-05	1.16E-02	2.57E+00
ZNF333	protein_coding	8.34E-04	3.13E-02	2.57E+00
ZNF266	protein_coding	7.17E-04	2.97E-02	2.58E+00
KMT2A	protein_coding	6.22E-04	2.79E-02	2.59E+00
MDN1	protein_coding	8.18E-04	3.13E-02	2.60E+00
DDX60L	protein_coding	2.79E-04	1.95E-02	2.60E+00
RESF1	protein_coding	4.83E-04	2.46E-02	2.61E+00
CENPJ	protein_coding	9.48E-05	1.32E-02	2.62E+00
ATXN3	protein_coding	1.90E-03	4.60E-02	2.62E+00
REV3L	protein_coding	4.66E-04	2.42E-02	2.63E+00
LRP6	protein_coding	7.28E-04	2.97E-02	2.63E+00
POLR1A	protein_coding	1.72E-03	4.37E-02	2.64E+00
AFG3L1P	transcribed_unitary_pseudogene	2.75E-06	1.80E-03	2.65E+00
DIPK1B	protein_coding	2.15E-03	4.88E-02	2.65E+00
ITSN1	protein_coding	9.17E-05	1.29E-02	2.66E+00
TCHP	protein_coding	1.26E-03	3.85E-02	2.66E+00
MYO18A	protein_coding	1.94E-03	4.66E-02	2.66E+00
SHPRH	protein_coding	2.60E-05	6.93E-03	2.68E+00
ODF2L	protein_coding	9.29E-04	3.29E-02	2.69E+00
SGSM2	protein_coding	1.69E-04	1.64E-02	2.69E+00
EVL	protein_coding	1.56E-04	1.62E-02	2.70E+00
ZNF440	protein_coding	2.74E-04	1.95E-02	2.71E+00
SEPTIN7P2	transcribed_unprocessed_pseudogene	3.07E-04	1.98E-02	2.72E+00
CCDC14	protein_coding	5.68E-05	9.75E-03	2.72E+00
CCDC88A	protein_coding	4.15E-04	2.28E-02	2.73E+00
HERC2P3	transcribed_unprocessed_pseudogene	1.36E-03	3.99E-02	2.73E+00

ZNF26	protein_coding	2.16E-03	4.90E-02	2.75E+00
PRKD3	protein_coding	2.34E-04	1.80E-02	2.77E+00
UPF3A	protein_coding	1.53E-05	5.02E-03	2.78E+00
TBC1D4	protein_coding	7.22E-05	1.11E-02	2.78E+00
ALMS1	protein_coding	1.60E-03	4.23E-02	2.81E+00
ZNF337	protein_coding	1.77E-03	4.40E-02	2.81E+00
DDHD1	protein_coding	1.01E-03	3.41E-02	2.81E+00
SESTD1	protein_coding	7.83E-04	3.10E-02	2.82E+00
AHI1	protein_coding	1.61E-06	1.25E-03	2.83E+00
SH3BP5-AS1	lncRNA	8.92E-04	3.26E-02	2.84E+00
KSR1	protein_coding	1.69E-03	4.35E-02	2.85E+00
FAM228B	protein_coding	2.40E-04	1.82E-02	2.86E+00
ARAP3	protein_coding	1.59E-03	4.23E-02	2.87E+00
C3orf62	protein_coding	2.14E-03	4.88E-02	2.87E+00
CDK17	protein_coding	2.17E-06	1.54E-03	2.87E+00
FAM111A	protein_coding	1.52E-04	1.62E-02	2.90E+00
SYNE3	protein_coding	1.75E-03	4.38E-02	2.90E+00
ELP1	protein_coding	5.40E-04	2.60E-02	2.92E+00
LINC00472	lncRNA	1.11E-03	3.60E-02	2.94E+00
TANC1	protein_coding	1.85E-04	1.70E-02	2.95E+00
NKTR	protein_coding	4.11E-05	8.59E-03	2.95E+00
TTLL3	protein_coding	1.79E-04	1.70E-02	2.96E+00
SP110	protein_coding	6.17E-04	2.79E-02	2.97E+00
NPHP3	protein_coding	6.83E-09	5.83E-05	2.97E+00
NLRC5	protein_coding	1.14E-03	3.65E-02	2.99E+00
LRRC37A2	protein_coding	2.05E-04	1.79E-02	3.00E+00
GK5	protein_coding	1.17E-03	3.71E-02	3.00E+00
TTLL5	protein_coding	1.03E-03	3.44E-02	3.02E+00
SNX33	protein_coding	2.30E-04	1.80E-02	3.02E+00
PEAK1	protein_coding	6.39E-04	2.81E-02	3.03E+00
FAM241A	protein_coding	1.63E-03	4.26E-02	3.03E+00
STK36	protein_coding	1.44E-03	4.07E-02	3.04E+00
ARMCX4	protein_coding	2.18E-03	4.92E-02	3.04E+00
LAT	protein_coding	2.09E-03	4.80E-02	3.06E+00
DOCK5	protein_coding	6.49E-05	1.06E-02	3.07E+00
GASK1A	protein_coding	2.15E-04	1.80E-02	3.11E+00
ZNF577	protein_coding	2.54E-04	1.87E-02	3.11E+00
NFIA	protein_coding	2.08E-04	1.79E-02	3.13E+00
CRELD2	protein_coding	2.41E-04	1.82E-02	3.14E+00

PDK4-AS1	lncRNA	1.20E-03	3.76E-02	3.15E+00
CEP250	protein_coding	8.19E-04	3.13E-02	3.23E+00
FLYWCH1	protein_coding	8.96E-04	3.26E-02	3.23E+00
CDC42BPA	protein_coding	2.35E-04	1.80E-02	3.24E+00
PHTF1	protein_coding	3.58E-04	2.14E-02	3.25E+00
SLC1A2	protein_coding	3.66E-04	2.17E-02	3.26E+00
FGFR1	protein_coding	1.82E-04	1.70E-02	3.28E+00
CC2D2A	protein_coding	1.40E-03	4.04E-02	3.28E+00
TNFRSF25	protein_coding	7.71E-04	3.07E-02	3.28E+00
SRSF11	protein_coding	4.12E-05	8.59E-03	3.29E+00
GRB10	protein_coding	1.23E-04	1.45E-02	3.30E+00
CCDC144CP	transcribed_processed_pseudogene	1.70E-03	4.36E-02	3.32E+00
TTC28	protein_coding	3.84E-04	2.21E-02	3.32E+00
HPS4	protein_coding	1.20E-04	1.43E-02	3.32E+00
AP4B1	protein_coding	6.36E-04	2.81E-02	3.39E+00
ITPR1	protein_coding	5.31E-04	2.58E-02	3.40E+00
TAMALIN	protein_coding	2.03E-03	4.74E-02	3.41E+00
SFI1	protein_coding	1.18E-03	3.72E-02	3.41E+00
KAT2A	protein_coding	8.60E-07	9.18E-04	3.42E+00
CEP290	protein_coding	1.15E-05	4.48E-03	3.47E+00
MAPK8IP3	protein_coding	6.35E-04	2.81E-02	3.49E+00
TRIML2	protein_coding	2.29E-04	1.80E-02	3.50E+00
WDPCP	protein_coding	1.61E-03	4.24E-02	3.50E+00
AASS	protein_coding	4.73E-05	8.97E-03	3.50E+00
PLCE1	protein_coding	9.30E-04	3.29E-02	3.51E+00
TTC39B	protein_coding	6.18E-04	2.79E-02	3.51E+00
FADS3	protein_coding	2.26E-04	1.80E-02	3.51E+00
CEP120	protein_coding	3.98E-04	2.25E-02	3.54E+00
NRBP2	protein_coding	4.25E-04	2.31E-02	3.54E+00
GFOD1	protein_coding	2.28E-04	1.80E-02	3.56E+00
NOPCHAP1	protein_coding	1.58E-03	4.23E-02	3.57E+00
COPG2IT1	lncRNA	1.13E-03	3.62E-02	3.57E+00
PPFIBP2	protein_coding	2.83E-04	1.95E-02	3.58E+00
NR2F2-AS1	lncRNA	5.78E-04	2.71E-02	3.61E+00
SRGAP1	protein_coding	7.95E-05	1.16E-02	3.61E+00
PLCB4	protein_coding	6.04E-04	2.75E-02	3.61E+00
CCDC144B	transcribed_unprocessed_pseudogene	4.19E-06	2.24E-03	3.63E+00

AGAP6	protein_coding	9.56E-05	1.32E-02	3.64E+00
FLVCR1	protein_coding	5.35E-04	2.59E-02	3.64E+00
GOLGA8B	protein_coding	2.71E-05	7.01E-03	3.65E+00
SLX1B-SULT1A4	lncRNA	1.46E-03	4.09E-02	3.66E+00
HSF4	protein_coding	2.20E-03	4.93E-02	3.66E+00
FAM193A	protein_coding	1.07E-04	1.37E-02	3.67E+00
SYNE1	protein_coding	4.72E-04	2.43E-02	3.67E+00
PDCL3P4	transcribed_processed_pseudogene	1.38E-03	4.01E-02	3.67E+00
FHOD1	protein_coding	7.86E-05	1.16E-02	3.71E+00
ZNF704	protein_coding	2.78E-07	4.40E-04	3.71E+00
SOX6	protein_coding	1.12E-03	3.60E-02	3.73E+00
HMCN1	protein_coding	3.60E-04	2.14E-02	3.75E+00
ANKRD36B	protein_coding	1.50E-04	1.62E-02	3.75E+00
NPDC1	protein_coding	6.78E-04	2.90E-02	3.77E+00
LINC01415	lncRNA	1.71E-03	4.36E-02	3.79E+00
MIR193BHG	lncRNA	7.94E-04	3.10E-02	3.80E+00
ALS2CL	protein_coding	4.76E-06	2.47E-03	3.81E+00
DYNC2H1	protein_coding	8.24E-04	3.13E-02	3.82E+00
ZNF331	protein_coding	5.71E-05	9.75E-03	3.82E+00
ADAMTS18	protein_coding	2.04E-03	4.74E-02	3.83E+00
GOLGA6L9	protein_coding	3.12E-04	1.98E-02	3.83E+00
PPIEL	transcribed_unprocessed_pseudogene	3.35E-04	2.08E-02	3.83E+00
KLF7	protein_coding	2.28E-03	4.98E-02	3.85E+00
LINC01881	transcribed_unprocessed_pseudogene	8.30E-04	3.13E-02	3.89E+00
CACNA2D1	protein_coding	5.34E-04	2.59E-02	3.92E+00
SNHG7	lncRNA	4.95E-04	2.49E-02	3.93E+00
USP32P1	transcribed_unprocessed_pseudogene	1.57E-04	1.62E-02	3.94E+00
KIAA0895L	protein_coding	3.19E-05	7.26E-03	3.95E+00
WDR19	protein_coding	9.76E-05	1.33E-02	3.96E+00
TRDMT1	protein_coding	1.44E-03	4.07E-02	3.99E+00
SCAND2P	transcribed_unprocessed_pseudogene	1.32E-03	3.94E-02	4.00E+00
TAL1	protein_coding	3.71E-04	2.18E-02	4.01E+00
KANK3	protein_coding	1.56E-04	1.62E-02	4.02E+00
RTTN	protein_coding	6.68E-04	2.87E-02	4.05E+00

VEGFC	protein_coding	1.73E-03	4.37E-02	4.06E+00
TRPM4	protein_coding	3.75E-04	2.18E-02	4.06E+00
LUC7L3	protein_coding	1.10E-06	1.03E-03	4.06E+00
GUSBP11	lncRNA	5.83E-05	9.87E-03	4.07E+00
DONSON	protein_coding	2.91E-04	1.95E-02	4.08E+00
ZNF780B	protein_coding	9.99E-04	3.40E-02	4.09E+00
ADARB1	protein_coding	8.00E-04	3.10E-02	4.09E+00
LINC01725	lncRNA	2.20E-03	4.93E-02	4.11E+00
UPF3AP1	processed_pseudogene	1.95E-03	4.67E-02	4.11E+00
LLGL2	protein_coding	4.83E-04	2.46E-02	4.15E+00
TSEN2	protein_coding	1.39E-03	4.03E-02	4.19E+00
PPARGC1B	protein_coding	2.26E-03	4.98E-02	4.19E+00
NPIPA1	protein_coding	7.22E-05	1.11E-02	4.20E+00
NRXN3	protein_coding	6.50E-04	2.82E-02	4.22E+00
NCOR2	protein_coding	6.47E-04	2.82E-02	4.24E+00
RRAD	protein_coding	9.74E-04	3.36E-02	4.24E+00
SPINK5	protein_coding	1.11E-04	1.39E-02	4.25E+00
TRAF5	protein_coding	2.19E-04	1.80E-02	4.26E+00
TTN	protein_coding	6.03E-06	2.86E-03	4.27E+00
CCM2L	protein_coding	6.26E-04	2.79E-02	4.27E+00
PHLDB1	protein_coding	2.14E-03	4.87E-02	4.27E+00
MEG8	lncRNA	1.93E-04	1.75E-02	4.29E+00
SLC12A2-DT	lncRNA	1.00E-03	3.40E-02	4.31E+00
SLC25A25-AS1	lncRNA	1.36E-03	3.99E-02	4.34E+00
TONSL	protein_coding	1.15E-03	3.65E-02	4.34E+00
LINC01205	lncRNA	2.27E-03	4.98E-02	4.34E+00
AGAP9	protein_coding	1.24E-03	3.81E-02	4.39E+00
ANKRD36C	protein_coding	3.41E-04	2.08E-02	4.41E+00
ECMXP	transcribed_unitary_pseudogene	2.13E-03	4.86E-02	4.44E+00
CAPN3	protein_coding	1.07E-07	2.28E-04	4.44E+00
ASAP3	protein_coding	4.41E-04	2.36E-02	4.45E+00
PKD2L2-DT	lncRNA	1.98E-03	4.68E-02	4.51E+00
PKP4	protein_coding	2.12E-04	1.80E-02	4.52E+00
CELF6	protein_coding	1.81E-03	4.47E-02	4.53E+00
LRRFIP1P1	processed_pseudogene	1.73E-04	1.66E-02	4.53E+00
TTC21A	protein_coding	1.04E-04	1.37E-02	4.57E+00
POU6F1	protein_coding	1.45E-04	1.61E-02	4.58E+00

ZNF793	protein_coding	1.90E-03	4.60E-02	4.59E+00
HIVEP3	protein_coding	1.42E-03	4.06E-02	4.67E+00
RBM25	protein_coding	7.47E-06	3.27E-03	4.69E+00
ZNF782	protein_coding	4.94E-04	2.49E-02	4.71E+00
STX18-AS1	lncRNA	4.06E-04	2.27E-02	4.73E+00
JPH2	protein_coding	4.94E-04	2.49E-02	4.74E+00
MAGI2	protein_coding	5.55E-04	2.64E-02	4.76E+00
NLRP1	protein_coding	7.06E-04	2.95E-02	4.80E+00
PVT1	lncRNA	3.00E-04	1.95E-02	4.82E+00
ZNF334	protein_coding	9.78E-05	1.33E-02	4.83E+00
HSP90AB4P	processed_pseudogene	8.44E-04	3.14E-02	4.85E+00
DUXAP10	transcribed_processed_pseudogene	1.36E-03	3.99E-02	4.88E+00
FSTL3	protein_coding	8.20E-04	3.13E-02	4.95E+00
HULC	lncRNA	5.27E-04	2.58E-02	4.97E+00
DOCK6	protein_coding	7.89E-04	3.10E-02	4.97E+00
SYT15B	protein_coding	8.01E-04	3.10E-02	4.97E+00
COLQ	protein_coding	1.86E-03	4.53E-02	5.00E+00
COL27A1	protein_coding	6.23E-07	7.10E-04	5.01E+00
ZMAT1	protein_coding	1.45E-05	4.96E-03	5.03E+00
CNKSR3	protein_coding	7.87E-04	3.10E-02	5.08E+00
ANKRD36	protein_coding	1.20E-04	1.43E-02	5.08E+00
RASGRF2	protein_coding	2.79E-05	7.01E-03	5.08E+00
LINC01515	lncRNA	2.27E-03	4.98E-02	5.09E+00
ANKRD20A17P	unprocessed_pseudogene	2.85E-04	1.95E-02	5.09E+00
PROSER3	protein_coding	5.51E-04	2.63E-02	5.09E+00
MEG3	lncRNA	1.47E-04	1.62E-02	5.12E+00
ARHGEF10	protein_coding	4.01E-04	2.26E-02	5.18E+00
LRRC8B	protein_coding	1.07E-04	1.37E-02	5.23E+00
ADAMTS15	protein_coding	5.84E-04	2.73E-02	5.25E+00
ZNF483	protein_coding	1.11E-03	3.60E-02	5.25E+00
TCAM1P	transcribed_unitary_pseudogene	8.92E-04	3.26E-02	5.28E+00
PTCHD4	protein_coding	4.43E-04	2.36E-02	5.30E+00
TMEM44	protein_coding	5.13E-04	2.54E-02	5.32E+00
DYNC2I1	protein_coding	2.38E-07	4.40E-04	5.37E+00
NSMF	protein_coding	9.87E-04	3.37E-02	5.41E+00
POU5F2	protein_coding	3.92E-04	2.23E-02	5.42E+00

SYCP2	protein_coding	4.31E-07	5.26E-04	5.42E+00
DNAH2	protein_coding	1.60E-03	4.23E-02	5.43E+00
MUC16	protein_coding	1.50E-03	4.10E-02	5.43E+00
ZNF699	protein_coding	1.58E-03	4.23E-02	5.43E+00
GPRC5A	protein_coding	1.47E-03	4.09E-02	5.47E+00
ZNF37BP	transcribed_processed_pseudogene	3.11E-07	4.41E-04	5.47E+00
ZNF66	protein_coding	4.83E-04	2.46E-02	5.48E+00
ARL10	protein_coding	5.65E-04	2.67E-02	5.54E+00
COL18A1	protein_coding	5.92E-04	2.73E-02	5.59E+00
CEP164	protein_coding	2.52E-05	6.84E-03	5.60E+00
NPIPB11	protein_coding	8.29E-04	3.13E-02	5.61E+00
PTOV1-AS1	lncRNA	7.43E-04	3.02E-02	5.63E+00
ATP8A2	protein_coding	7.10E-04	2.96E-02	5.65E+00
XACT	lncRNA	2.04E-03	4.74E-02	5.72E+00
STOX2	protein_coding	1.66E-05	5.27E-03	5.74E+00
NOTCH4	protein_coding	1.05E-04	1.37E-02	5.74E+00
WHAMMP1	transcribed_processed_pseudogene	9.65E-04	3.35E-02	5.78E+00
SAP30L-AS1	lncRNA	2.26E-03	4.98E-02	5.84E+00
ZNF284	protein_coding	2.09E-03	4.80E-02	5.85E+00
PPP5D1P	transcribed_unprocessed_pseudogene	7.01E-04	2.94E-02	5.87E+00
SOBP	protein_coding	6.02E-04	2.75E-02	5.88E+00
RIPOR3	protein_coding	1.87E-03	4.55E-02	5.89E+00
SMARCD3	protein_coding	1.72E-03	4.37E-02	5.91E+00
FN1	protein_coding	1.60E-03	4.23E-02	5.94E+00
SATB2	protein_coding	1.07E-03	3.53E-02	5.96E+00
KCTD15	protein_coding	6.59E-05	1.06E-02	5.96E+00
HOXB3	protein_coding	2.91E-04	1.95E-02	5.97E+00
ZFAND2A-DT	lncRNA	4.69E-04	2.42E-02	5.98E+00
MTND5P11	processed_pseudogene	2.22E-04	1.80E-02	5.99E+00
GOLGA6L4	protein_coding	6.66E-04	2.87E-02	6.03E+00
HERC2P4	transcribed_unprocessed_pseudogene	9.07E-04	3.27E-02	6.03E+00
CDRT4	protein_coding	7.32E-06	3.27E-03	6.04E+00
CHDH	protein_coding	5.22E-04	2.57E-02	6.06E+00
LINC00506	lncRNA	1.39E-04	1.58E-02	6.16E+00
CROCCP3	transcribed_unprocessed_pseudogene	2.99E-04	1.95E-02	6.16E+00

ZNF660	protein_coding	1.08E-03	3.55E-02	6.17E+00
SLC9A3-AS1	lncRNA	6.11E-04	2.77E-02	6.22E+00
HELLPAR	lncRNA	3.56E-05	8.00E-03	6.27E+00
APRG1	lncRNA	9.04E-04	3.27E-02	6.34E+00
SNHG14	lncRNA	9.06E-06	3.77E-03	6.36E+00
GABBR1	protein_coding	7.90E-05	1.16E-02	6.43E+00
CNTNAP1	protein_coding	2.00E-03	4.71E-02	6.47E+00
KCNQ1OT1	lncRNA	6.29E-08	1.79E-04	6.47E+00
CCDC30	protein_coding	7.75E-05	1.16E-02	6.48E+00
CNTNAP3	protein_coding	3.92E-04	2.23E-02	6.52E+00
MTMR9LP	transcribed_unprocessed_pseudogene	5.28E-04	2.58E-02	6.53E+00
ANXA2R-AS1	lncRNA	3.04E-05	7.21E-03	6.56E+00
TRPV3	protein_coding	1.07E-03	3.53E-02	6.58E+00
BDH1	protein_coding	2.97E-04	1.95E-02	6.63E+00
KIAA1671	protein_coding	1.39E-03	4.03E-02	6.66E+00
AKAP6	protein_coding	1.36E-03	3.99E-02	6.66E+00
NFASC	protein_coding	1.58E-04	1.62E-02	6.66E+00
BCO2	protein_coding	3.14E-04	1.98E-02	6.67E+00
GOLGA8A	protein_coding	1.18E-08	6.71E-05	6.74E+00
QRICH2	protein_coding	2.20E-03	4.93E-02	6.75E+00
GRIK2	protein_coding	2.32E-04	1.80E-02	6.76E+00
ABCC9	protein_coding	3.23E-04	2.02E-02	6.77E+00
LINC02895	lncRNA	2.18E-04	1.80E-02	6.81E+00
DCST2	protein_coding	1.04E-03	3.45E-02	6.81E+00
CCDC144A	protein_coding	5.48E-06	2.68E-03	6.90E+00
CRYGS	protein_coding	8.64E-04	3.20E-02	7.01E+00
BVES	protein_coding	8.36E-04	3.13E-02	7.04E+00
LINC02969	lncRNA	2.20E-04	1.80E-02	7.05E+00
BMS1P1	transcribed_unprocessed_pseudogene	2.47E-04	1.84E-02	7.08E+00
POTEF	protein_coding	1.22E-03	3.79E-02	7.13E+00
BMP8A	protein_coding	1.73E-03	4.37E-02	7.14E+00
GINS4	protein_coding	1.49E-03	4.10E-02	7.16E+00
CCDC168	protein_coding	2.03E-04	1.79E-02	7.18E+00
TMED2-DT	lncRNA	2.14E-04	1.80E-02	7.20E+00
CYP2E1	protein_coding	1.99E-04	1.77E-02	7.25E+00
SPRY3	protein_coding	1.81E-04	1.70E-02	7.25E+00
CASC19	lncRNA	4.14E-04	2.28E-02	7.26E+00

EGFR-AS1	lncRNA	3.12E-04	1.98E-02	7.31E+00
IDO2	protein_coding	1.03E-05	4.18E-03	7.39E+00
COL5A3	protein_coding	1.50E-03	4.10E-02	7.48E+00
FRG1-DT	lncRNA	1.50E-03	4.10E-02	7.49E+00
PKD1L2	protein_coding	5.61E-05	9.75E-03	7.54E+00
CHRNA10	protein_coding	2.26E-03	4.98E-02	7.54E+00
KRT18P61	processed_pseudogene	3.17E-05	7.26E-03	7.55E+00
MYO15B	protein_coding	4.47E-05	8.78E-03	7.62E+00
NPAS3	protein_coding	1.43E-03	4.07E-02	7.63E+00
CACNB4	protein_coding	2.79E-05	7.01E-03	7.64E+00
EPM2A	protein_coding	4.56E-05	8.78E-03	7.69E+00
MARK4	protein_coding	1.24E-03	3.81E-02	7.75E+00
AGBL4	protein_coding	2.30E-03	5.00E-02	7.77E+00
KC6	lncRNA	1.83E-03	4.49E-02	7.78E+00
LTK	protein_coding	1.21E-06	1.03E-03	7.85E+00
MTCO3P12	unprocessed_pseudogene	7.88E-06	3.36E-03	7.97E+00
ADAMTS9-AS2	lncRNA	1.41E-05	4.93E-03	7.98E+00
LINC02028	lncRNA	6.61E-04	2.86E-02	7.98E+00
NMNAT3	protein_coding	6.87E-04	2.91E-02	7.99E+00
SPATA33	protein_coding	5.08E-04	2.53E-02	8.00E+00
PIPOX	protein_coding	8.96E-04	3.26E-02	8.20E+00
MCF2L	protein_coding	9.34E-04	3.29E-02	8.37E+00
ZNF337-AS1	lncRNA	1.02E-03	3.43E-02	8.37E+00
NSRP1P1	processed_pseudogene	1.73E-06	1.28E-03	8.52E+00
FAR2P1	transcribed_unprocessed_pseudogene	5.42E-04	2.61E-02	8.58E+00
BMP8B	protein_coding	7.21E-04	2.97E-02	8.60E+00
CTBP2P8	processed_pseudogene	2.89E-04	1.95E-02	8.65E+00
TTTY10	lncRNA	1.46E-03	4.09E-02	8.74E+00
AR	protein_coding	4.31E-04	2.32E-02	8.95E+00
MTCO2P12	unprocessed_pseudogene	1.49E-05	4.99E-03	8.95E+00
HABP4	protein_coding	1.18E-04	1.43E-02	9.09E+00
SLC4A8	protein_coding	2.15E-04	1.80E-02	9.12E+00
KCNMA1	protein_coding	2.08E-03	4.80E-02	9.14E+00
RNASEH2B-AS1	lncRNA	2.17E-03	4.90E-02	9.19E+00
FLJ43315	transcribed_unprocessed_pseudogene	1.08E-04	1.37E-02	9.29E+00
KIF5C	protein_coding	1.91E-03	4.60E-02	9.31E+00

SOX2-OT	lncRNA	2.04E-03	4.74E-02	9.34E+00
SLC51A	protein_coding	8.65E-04	3.20E-02	9.44E+00
MTCO2P2	processed_pseudogene	7.43E-06	3.27E-03	9.45E+00
NBEA	protein_coding	7.03E-04	2.95E-02	9.47E+00
SHANK3	protein_coding	5.98E-04	2.74E-02	9.47E+00
TMEM217	protein_coding	1.63E-03	4.26E-02	9.60E+00
SLC2A11	protein_coding	3.50E-06	2.03E-03	9.76E+00
POTEM	protein_coding	4.25E-04	2.31E-02	9.79E+00
OR8B1P	transcribed_unprocessed_pseudogene	1.16E-04	1.42E-02	9.91E+00
USP6	protein_coding	1.08E-04	1.37E-02	1.00E+01
MTND2P28	unprocessed_pseudogene	4.87E-05	9.15E-03	1.00E+01
HSPA6	protein_coding	2.97E-04	1.95E-02	1.02E+01
CACNA1C	protein_coding	3.69E-06	2.03E-03	1.02E+01
CYP46A1	protein_coding	1.49E-03	4.10E-02	1.02E+01
LINC00607	lncRNA	4.57E-05	8.78E-03	1.03E+01
CACNA1A	protein_coding	2.24E-03	4.96E-02	1.03E+01
SKA1	protein_coding	1.69E-03	4.35E-02	1.05E+01
GOLGA8R	protein_coding	1.94E-03	4.65E-02	1.06E+01
LETR1	lncRNA	1.92E-03	4.62E-02	1.08E+01
LINC00342	lncRNA	8.86E-08	2.16E-04	1.08E+01
LRRIQ1	protein_coding	1.25E-03	3.82E-02	1.08E+01
TNNI2	protein_coding	1.77E-03	4.40E-02	1.08E+01
CFAP69	protein_coding	2.08E-04	1.79E-02	1.10E+01
UBQLNL	protein_coding	1.29E-03	3.91E-02	1.10E+01
KLHL33	protein_coding	2.03E-03	4.74E-02	1.11E+01
ZC3H12D	protein_coding	1.60E-03	4.23E-02	1.11E+01
LINC00824	lncRNA	1.44E-03	4.07E-02	1.14E+01
RIMS2	protein_coding	1.99E-03	4.70E-02	1.14E+01
VEGFA	protein_coding	2.48E-05	6.83E-03	1.16E+01
NEXN-AS1	lncRNA	1.05E-03	3.49E-02	1.17E+01
LINC00504	lncRNA	1.00E-03	3.40E-02	1.17E+01
ADAM28	protein_coding	2.14E-05	6.20E-03	1.19E+01
ASCL2	protein_coding	7.90E-04	3.10E-02	1.20E+01
FAM27C	lncRNA	4.58E-04	2.40E-02	1.24E+01
GLYATL1	protein_coding	2.26E-03	4.98E-02	1.29E+01
ADGRF1	protein_coding	3.15E-04	1.98E-02	1.29E+01
TPH1	protein_coding	7.58E-04	3.06E-02	1.31E+01
ZFP57	protein_coding	1.68E-03	4.32E-02	1.33E+01

TDRD1	protein_coding	2.92E-04	1.95E-02	1.33E+01
FBXW10B	protein_coding	1.72E-05	5.35E-03	1.34E+01
CPB2-AS1	lncRNA	5.88E-04	2.73E-02	1.34E+01
TRDN-AS1	lncRNA	1.49E-03	4.10E-02	1.36E+01
KIF5A	protein_coding	1.95E-03	4.67E-02	1.36E+01
ZNF774	protein_coding	1.52E-04	1.62E-02	1.37E+01
ST8SIA1	protein_coding	8.16E-04	3.13E-02	1.38E+01
LINC02984	lncRNA	2.28E-03	4.98E-02	1.39E+01
DLEC1	protein_coding	9.33E-04	3.29E-02	1.40E+01
POU5F1B	protein_coding	4.07E-04	2.27E-02	1.40E+01
LINC01506	lncRNA	6.39E-04	2.81E-02	1.41E+01
FGB	protein_coding	1.32E-03	3.94E-02	1.45E+01
TRIM64B	protein_coding	2.09E-03	4.80E-02	1.45E+01
ZACN	protein_coding	1.66E-03	4.30E-02	1.45E+01
XKR4	protein_coding	1.94E-04	1.75E-02	1.46E+01
LINC00499	lncRNA	7.01E-04	2.94E-02	1.49E+01
L3MBTL4	protein_coding	7.86E-04	3.10E-02	1.52E+01
KRT8P32	processed_pseudogene	8.80E-04	3.24E-02	1.54E+01
MTATP6P1	unprocessed_pseudogene	5.75E-08	1.79E-04	1.54E+01
SPAG8	protein_coding	1.87E-05	5.70E-03	1.55E+01
PCDHB9	protein_coding	9.71E-04	3.36E-02	1.57E+01
MTND4P12	processed_pseudogene	2.14E-09	3.66E-05	1.57E+01
SCOC-AS1	lncRNA	1.02E-03	3.43E-02	1.59E+01
KRT18P22	processed_pseudogene	1.20E-03	3.76E-02	1.59E+01
JAKMIP2-AS1	lncRNA	1.47E-03	4.09E-02	1.61E+01
LINC02346	lncRNA	1.51E-03	4.12E-02	1.62E+01
PCDHGB8P	transcribed_unitary_pseudogene	2.15E-03	4.88E-02	1.63E+01
LINC01570	lncRNA	2.12E-03	4.85E-02	1.63E+01
KRT16P6	transcribed_unprocessed_pseudogene	2.86E-05	7.09E-03	1.64E+01
STK32A	protein_coding	1.84E-03	4.49E-02	1.65E+01
ANKRD18A	protein_coding	2.27E-05	6.37E-03	1.67E+01
CD109-AS1	lncRNA	8.71E-05	1.25E-02	1.70E+01
PRAL	TEC	4.03E-05	8.59E-03	1.71E+01
TNR	protein_coding	3.83E-05	8.38E-03	1.71E+01
PSMD7-DT	lncRNA	1.75E-03	4.38E-02	1.73E+01
ARHGEF4	protein_coding	2.05E-04	1.79E-02	1.74E+01
KRT18P35	processed_pseudogene	2.98E-04	1.95E-02	1.79E+01

IGSF9B	protein_coding	1.58E-04	1.62E-02	1.80E+01
XRCC6P5	processed_pseudogene	1.76E-03	4.38E-02	1.81E+01
LRRC37A4P	transcribed_unprocessed_pseudogene	1.61E-03	4.23E-02	1.83E+01
RET	protein_coding	1.90E-03	4.60E-02	1.85E+01
OR10A3	protein_coding	3.85E-04	2.21E-02	1.85E+01
LINC00951	lncRNA	1.13E-03	3.62E-02	1.90E+01
OR51A3P	unprocessed_pseudogene	1.70E-03	4.36E-02	1.94E+01
PGM5P2	transcribed_unprocessed_pseudogene	1.27E-04	1.48E-02	1.95E+01
SV2C	protein_coding	3.39E-04	2.08E-02	1.96E+01
ZRANB2-DT	lncRNA	6.24E-05	1.05E-02	1.96E+01
LINC02023	lncRNA	1.72E-03	4.37E-02	1.97E+01
EGLN3	protein_coding	2.28E-05	6.37E-03	1.99E+01
LINC00461	lncRNA	1.15E-03	3.67E-02	2.00E+01
FAM133CP	processed_pseudogene	6.44E-04	2.81E-02	2.02E+01
LINC00598	lncRNA	2.41E-04	1.82E-02	2.03E+01
BCL2L2-PABPN1	protein_coding	1.01E-04	1.36E-02	2.04E+01
RN7SL37P	misc_RNA	7.27E-04	2.97E-02	2.04E+01
NPAP1	protein_coding	1.31E-03	3.93E-02	2.05E+01
DAAM2-AS1	lncRNA	2.17E-04	1.80E-02	2.05E+01
FHOD3	protein_coding	1.48E-03	4.10E-02	2.08E+01
GRIN2A	protein_coding	1.97E-04	1.76E-02	2.11E+01
LINC01697	lncRNA	1.30E-04	1.51E-02	2.13E+01
PADI1	protein_coding	5.79E-04	2.71E-02	2.14E+01
AADACL3	protein_coding	2.50E-04	1.85E-02	2.19E+01
AKR7L	protein_coding	8.42E-04	3.14E-02	2.20E+01
CDYLP1	unprocessed_pseudogene	2.34E-04	1.80E-02	2.25E+01
LINC02008	lncRNA	1.45E-03	4.09E-02	2.27E+01
GRIN2B	protein_coding	1.66E-04	1.64E-02	2.32E+01
TET1P1	processed_pseudogene	1.53E-03	4.15E-02	2.43E+01
LINC00895	lncRNA	1.27E-03	3.87E-02	2.45E+01
HSPA7	unprocessed_pseudogene	5.98E-08	1.79E-04	2.46E+01
LINC01471	lncRNA	1.55E-03	4.18E-02	2.53E+01
RHPN2P1	unprocessed_pseudogene	6.43E-04	2.81E-02	2.54E+01
TCP10L	protein_coding	6.48E-05	1.06E-02	2.56E+01
LRRC38	protein_coding	6.72E-05	1.06E-02	2.58E+01
OR10G3	protein_coding	1.03E-03	3.43E-02	2.68E+01

STEAP3-AS1	lncRNA	2.17E-03	4.91E-02	2.70E+01
MTND4LP30	processed_pseudogene	9.62E-05	1.32E-02	2.73E+01
DNM1P46	transcribed_unprocessed_pseudogene	1.10E-03	3.59E-02	2.78E+01
LINC02664	lncRNA	3.69E-04	2.18E-02	2.78E+01
CNNM1	protein_coding	5.09E-04	2.53E-02	2.84E+01
NTRK3	protein_coding	5.30E-05	9.68E-03	2.89E+01
CLDN18	protein_coding	2.02E-03	4.73E-02	2.91E+01
LINC01127	lncRNA	8.76E-04	3.23E-02	2.93E+01
OR4K13	protein_coding	4.34E-04	2.32E-02	3.46E+01
OR51M1	protein_coding	8.87E-04	3.25E-02	3.52E+01
HMGA2-AS1	lncRNA	1.66E-03	4.30E-02	3.69E+01
EMSLR	lncRNA	1.67E-03	4.31E-02	3.78E+01
RNF212	protein_coding	2.96E-05	7.19E-03	3.98E+01
LINC02465	lncRNA	1.44E-04	1.61E-02	4.94E+01
OR5AS1	protein_coding	3.18E-06	1.94E-03	7.36E+01
