# Supplementary material for: Global Transcriptomic Analysis of Placentas from Women with Gestational SARS-CoV-2 Infection during the Third Trimester of Pregnancy
Source: Int J Mol Sci. 2024 Jan 28;25(3):1608. doi: 10.3390/ijms25031608 (PMC10855544; doi:10.3390/ijms25031608)
Supplement: Supplementary file 1 [file ijms-25-01608-s001.zip › Supplementary Table S2- symptomatic vs healthy.pdf]

Supplementary table

Differentially Expressed Genes in the maternal placental compartment among women with COVID-19 symptoms, compared to healthy controls. Analysis utilized stringent DEG criteria with FDR of 0.01. Genes are presented in ascending order of fold change

Gene name	Gene biotype	P-value	FDR step up	Fold change
PAPPA-AS1	lncRNA	1.17E-05	4.38E-04	-1.44E+02
CGB5	protein_coding	2.57E-04	3.78E-03	-5.37E+01
EBI3	protein_coding	3.78E-09	7.93E-07	-4.98E+01
CGB8	protein_coding	4.67E-04	5.67E-03	-3.85E+01
TFPI2	protein_coding	9.43E-10	2.72E-07	-3.56E+01
CRH	protein_coding	1.52E-07	1.51E-05	-3.42E+01
GDF15	protein_coding	2.26E-09	5.33E-07	-3.33E+01
KISS1	protein_coding	5.79E-04	6.50E-03	-3.27E+01
PSG9	protein_coding	1.31E-08	2.11E-06	-3.10E+01
PSG1	protein_coding	4.28E-10	1.47E-07	-2.84E+01
ALPP	protein_coding	1.86E-07	1.80E-05	-2.79E+01
PSG3	protein_coding	9.57E-09	1.62E-06	-2.76E+01
CGA	protein_coding	2.15E-09	5.13E-07	-2.56E+01
SCNN1B	protein_coding	3.04E-04	4.22E-03	-2.56E+01
ENDOU	protein_coding	1.86E-07	1.80E-05	-2.05E+01
PSG6	protein_coding	8.42E-09	1.46E-06	-2.00E+01
INSL4	protein_coding	1.67E-08	2.60E-06	-1.87E+01
CA10	protein_coding	2.23E-04	3.42E-03	-1.81E+01
PAPPA	protein_coding	4.40E-07	3.65E-05	-1.80E+01
PSG11	protein_coding	1.53E-05	5.30E-04	-1.78E+01
SDC1	protein_coding	3.38E-06	1.77E-04	-1.76E+01
PSG4	protein_coding	1.01E-08	1.69E-06	-1.71E+01
PSG5	protein_coding	2.15E-07	2.03E-05	-1.68E+01
PSG2	protein_coding	4.02E-09	8.34E-07	-1.68E+01
CYP19A1	protein_coding	5.47E-08	6.77E-06	-1.66E+01
ADAM12	protein_coding	9.29E-07	6.53E-05	-1.56E+01
PLAC1	protein_coding	1.93E-07	1.86E-05	-1.52E+01
PSG10P	transcribed_unprocessed_pseudogene	7.70E-06	3.23E-04	-1.48E+01
PSG8	protein_coding	8.03E-08	9.17E-06	-1.45E+01
LAPTM4A	protein_coding	8.74E-14	1.51E-10	-1.45E+01
GH1	protein_coding	5.47E-07	4.38E-05	-1.43E+01
CXCL1	protein_coding	3.41E-05	9.19E-04	-1.41E+01

HSPB1	protein_coding	1.44E-07	1.46E-05	-1.35E+01
GPC3	protein_coding	4.74E-09	9.23E-07	-1.33E+01
LYPD3	protein_coding	4.86E-06	2.33E-04	-1.26E+01
SDSL	protein_coding	2.14E-04	3.34E-03	-1.20E+01
PAGE4	protein_coding	2.13E-08	3.16E-06	-1.17E+01
FBLN1	protein_coding	3.47E-06	1.80E-04	-1.16E+01
CSH2	protein_coding	6.42E-06	2.83E-04	-1.16E+01
CD14	protein_coding	6.36E-07	4.93E-05	-1.11E+01
CD74	protein_coding	6.16E-04	6.79E-03	-1.11E+01
BGN	protein_coding	3.72E-05	9.76E-04	-1.10E+01
SPATA2L	protein_coding	5.70E-04	6.43E-03	-1.09E+01
CLDN7	protein_coding	2.10E-08	3.13E-06	-1.09E+01
CLDN1	protein_coding	1.42E-05	5.05E-04	-1.07E+01
GRN	protein_coding	7.69E-13	8.15E-10	-1.07E+01
FKBP2	protein_coding	3.46E-06	1.80E-04	-1.03E+01
MIF-AS1	lncRNA	4.27E-04	5.37E-03	-1.02E+01
CPVL	protein_coding	6.08E-04	6.72E-03	-1.01E+01
VSIR	protein_coding	3.56E-12	2.51E-09	-9.97E+00
CSH1	protein_coding	1.36E-05	4.91E-04	-9.78E+00
CTSD	protein_coding	1.54E-13	2.45E-10	-9.72E+00
SEMA3B	protein_coding	1.78E-05	5.89E-04	-9.66E+00
TEN1	protein_coding	2.31E-05	6.97E-04	-9.44E+00
FURIN	protein_coding	8.91E-06	3.55E-04	-8.84E+00
GH2	protein_coding	3.36E-05	9.12E-04	-8.77E+00
SLC7A2	protein_coding	6.58E-05	1.45E-03	-8.57E+00
UBB	protein_coding	1.98E-04	3.16E-03	-8.48E+00
IFI30	protein_coding	5.74E-07	4.52E-05	-8.44E+00
CLDN4	protein_coding	6.23E-10	2.01E-07	-8.19E+00
CD274	protein_coding	3.19E-04	4.35E-03	-8.16E+00
RHOD	protein_coding	3.80E-04	4.96E-03	-8.12E+00
MFSD2A	protein_coding	1.00E-04	1.99E-03	-8.05E+00
ABHD12	protein_coding	4.38E-04	5.45E-03	-7.99E+00
TREML2	protein_coding	8.25E-04	8.22E-03	-7.98E+00
S100P	protein_coding	2.34E-06	1.35E-04	-7.95E+00
TNFRSF12A	protein_coding	1.11E-05	4.22E-04	-7.91E+00
BSG	protein_coding	4.45E-13	5.00E-10	-7.89E+00
SPINT2	protein_coding	1.80E-10	7.32E-08	-7.83E+00
TUBA1C	protein_coding	5.83E-05	1.35E-03	-7.81E+00
HLA-C	protein_coding	1.07E-03	9.89E-03	-7.80E+00

SERPINB2	protein_coding	1.16E-05	4.35E-04	-7.78E+00
FAM171B	protein_coding	2.28E-04	3.48E-03	-7.71E+00
BCAS4	protein_coding	5.37E-04	6.19E-03	-7.60E+00
CRIP1	protein_coding	1.12E-05	4.22E-04	-7.45E+00
TECRP1	processed_pseudogene	1.89E-05	6.09E-04	-7.44E+00
NECTIN3	protein_coding	3.96E-04	5.10E-03	-7.40E+00
CLIC3	protein_coding	2.92E-04	4.11E-03	-7.39E+00
XAGE3	protein_coding	1.34E-11	7.52E-09	-7.38E+00
SLC31A2	protein_coding	1.66E-04	2.81E-03	-7.23E+00
ERV3-1	protein_coding	1.08E-03	9.94E-03	-7.17E+00
FRZB	protein_coding	8.50E-09	1.46E-06	-7.09E+00
LGMN	protein_coding	3.76E-12	2.56E-09	-7.09E+00
SVEP1	protein_coding	1.23E-04	2.29E-03	-6.88E+00
MYDGF	protein_coding	5.50E-09	1.05E-06	-6.73E+00
MMP11	protein_coding	7.14E-04	7.53E-03	-6.64E+00
TUSC3	protein_coding	1.57E-05	5.42E-04	-6.63E+00
PDIA3P1	transcribed_processed_pseudogene	7.71E-08	8.95E-06	-6.62E+00
MAD1L1	protein_coding	3.89E-04	5.05E-03	-6.61E+00
FUCA1	protein_coding	2.40E-06	1.37E-04	-6.59E+00
COX6A1P2	processed_pseudogene	1.14E-06	7.63E-05	-6.50E+00
ESAM	protein_coding	1.84E-08	2.77E-06	-6.47E+00
CHID1	protein_coding	1.29E-11	7.47E-09	-6.43E+00
LNPEP	protein_coding	2.92E-04	4.11E-03	-6.41E+00
PSAP	protein_coding	1.86E-12	1.48E-09	-6.36E+00
PDIA6	protein_coding	2.13E-13	3.13E-10	-6.36E+00
DEGS1	protein_coding	8.27E-06	3.39E-04	-6.29E+00
MAGEA8	protein_coding	1.19E-05	4.44E-04	-6.28E+00
PGF	protein_coding	1.56E-04	2.69E-03	-6.27E+00
SLC39A8	protein_coding	1.80E-04	2.99E-03	-6.24E+00
TAF1B	protein_coding	1.16E-04	2.21E-03	-6.24E+00
RPN1	protein_coding	9.05E-13	9.08E-10	-6.21E+00
PGAP3	protein_coding	2.86E-04	4.05E-03	-6.13E+00
MGAT1	protein_coding	1.07E-07	1.15E-05	-6.10E+00
PCDH1	protein_coding	5.90E-04	6.58E-03	-6.09E+00
MS4A4A	protein_coding	2.89E-06	1.58E-04	-6.07E+00
ADIRF	protein_coding	4.66E-05	1.14E-03	-6.07E+00

CD320	protein_coding	8.74E-06	3.50E-04	-6.06E+00
TGM2	protein_coding	7.99E-11	3.63E-08	-5.98E+00
MIF	protein_coding	1.42E-09	3.69E-07	-5.92E+00
CTSL	protein_coding	2.29E-04	3.48E-03	-5.92E+00
MAN1C1	protein_coding	3.51E-04	4.68E-03	-5.90E+00
EEFSEC	protein_coding	1.73E-04	2.90E-03	-5.89E+00
CD63	protein_coding	8.11E-09	1.42E-06	-5.80E+00
HYOU1	protein_coding	1.06E-04	2.07E-03	-5.79E+00
FBN2	protein_coding	4.10E-06	2.05E-04	-5.76E+00
GBA	protein_coding	2.58E-06	1.45E-04	-5.68E+00
TIMP2	protein_coding	8.70E-04	8.53E-03	-5.64E+00
HSPA5	protein_coding	8.45E-12	5.37E-09	-5.64E+00
PPT1	protein_coding	5.68E-06	2.60E-04	-5.63E+00
RELL1	protein_coding	1.72E-04	2.89E-03	-5.61E+00
MRPL54	protein_coding	3.75E-06	1.92E-04	-5.61E+00
CDYL2	protein_coding	7.51E-04	7.76E-03	-5.59E+00
SLC3A2	protein_coding	4.91E-08	6.33E-06	-5.58E+00
VGLL3	protein_coding	2.02E-04	3.20E-03	-5.52E+00
AP1M2	protein_coding	5.96E-05	1.36E-03	-5.51E+00
HS3ST3B1	protein_coding	8.08E-04	8.13E-03	-5.51E+00
PPIB	protein_coding	1.08E-07	1.15E-05	-5.50E+00
GAA	protein_coding	5.20E-09	1.00E-06	-5.50E+00
POGLUT3	protein_coding	6.24E-04	6.83E-03	-5.47E+00
ATP1A1	protein_coding	2.18E-05	6.75E-04	-5.47E+00
DNAJB9	protein_coding	6.28E-08	7.63E-06	-5.47E+00
PLAU	protein_coding	1.28E-09	3.40E-07	-5.46E+00
PMVK	protein_coding	1.39E-04	2.47E-03	-5.45E+00
FXYD3	protein_coding	1.42E-04	2.50E-03	-5.42E+00
CSF2RB	protein_coding	7.00E-04	7.45E-03	-5.42E+00
MFAP2	protein_coding	6.53E-05	1.44E-03	-5.42E+00
EFEMP1	protein_coding	4.34E-04	5.42E-03	-5.41E+00
NOMO2	protein_coding	2.51E-04	3.72E-03	-5.40E+00
SSR4	protein_coding	8.28E-07	6.00E-05	-5.34E+00
SIL1	protein_coding	1.57E-06	9.91E-05	-5.31E+00
CD55	protein_coding	2.36E-08	3.40E-06	-5.30E+00
GPNMB	protein_coding	3.48E-05	9.27E-04	-5.29E+00
RPN2	protein_coding	2.64E-13	3.52E-10	-5.28E+00
GJA5	protein_coding	1.46E-10	6.06E-08	-5.22E+00
SLC39A7	protein_coding	2.52E-12	1.85E-09	-5.22E+00

SPINT1	protein_coding	6.98E-06	3.00E-04	-5.16E+00
UBL7	protein_coding	4.34E-06	2.13E-04	-5.14E+00
TMED9	protein_coding	1.19E-12	1.08E-09	-5.12E+00
TENT5A	protein_coding	1.57E-04	2.71E-03	-5.11E+00
SLC38A1	protein_coding	1.42E-05	5.04E-04	-5.11E+00
MUL1	protein_coding	2.03E-05	6.40E-04	-5.10E+00
CILK1	protein_coding	1.75E-04	2.91E-03	-5.05E+00
HSPB8	protein_coding	1.92E-06	1.16E-04	-5.04E+00
PRSS8	protein_coding	6.36E-04	6.93E-03	-5.03E+00
EFHD1	protein_coding	9.78E-05	1.96E-03	-5.01E+00
TEX264	protein_coding	2.28E-10	8.83E-08	-5.00E+00
RABAC1	protein_coding	1.42E-07	1.46E-05	-5.00E+00
PHYHIPL	protein_coding	3.63E-05	9.58E-04	-4.96E+00
COX6C	protein_coding	5.92E-09	1.11E-06	-4.96E+00
S100A11	protein_coding	7.86E-07	5.79E-05	-4.96E+00
SVBP	protein_coding	4.73E-04	5.72E-03	-4.95E+00
POR	protein_coding	2.16E-04	3.36E-03	-4.95E+00
TM9SF2	protein_coding	7.22E-10	2.21E-07	-4.94E+00
DHCR24	protein_coding	1.46E-08	2.32E-06	-4.92E+00
TMED10	protein_coding	2.82E-07	2.55E-05	-4.92E+00
PERP	protein_coding	2.09E-09	5.05E-07	-4.91E+00
DDT	protein_coding	2.37E-04	3.59E-03	-4.91E+00
CHMP6	protein_coding	1.56E-07	1.55E-05	-4.90E+00
EEF1A1P6	processed_pseudogene	2.46E-04	3.68E-03	-4.89E+00
PEG10	protein_coding	1.88E-05	6.08E-04	-4.89E+00
PLD3	protein_coding	1.42E-11	7.76E-09	-4.89E+00
NUCB1	protein_coding	9.23E-06	3.66E-04	-4.86E+00
LRPAP1	protein_coding	8.11E-10	2.42E-07	-4.83E+00
FDX1	protein_coding	5.70E-04	6.43E-03	-4.83E+00
CSF1R	protein_coding	1.43E-06	9.42E-05	-4.79E+00
CTSB	protein_coding	5.01E-05	1.20E-03	-4.78E+00
COX6A1	protein_coding	1.56E-09	3.96E-07	-4.78E+00
DDX28	protein_coding	4.91E-05	1.18E-03	-4.76E+00
TMBIM1	protein_coding	7.50E-09	1.35E-06	-4.74E+00
MAP1LC3B2	protein_coding	4.77E-04	5.76E-03	-4.74E+00
P4HB	protein_coding	3.55E-13	4.23E-10	-4.73E+00
OVOL1	protein_coding	1.63E-04	2.77E-03	-4.73E+00

DUSP4	protein_coding	7.52E-05	1.61E-03	-4.72E+00
ATP6V0B	protein_coding	1.86E-09	4.61E-07	-4.70E+00
ATAD1	protein_coding	9.22E-05	1.88E-03	-4.69E+00
SURF4	protein_coding	2.77E-13	3.52E-10	-4.69E+00
C15orf48	protein_coding	3.19E-05	8.75E-04	-4.68E+00
TMEM115	protein_coding	4.55E-04	5.57E-03	-4.65E+00
SLC2A1	protein_coding	2.74E-09	6.15E-07	-4.64E+00
ZNF593	protein_coding	1.08E-03	9.89E-03	-4.64E+00
EMC7	protein_coding	4.51E-08	5.94E-06	-4.63E+00
EMP2	protein_coding	4.68E-05	1.15E-03	-4.61E+00
SLC39A1	protein_coding	4.22E-06	2.09E-04	-4.60E+00
CRIM1	protein_coding	7.02E-04	7.46E-03	-4.57E+00
NT5E	protein_coding	6.31E-06	2.83E-04	-4.55E+00
SNX22	protein_coding	2.61E-04	3.83E-03	-4.55E+00
TMEM59	protein_coding	7.35E-06	3.13E-04	-4.51E+00
NDUFA13	protein_coding	2.02E-06	1.21E-04	-4.50E+00
ISYNA1	protein_coding	1.73E-04	2.90E-03	-4.50E+00
CALR	protein_coding	4.32E-10	1.47E-07	-4.50E+00
NAGA	protein_coding	3.75E-06	1.92E-04	-4.49E+00
CSHL1	protein_coding	1.83E-05	6.00E-04	-4.48E+00
TECR	protein_coding	1.36E-04	2.44E-03	-4.48E+00
UBAC2	protein_coding	8.02E-08	9.17E-06	-4.47E+00
HSD3B1	protein_coding	5.13E-04	6.02E-03	-4.46E+00
ARF1	protein_coding	7.69E-09	1.37E-06	-4.46E+00
GRAMD2B	protein_coding	1.17E-04	2.21E-03	-4.45E+00
ECM1	protein_coding	1.12E-04	2.14E-03	-4.45E+00
ARL6IP5	protein_coding	1.33E-12	1.15E-09	-4.45E+00
POP7	protein_coding	6.14E-05	1.39E-03	-4.44E+00
GM2A	protein_coding	2.41E-04	3.62E-03	-4.40E+00
PSENEN	protein_coding	2.64E-04	3.85E-03	-4.39E+00
CST3	protein_coding	3.76E-04	4.93E-03	-4.39E+00
ADGRE5	protein_coding	9.89E-05	1.98E-03	-4.38E+00
BAD	protein_coding	2.44E-05	7.17E-04	-4.36E+00
EEF1A1	protein_coding	1.56E-04	2.69E-03	-4.35E+00
PDIA3	protein_coding	2.29E-07	2.14E-05	-4.33E+00
CCDC47	protein_coding	2.16E-05	6.72E-04	-4.33E+00
ST3GAL4	protein_coding	4.37E-04	5.45E-03	-4.31E+00
HTATIP2	protein_coding	1.25E-05	4.61E-04	-4.31E+00
TMEM140	protein_coding	2.57E-06	1.45E-04	-4.31E+00

DDOST	protein_coding	2.28E-06	1.32E-04	-4.29E+00
TMBIM6	protein_coding	8.74E-08	9.75E-06	-4.27E+00
TAX1BP3	protein_coding	2.77E-07	2.52E-05	-4.26E+00
SYNGR2	protein_coding	8.69E-09	1.48E-06	-4.26E+00
SQSTM1	protein_coding	6.04E-05	1.37E-03	-4.24E+00
ATP6V0E1	protein_coding	4.63E-09	9.11E-07	-4.24E+00
SLC39A6	protein_coding	3.99E-04	5.13E-03	-4.23E+00
BEX2	protein_coding	8.28E-04	8.23E-03	-4.23E+00
MAFF	protein_coding	5.84E-04	6.53E-03	-4.21E+00
FEN1	protein_coding	5.06E-04	5.98E-03	-4.21E+00
HSP90B1	protein_coding	6.71E-07	5.12E-05	-4.18E+00
YIPF3	protein_coding	8.28E-07	6.00E-05	-4.17E+00
ZDHHC12	protein_coding	5.14E-04	6.02E-03	-4.16E+00
CD200	protein_coding	6.92E-04	7.37E-03	-4.15E+00
ORMDL3	protein_coding	1.78E-08	2.71E-06	-4.15E+00
SYPL1	protein_coding	1.12E-06	7.56E-05	-4.12E+00
CDK2AP2	protein_coding	4.29E-04	5.38E-03	-4.09E+00
LIPA	protein_coding	1.14E-05	4.30E-04	-4.08E+00
CISH	protein_coding	2.84E-05	8.02E-04	-4.08E+00
ADAMTSL4	protein_coding	5.64E-04	6.38E-03	-4.07E+00
SELENOF	protein_coding	4.78E-05	1.16E-03	-4.07E+00
ILVBL	protein_coding	1.11E-04	2.14E-03	-4.07E+00
PINK1	protein_coding	9.50E-04	9.11E-03	-4.05E+00
H2AZ1	protein_coding	6.79E-05	1.48E-03	-4.04E+00
STK26	protein_coding	1.08E-04	2.10E-03	-4.04E+00
SLC7A8	protein_coding	2.03E-04	3.21E-03	-4.02E+00
AP2M1	protein_coding	4.59E-10	1.54E-07	-4.02E+00
TMED1	protein_coding	5.78E-05	1.34E-03	-4.01E+00
PCNA	protein_coding	1.62E-04	2.76E-03	-4.00E+00
H4C3	protein_coding	2.62E-04	3.84E-03	-3.98E+00
SESN3	protein_coding	7.28E-04	7.61E-03	-3.98E+00
DIPK1A	protein_coding	1.30E-04	2.36E-03	-3.96E+00
S100A6	protein_coding	4.63E-11	2.19E-08	-3.94E+00
B4GALT1	protein_coding	1.05E-03	9.78E-03	-3.94E+00
PDIA4	protein_coding	8.43E-05	1.75E-03	-3.91E+00
HSD17B10	protein_coding	1.80E-05	5.94E-04	-3.91E+00
OLR1	protein_coding	1.49E-05	5.25E-04	-3.91E+00
NDUFA4	protein_coding	4.61E-08	6.03E-06	-3.90E+00
ATP6AP1	protein_coding	2.77E-06	1.53E-04	-3.90E+00

KDELR1	protein_coding	9.16E-06	3.64E-04	-3.89E+00
EHD1	protein_coding	1.72E-06	1.06E-04	-3.89E+00
CALU	protein_coding	1.97E-07	1.88E-05	-3.88E+00
REEP5	protein_coding	6.81E-08	8.02E-06	-3.88E+00
LAMP2	protein_coding	1.24E-06	8.22E-05	-3.87E+00
LGALS3BP	protein_coding	4.34E-04	5.42E-03	-3.86E+00
MAGEA10	protein_coding	2.96E-04	4.15E-03	-3.85E+00
COX6B1	protein_coding	7.53E-06	3.18E-04	-3.84E+00
PHB1	protein_coding	1.11E-04	2.14E-03	-3.84E+00
DDX49	protein_coding	6.35E-04	6.92E-03	-3.83E+00
GABARAPL1	protein_coding	2.03E-06	1.21E-04	-3.82E+00
NDUFC2	protein_coding	1.60E-06	1.01E-04	-3.82E+00
FOXO4	protein_coding	9.94E-05	1.98E-03	-3.82E+00
PLBD1	protein_coding	8.45E-05	1.76E-03	-3.81E+00
CNPPD1	protein_coding	8.37E-06	3.40E-04	-3.81E+00
COMT	protein_coding	2.15E-06	1.27E-04	-3.79E+00
CFL1	protein_coding	7.71E-05	1.64E-03	-3.78E+00
ERVH48-1	protein_coding	1.71E-06	1.06E-04	-3.78E+00
GSTP1	protein_coding	2.18E-05	6.75E-04	-3.78E+00
CD164	protein_coding	1.09E-09	2.96E-07	-3.77E+00
PTTG1IP	protein_coding	3.40E-10	1.25E-07	-3.77E+00
OSTC	protein_coding	1.71E-04	2.87E-03	-3.77E+00
TXNDC12	protein_coding	1.82E-05	5.97E-04	-3.76E+00
PFN1	protein_coding	7.27E-05	1.57E-03	-3.76E+00
ALDH4A1	protein_coding	4.40E-06	2.15E-04	-3.76E+00
CNPY2	protein_coding	5.24E-07	4.27E-05	-3.76E+00
CSRP1	protein_coding	1.14E-04	2.17E-03	-3.75E+00
GANAB	protein_coding	1.34E-04	2.42E-03	-3.75E+00
EVA1A	protein_coding	1.23E-04	2.28E-03	-3.74E+00
OS9	protein_coding	2.32E-05	6.99E-04	-3.73E+00
KRT18	protein_coding	1.31E-05	4.79E-04	-3.71E+00
FOLR1	protein_coding	5.36E-05	1.26E-03	-3.70E+00
ACTR6	protein_coding	3.49E-04	4.67E-03	-3.70E+00
ACP2	protein_coding	8.42E-06	3.41E-04	-3.69E+00
ALG3	protein_coding	1.73E-04	2.90E-03	-3.69E+00
SLC25A6	protein_coding	3.77E-08	5.18E-06	-3.67E+00
DYNLRB1	protein_coding	3.00E-04	4.18E-03	-3.67E+00
CDKN2AIP	protein_coding	4.42E-04	5.45E-03	-3.65E+00

SOD1	protein_coding	7.09E-06	3.05E-04	-3.65E+00
ACTG1	protein_coding	2.26E-05	6.88E-04	-3.64E+00
EGFR	protein_coding	2.73E-04	3.93E-03	-3.63E+00
CLIC1	protein_coding	2.56E-04	3.78E-03	-3.63E+00
AHCY	protein_coding	1.34E-05	4.86E-04	-3.62E+00
NUCB2	protein_coding	8.21E-04	8.20E-03	-3.61E+00
DOLK	protein_coding	7.23E-04	7.58E-03	-3.59E+00
RTN3	protein_coding	4.50E-08	5.94E-06	-3.58E+00
NXT2	protein_coding	2.97E-04	4.16E-03	-3.58E+00
LMAN2	protein_coding	5.74E-09	1.08E-06	-3.57E+00
CYB5R1	protein_coding	3.94E-05	1.02E-03	-3.57E+00
MESD	protein_coding	3.26E-07	2.92E-05	-3.56E+00
H3-3A	protein_coding	7.88E-05	1.67E-03	-3.56E+00
AMDHD2	protein_coding	6.96E-05	1.51E-03	-3.55E+00
TFPI	protein_coding	9.84E-04	9.29E-03	-3.55E+00
PIGH	protein_coding	1.39E-04	2.47E-03	-3.54E+00
VPS25	protein_coding	2.17E-04	3.37E-03	-3.52E+00
TXNDC9	protein_coding	6.30E-05	1.41E-03	-3.52E+00
RHOG	protein_coding	9.12E-07	6.44E-05	-3.51E+00
SERF2	protein_coding	2.31E-05	6.98E-04	-3.50E+00
ST3GAL1	protein_coding	2.28E-04	3.48E-03	-3.50E+00
SCAMP2	protein_coding	4.39E-09	8.73E-07	-3.50E+00
RCN1	protein_coding	2.42E-09	5.57E-07	-3.50E+00
EDEM2	protein_coding	2.93E-06	1.59E-04	-3.49E+00
SMIM29	protein_coding	1.02E-04	2.02E-03	-3.48E+00
PLIN3	protein_coding	1.04E-05	3.99E-04	-3.47E+00
NSG1	protein_coding	7.19E-04	7.55E-03	-3.47E+00
TMEM40	protein_coding	2.31E-04	3.51E-03	-3.47E+00
CANX	protein_coding	2.35E-05	7.05E-04	-3.47E+00
SLC35A2	protein_coding	3.08E-04	4.25E-03	-3.46E+00
IARS2	protein_coding	8.38E-06	3.40E-04	-3.46E+00
C8orf76	protein_coding	3.70E-04	4.87E-03	-3.46E+00
TMED4	protein_coding	1.03E-05	3.95E-04	-3.46E+00
RASL11B	protein_coding	2.55E-04	3.76E-03	-3.46E+00
MYL12B	protein_coding	3.21E-05	8.80E-04	-3.44E+00
ATOX1	protein_coding	1.23E-04	2.29E-03	-3.44E+00
CD59	protein_coding	2.17E-06	1.27E-04	-3.43E+00
PISD	protein_coding	8.13E-09	1.42E-06	-3.43E+00
ITGB5	protein_coding	7.95E-04	8.05E-03	-3.43E+00

KIAA1522	protein_coding	1.70E-04	2.86E-03	-3.42E+00
HEXIM1	protein_coding	4.40E-04	5.45E-03	-3.42E+00
SLC44A2	protein_coding	4.99E-06	2.38E-04	-3.42E+00
CDH1	protein_coding	2.60E-05	7.48E-04	-3.41E+00
FIS1	protein_coding	1.43E-07	1.46E-05	-3.41E+00
MLF2	protein_coding	3.89E-05	1.01E-03	-3.41E+00
TMEM101	protein_coding	6.61E-04	7.14E-03	-3.41E+00
NR1H2	protein_coding	1.75E-05	5.84E-04	-3.40E+00
RABIF	protein_coding	1.09E-03	9.97E-03	-3.40E+00
PRDX5	protein_coding	3.29E-07	2.93E-05	-3.38E+00
ERP29	protein_coding	1.03E-09	2.88E-07	-3.36E+00
SARAF	protein_coding	2.60E-04	3.81E-03	-3.36E+00
DEPP1	protein_coding	3.50E-07	3.06E-05	-3.36E+00
ARF4	protein_coding	7.46E-04	7.73E-03	-3.35E+00
SF3B6	protein_coding	1.45E-05	5.14E-04	-3.35E+00
DNAJC3	protein_coding	5.22E-05	1.23E-03	-3.35E+00
IFNGR1	protein_coding	7.90E-06	3.30E-04	-3.34E+00
RASA1	protein_coding	3.69E-04	4.86E-03	-3.33E+00
SCAMP3	protein_coding	1.31E-06	8.67E-05	-3.33E+00
VGLL1	protein_coding	1.11E-05	4.22E-04	-3.32E+00
SRPRA	protein_coding	3.41E-04	4.59E-03	-3.32E+00
YIF1A	protein_coding	6.63E-06	2.91E-04	-3.32E+00
PSMB6	protein_coding	1.62E-05	5.53E-04	-3.32E+00
TKT	protein_coding	2.55E-09	5.78E-07	-3.31E+00
BCAP31	protein_coding	1.68E-08	2.61E-06	-3.31E+00
RBX1	protein_coding	2.02E-06	1.21E-04	-3.30E+00
ALDOA	protein_coding	9.42E-08	1.04E-05	-3.30E+00
PIGY	protein_coding	1.07E-03	9.86E-03	-3.28E+00
PYURF	protein_coding	1.07E-03	9.86E-03	-3.28E+00
SMAGP	protein_coding	6.65E-04	7.17E-03	-3.28E+00
NDUFB11	protein_coding	4.05E-05	1.04E-03	-3.28E+00
MRPL36	protein_coding	3.15E-04	4.31E-03	-3.28E+00
DAD1	protein_coding	1.77E-05	5.88E-04	-3.28E+00
NDUFB7	protein_coding	4.13E-05	1.05E-03	-3.27E+00
HACD3	protein_coding	2.88E-06	1.58E-04	-3.26E+00
FAUP1	processed_pseudogene	1.22E-04	2.28E-03	-3.26E+00
ADIPOR1	protein_coding	1.88E-04	3.06E-03	-3.26E+00
ATP1B3	protein_coding	1.17E-07	1.22E-05	-3.26E+00

TMEM139	protein_coding	3.80E-04	4.97E-03	-3.25E+00
TMEM109	protein_coding	8.41E-08	9.47E-06	-3.24E+00
PLPP3	protein_coding	9.55E-04	9.14E-03	-3.23E+00
TOLLIP	protein_coding	5.09E-05	1.21E-03	-3.23E+00
TMEM179B	protein_coding	1.82E-04	3.00E-03	-3.23E+00
NDUFA11	protein_coding	2.25E-06	1.31E-04	-3.23E+00
NUDT16L1	protein_coding	6.39E-05	1.42E-03	-3.23E+00
IRF6	protein_coding	4.13E-04	5.25E-03	-3.23E+00
DNAJC8	protein_coding	6.19E-07	4.82E-05	-3.22E+00
NDUFA1	protein_coding	4.10E-07	3.46E-05	-3.21E+00
APMAP	protein_coding	2.09E-05	6.52E-04	-3.21E+00
MPDU1	protein_coding	1.72E-06	1.06E-04	-3.21E+00
TMEM208	protein_coding	1.04E-04	2.04E-03	-3.20E+00
ITFG1	protein_coding	5.18E-05	1.22E-03	-3.20E+00
TOR1A	protein_coding	3.93E-04	5.07E-03	-3.20E+00
SNRPB	protein_coding	1.39E-05	4.96E-04	-3.20E+00
B4GALT3	protein_coding	8.89E-05	1.83E-03	-3.19E+00
ATP5PO	protein_coding	9.61E-05	1.94E-03	-3.19E+00
RHBDD2	protein_coding	4.08E-04	5.22E-03	-3.19E+00
C1GALT1	protein_coding	1.08E-04	2.10E-03	-3.19E+00
GLMP	protein_coding	2.08E-05	6.51E-04	-3.18E+00
SND1	protein_coding	4.25E-07	3.54E-05	-3.18E+00
ATRAID	protein_coding	1.03E-04	2.04E-03	-3.17E+00
HSPA8	protein_coding	8.53E-04	8.43E-03	-3.17E+00
LMNA	protein_coding	7.86E-05	1.67E-03	-3.16E+00
ADGRG1	protein_coding	5.32E-04	6.14E-03	-3.16E+00
GPX1	protein_coding	8.78E-04	8.57E-03	-3.16E+00
ATIC	protein_coding	8.39E-05	1.75E-03	-3.15E+00
WDR83OS	protein_coding	1.37E-04	2.44E-03	-3.14E+00
RAB1B	protein_coding	9.20E-05	1.88E-03	-3.13E+00
SSR2	protein_coding	3.27E-06	1.73E-04	-3.13E+00
NINJ1	protein_coding	1.65E-04	2.80E-03	-3.12E+00
AKR1B1	protein_coding	7.49E-05	1.60E-03	-3.11E+00
ATP6AP2	protein_coding	8.48E-05	1.76E-03	-3.11E+00
SERTAD1	protein_coding	4.78E-06	2.29E-04	-3.10E+00
NUDT22	protein_coding	1.94E-04	3.11E-03	-3.10E+00
TALDO1	protein_coding	8.72E-04	8.54E-03	-3.09E+00
SNRPD2	protein_coding	1.45E-04	2.54E-03	-3.09E+00
TAGLN2	protein_coding	5.11E-04	6.01E-03	-3.09E+00

GULP1	protein_coding	2.77E-04	3.96E-03	-3.08E+00
FAU	protein_coding	1.28E-06	8.49E-05	-3.08E+00
GNG12	protein_coding	8.36E-05	1.74E-03	-3.07E+00
ERGIC3	protein_coding	2.38E-05	7.09E-04	-3.07E+00
FKBP4	protein_coding	4.32E-04	5.41E-03	-3.06E+00
BRMS1	protein_coding	5.56E-05	1.30E-03	-3.05E+00
GNS	protein_coding	4.93E-07	4.06E-05	-3.05E+00
CRYBG1	protein_coding	7.13E-04	7.53E-03	-3.04E+00
TMEM254	protein_coding	1.84E-04	3.03E-03	-3.04E+00
TMEM141	protein_coding	6.17E-05	1.39E-03	-3.03E+00
ELOB	protein_coding	2.78E-05	7.93E-04	-3.03E+00
COPE	protein_coding	5.53E-07	4.40E-05	-3.03E+00
PARK7	protein_coding	7.51E-06	3.18E-04	-3.03E+00
SERINC3	protein_coding	7.97E-06	3.32E-04	-3.03E+00
TMEM9B	protein_coding	1.05E-04	2.06E-03	-3.03E+00
RNASEK	protein_coding	4.07E-07	3.45E-05	-3.03E+00
KRT80	protein_coding	7.74E-04	7.92E-03	-3.02E+00
SEM1	protein_coding	5.46E-06	2.55E-04	-3.01E+00
ATG9A	protein_coding	6.04E-04	6.70E-03	-3.01E+00
NDUFA2	protein_coding	3.34E-05	9.08E-04	-3.01E+00
ARL6IP1	protein_coding	2.72E-06	1.51E-04	-3.00E+00
KLHDC3	protein_coding	3.85E-04	5.01E-03	-3.00E+00
PGRMC1	protein_coding	3.66E-04	4.84E-03	-3.00E+00
TMED2	protein_coding	3.23E-04	4.39E-03	-3.00E+00
TMEM222	protein_coding	1.10E-04	2.13E-03	-2.98E+00
COX7C	protein_coding	6.41E-07	4.95E-05	-2.98E+00
SPCS2	protein_coding	3.44E-05	9.22E-04	-2.98E+00
NUDC	protein_coding	2.98E-06	1.61E-04	-2.98E+00
ACOT7	protein_coding	5.96E-04	6.62E-03	-2.97E+00
GPAA1	protein_coding	4.14E-05	1.05E-03	-2.97E+00
TPD52	protein_coding	2.21E-04	3.41E-03	-2.97E+00
BANF1	protein_coding	6.34E-04	6.92E-03	-2.94E+00
YIPF6	protein_coding	1.42E-04	2.50E-03	-2.94E+00
CD24	protein_coding	8.48E-05	1.76E-03	-2.94E+00
PBXIP1	protein_coding	9.64E-04	9.17E-03	-2.93E+00
SELENOT	protein_coding	3.13E-04	4.29E-03	-2.93E+00
BCKDHA	protein_coding	5.13E-05	1.22E-03	-2.93E+00
ARPC5	protein_coding	2.05E-07	1.94E-05	-2.93E+00
ATP5F1E	protein_coding	2.43E-05	7.14E-04	-2.92E+00

TM9SF1	protein_coding	4.80E-06	2.30E-04	-2.92E+00
XAGE2	protein_coding	1.01E-04	2.00E-03	-2.90E+00
POLR2E	protein_coding	3.15E-06	1.68E-04	-2.90E+00
SUMF1	protein_coding	3.94E-05	1.02E-03	-2.90E+00
IER3IP1	protein_coding	8.13E-04	8.14E-03	-2.90E+00
FSTL1	protein_coding	2.36E-06	1.35E-04	-2.90E+00
NELFE	protein_coding	3.76E-06	1.92E-04	-2.90E+00
MCFD2	protein_coding	1.91E-04	3.09E-03	-2.89E+00
NAPA	protein_coding	1.03E-04	2.03E-03	-2.89E+00
EBNA1BP2	protein_coding	5.81E-04	6.51E-03	-2.88E+00
BLOC1S1	protein_coding	2.63E-04	3.85E-03	-2.88E+00
NRK	protein_coding	8.74E-04	8.55E-03	-2.87E+00
PCBP1	protein_coding	8.10E-06	3.35E-04	-2.87E+00
MGST3	protein_coding	8.27E-05	1.73E-03	-2.86E+00
SIAH1	protein_coding	2.21E-04	3.42E-03	-2.86E+00
TUFM	protein_coding	3.70E-05	9.72E-04	-2.86E+00
BCAT2	protein_coding	1.10E-04	2.12E-03	-2.85E+00
COX4I1	protein_coding	1.27E-05	4.68E-04	-2.85E+00
SLC29A3	protein_coding	1.04E-03	9.69E-03	-2.85E+00
GINM1	protein_coding	9.28E-05	1.89E-03	-2.85E+00
PAQR7	protein_coding	8.13E-04	8.14E-03	-2.85E+00
UQCRC1	protein_coding	3.22E-04	4.38E-03	-2.84E+00
COX5B	protein_coding	8.65E-06	3.48E-04	-2.84E+00
SLC35F6	protein_coding	2.05E-04	3.23E-03	-2.84E+00
SLC16A4	protein_coding	5.47E-04	6.28E-03	-2.83E+00
NDUFB6	protein_coding	3.59E-04	4.76E-03	-2.83E+00
MARCKS	protein_coding	9.79E-04	9.27E-03	-2.82E+00
CMTM6	protein_coding	1.42E-04	2.51E-03	-2.82E+00
BTG2	protein_coding	1.93E-04	3.11E-03	-2.82E+00
KDELR2	protein_coding	2.97E-11	1.49E-08	-2.82E+00
SCYL1	protein_coding	1.53E-05	5.30E-04	-2.81E+00
CUTA	protein_coding	6.10E-05	1.38E-03	-2.80E+00
NDUFS5	protein_coding	6.45E-05	1.43E-03	-2.79E+00
ANXA6	protein_coding	3.07E-04	4.25E-03	-2.79E+00
ANXA5	protein_coding	1.45E-04	2.54E-03	-2.78E+00
RNF167	protein_coding	4.05E-04	5.18E-03	-2.78E+00
HMOX1	protein_coding	4.17E-04	5.28E-03	-2.78E+00
RSL24D1	protein_coding	5.61E-04	6.36E-03	-2.78E+00
BEX4	protein_coding	6.98E-04	7.43E-03	-2.77E+00

EIF1	protein_coding	8.53E-07	6.15E-05	-2.76E+00
BTF3	protein_coding	4.51E-05	1.12E-03	-2.76E+00
SMIM26	protein_coding	6.76E-04	7.26E-03	-2.76E+00
BCAP29	protein_coding	2.62E-04	3.84E-03	-2.75E+00
COX7A2	protein_coding	7.74E-08	8.95E-06	-2.75E+00
TPP1	protein_coding	8.06E-06	3.34E-04	-2.75E+00
MORF4L1	protein_coding	3.28E-06	1.73E-04	-2.75E+00
ATP5PF	protein_coding	2.12E-04	3.31E-03	-2.74E+00
C6orf89	protein_coding	1.44E-06	9.43E-05	-2.74E+00
RNF181	protein_coding	2.54E-04	3.76E-03	-2.73E+00
GABARAPL2	protein_coding	5.14E-08	6.49E-06	-2.73E+00
FAM234A	protein_coding	5.72E-04	6.44E-03	-2.73E+00
EDF1	protein_coding	2.20E-04	3.41E-03	-2.73E+00
SHISA5	protein_coding	5.82E-05	1.35E-03	-2.73E+00
PSMC3	protein_coding	4.99E-05	1.20E-03	-2.72E+00
SNX17	protein_coding	9.78E-06	3.82E-04	-2.72E+00
PPP2R1A	protein_coding	3.28E-05	8.94E-04	-2.72E+00
HSP90AA1	protein_coding	1.21E-05	4.50E-04	-2.72E+00
PTPMT1	protein_coding	5.51E-05	1.29E-03	-2.71E+00
TNFAIP1	protein_coding	8.07E-05	1.70E-03	-2.71E+00
POMP	protein_coding	4.40E-04	5.45E-03	-2.71E+00
NPM1	protein_coding	2.29E-04	3.48E-03	-2.71E+00
ASAH1	protein_coding	4.50E-05	1.12E-03	-2.69E+00
AURKAIP1	protein_coding	3.51E-04	4.68E-03	-2.68E+00
PRPF31	protein_coding	9.06E-04	8.79E-03	-2.68E+00
PTGES3	protein_coding	8.45E-08	9.47E-06	-2.67E+00
B4GALT7	protein_coding	1.25E-04	2.31E-03	-2.67E+00
CCT7	protein_coding	8.04E-04	8.10E-03	-2.66E+00
STT3A	protein_coding	3.21E-05	8.81E-04	-2.66E+00
PSMD8	protein_coding	8.57E-06	3.45E-04	-2.65E+00
UQCRQ	protein_coding	2.55E-05	7.41E-04	-2.65E+00
FAM168B	protein_coding	9.75E-04	9.24E-03	-2.64E+00
TUBB4B	protein_coding	5.93E-07	4.64E-05	-2.63E+00
UQCRFS1	protein_coding	2.48E-04	3.70E-03	-2.63E+00
PUF60	protein_coding	7.82E-06	3.27E-04	-2.63E+00
PSMA4	protein_coding	3.27E-04	4.44E-03	-2.63E+00
ATP5PB	protein_coding	2.86E-04	4.05E-03	-2.62E+00
VCP	protein_coding	5.53E-07	4.40E-05	-2.62E+00

RNF13	protein_coding	2.40E-06	1.37E-04	-2.62E+00
NEU1	protein_coding	1.03E-03	9.56E-03	-2.62E+00
MDH2	protein_coding	5.00E-04	5.93E-03	-2.61E+00
CCT3	protein_coding	5.44E-04	6.25E-03	-2.61E+00
AP2S1	protein_coding	1.42E-04	2.50E-03	-2.61E+00
PDLIM2	protein_coding	3.88E-04	5.03E-03	-2.61E+00
ECH1	protein_coding	4.50E-04	5.52E-03	-2.61E+00
ANAPC11	protein_coding	6.34E-05	1.42E-03	-2.60E+00
ATP5MF	protein_coding	1.20E-04	2.26E-03	-2.59E+00
SGTA	protein_coding	4.96E-04	5.90E-03	-2.59E+00
RETREG3	protein_coding	2.43E-04	3.66E-03	-2.59E+00
PFKL	protein_coding	2.28E-05	6.92E-04	-2.59E+00
ANXA1	protein_coding	6.17E-04	6.79E-03	-2.58E+00
PLEKHO2	protein_coding	2.32E-05	6.98E-04	-2.57E+00
COX5A	protein_coding	2.76E-04	3.96E-03	-2.57E+00
SLC25A11	protein_coding	6.32E-04	6.91E-03	-2.56E+00
AP1S1	protein_coding	9.18E-04	8.86E-03	-2.56E+00
ACTR1A	protein_coding	4.55E-05	1.13E-03	-2.56E+00
PMF1	protein_coding	1.25E-04	2.31E-03	-2.56E+00
TXNDC17	protein_coding	4.39E-04	5.45E-03	-2.54E+00
TPD52L2	protein_coding	1.17E-04	2.22E-03	-2.54E+00
EEF1G	protein_coding	7.40E-04	7.70E-03	-2.54E+00
H3P6	processed_pseudogene	3.24E-06	1.72E-04	-2.54E+00
TMEM50A	protein_coding	1.31E-04	2.38E-03	-2.53E+00
COX7B	protein_coding	9.19E-05	1.88E-03	-2.53E+00
RAB1A	protein_coding	2.71E-04	3.93E-03	-2.53E+00
ATP6V0D1	protein_coding	3.02E-05	8.45E-04	-2.52E+00
MAP2K3	protein_coding	9.83E-04	9.28E-03	-2.52E+00
MAP1LC3B	protein_coding	9.19E-05	1.88E-03	-2.51E+00
GDE1	protein_coding	4.75E-06	2.29E-04	-2.51E+00
PSMB3	protein_coding	8.03E-05	1.69E-03	-2.51E+00
HNRNPC	protein_coding	1.24E-04	2.31E-03	-2.50E+00
FBXW5	protein_coding	3.91E-04	5.06E-03	-2.50E+00
ATP5MG	protein_coding	5.68E-05	1.32E-03	-2.50E+00
LYPLA2	protein_coding	4.78E-04	5.76E-03	-2.50E+00
TMEM258	protein_coding	8.60E-04	8.46E-03	-2.50E+00
SZRD1	protein_coding	2.06E-04	3.24E-03	-2.49E+00
AP1B1	protein_coding	4.83E-05	1.17E-03	-2.49E+00

EID1	protein_coding	7.18E-04	7.55E-03	-2.49E+00
MRPL15	protein_coding	1.30E-04	2.37E-03	-2.48E+00
NAA38	protein_coding	9.58E-04	9.14E-03	-2.48E+00
LEPROT	protein_coding	1.89E-06	1.14E-04	-2.47E+00
DERPC	protein_coding	8.12E-05	1.71E-03	-2.47E+00
UQCR11	protein_coding	8.62E-05	1.78E-03	-2.47E+00
APLP2	protein_coding	1.36E-04	2.44E-03	-2.45E+00
SSR1	protein_coding	1.83E-08	2.77E-06	-2.45E+00
ELOVL1	protein_coding	1.05E-03	9.78E-03	-2.45E+00
RHOC	protein_coding	1.32E-04	2.39E-03	-2.44E+00
ERLEC1	protein_coding	7.40E-07	5.54E-05	-2.44E+00
ADRM1	protein_coding	9.83E-05	1.97E-03	-2.43E+00
ATP5MC1	protein_coding	8.54E-04	8.43E-03	-2.43E+00
NDUFB2	protein_coding	4.20E-05	1.07E-03	-2.43E+00
LRRC41	protein_coding	2.20E-04	3.41E-03	-2.42E+00
PSMB1	protein_coding	5.87E-05	1.35E-03	-2.41E+00
CLIC4	protein_coding	9.63E-04	9.17E-03	-2.41E+00
TMX2	protein_coding	9.16E-04	8.86E-03	-2.41E+00
UQCRB	protein_coding	1.69E-05	5.70E-04	-2.40E+00
UBE2L3	protein_coding	9.50E-04	9.11E-03	-2.39E+00
ATP6V1G1	protein_coding	2.73E-04	3.93E-03	-2.39E+00
PPP1CA	protein_coding	7.92E-04	8.03E-03	-2.38E+00
SPCS1	protein_coding	1.51E-04	2.63E-03	-2.38E+00
CHCHD2	protein_coding	1.45E-04	2.54E-03	-2.38E+00
HNRNPK	protein_coding	1.70E-04	2.86E-03	-2.37E+00
TMEM43	protein_coding	1.01E-03	9.48E-03	-2.36E+00
DAXX	protein_coding	1.06E-03	9.81E-03	-2.36E+00
ATP5MK	protein_coding	1.82E-04	3.00E-03	-2.36E+00
CREB3	protein_coding	1.00E-03	9.39E-03	-2.33E+00
DNAJA1	protein_coding	8.70E-04	8.53E-03	-2.33E+00
APOL1	protein_coding	2.48E-04	3.69E-03	-2.32E+00
TBCA	protein_coding	7.90E-04	8.02E-03	-2.32E+00
HSP90AB1	protein_coding	4.08E-05	1.04E-03	-2.31E+00
TMEM106B	protein_coding	6.23E-05	1.40E-03	-2.30E+00
KLF10	protein_coding	6.39E-04	6.94E-03	-2.30E+00
RMDN3	protein_coding	5.12E-04	6.01E-03	-2.29E+00
PDCD10	protein_coding	1.07E-03	9.85E-03	-2.29E+00
PRDX1	protein_coding	5.93E-05	1.36E-03	-2.28E+00
PTMA	protein_coding	6.46E-04	7.00E-03	-2.27E+00

ATP5MJ	protein_coding	4.88E-04	5.84E-03	-2.27E+00
TRIM29	protein_coding	2.74E-05	7.81E-04	-2.27E+00
MCRIP1	protein_coding	9.39E-06	3.72E-04	-2.27E+00
ARL1	protein_coding	3.59E-05	9.51E-04	-2.27E+00
CDC42SE1	protein_coding	4.80E-04	5.77E-03	-2.26E+00
QPRT	protein_coding	3.39E-05	9.16E-04	-2.25E+00
DERL1	protein_coding	7.52E-04	7.76E-03	-2.24E+00
FDFT1	protein_coding	5.19E-04	6.05E-03	-2.23E+00
TSG101	protein_coding	9.36E-04	9.02E-03	-2.23E+00
COPG1	protein_coding	1.37E-04	2.44E-03	-2.23E+00
SEC61A1	protein_coding	7.82E-07	5.78E-05	-2.21E+00
BSDC1	protein_coding	2.44E-04	3.66E-03	-2.21E+00
SNRPG	protein_coding	3.29E-04	4.45E-03	-2.21E+00
EEF2	protein_coding	4.87E-04	5.84E-03	-2.20E+00
ZNF706	protein_coding	6.57E-04	7.10E-03	-2.19E+00
FARSA	protein_coding	1.22E-04	2.27E-03	-2.19E+00
TRMT112	protein_coding	5.59E-04	6.34E-03	-2.19E+00
EIF4H	protein_coding	1.29E-04	2.36E-03	-2.19E+00
VPS35	protein_coding	7.37E-04	7.68E-03	-2.17E+00
RAB2A	protein_coding	9.49E-04	9.11E-03	-2.17E+00
TAF7	protein_coding	6.23E-04	6.82E-03	-2.17E+00
SLC35A4	protein_coding	1.26E-04	2.33E-03	-2.16E+00
TPT1	protein_coding	9.40E-04	9.04E-03	-2.15E+00
TM2D3	protein_coding	3.77E-04	4.93E-03	-2.14E+00
SERBP1	protein_coding	3.17E-04	4.33E-03	-2.12E+00
UBL5	protein_coding	8.03E-04	8.10E-03	-2.11E+00
SEC11A	protein_coding	4.87E-04	5.84E-03	-2.10E+00
TMEM87A	protein_coding	5.26E-04	6.10E-03	-2.09E+00
EIF1AX	protein_coding	7.85E-04	7.98E-03	-2.08E+00
CTNNA1	protein_coding	9.11E-05	1.87E-03	-2.06E+00
PKM	protein_coding	7.13E-04	7.53E-03	-2.05E+00
HSPA9	protein_coding	5.79E-04	6.50E-03	-2.04E+00
DNAJB12	protein_coding	7.60E-04	7.83E-03	-2.03E+00
HADHA	protein_coding	3.90E-04	5.05E-03	-2.01E+00
TSC1	protein_coding	7.68E-04	7.88E-03	2.01E+00
ANKLE2	protein_coding	7.42E-04	7.72E-03	2.02E+00
UGGT2	protein_coding	5.57E-04	6.34E-03	2.03E+00
ATRX	protein_coding	8.89E-04	8.65E-03	2.03E+00
CCDC82	protein_coding	7.36E-04	7.68E-03	2.04E+00

AP3B1	protein_coding	2.22E-04	3.42E-03	2.07E+00
LIMA1	protein_coding	4.51E-04	5.53E-03	2.07E+00
ZMIZ1	protein_coding	5.54E-04	6.33E-03	2.08E+00
PHKA2	protein_coding	4.41E-04	5.45E-03	2.09E+00
FMNL2	protein_coding	2.23E-04	3.43E-03	2.10E+00
ARGLU1	protein_coding	1.21E-04	2.27E-03	2.11E+00
HDAC6	protein_coding	3.74E-04	4.91E-03	2.11E+00
SUN1	protein_coding	5.07E-05	1.21E-03	2.12E+00
VPS13C	protein_coding	4.28E-06	2.10E-04	2.14E+00
LZTR1	protein_coding	1.54E-04	2.67E-03	2.15E+00
SMC5	protein_coding	1.02E-03	9.55E-03	2.15E+00
CENPC	protein_coding	3.53E-04	4.70E-03	2.15E+00
ZFHX3	protein_coding	4.88E-04	5.84E-03	2.17E+00
SASH1	protein_coding	1.85E-05	6.02E-04	2.17E+00
NADSYN1	protein_coding	8.29E-04	8.24E-03	2.19E+00
MACF1	protein_coding	6.04E-05	1.37E-03	2.19E+00
RNF213	protein_coding	1.88E-05	6.08E-04	2.20E+00
TIA1	protein_coding	6.12E-04	6.75E-03	2.21E+00
UTRN	protein_coding	1.88E-05	6.08E-04	2.21E+00
PCNX1	protein_coding	3.25E-04	4.41E-03	2.22E+00
CDK5RAP3	protein_coding	2.38E-05	7.09E-04	2.22E+00
AGAP3	protein_coding	5.13E-04	6.02E-03	2.24E+00
RBM5	protein_coding	8.62E-05	1.78E-03	2.24E+00
MED17	protein_coding	7.30E-04	7.63E-03	2.25E+00
CEP104	protein_coding	2.74E-04	3.94E-03	2.25E+00
KDM1A	protein_coding	8.21E-04	8.20E-03	2.25E+00
SMPD4BP	transcribed_processed_ pseudogene	1.26E-04	2.33E-03	2.25E+00
USP8	protein_coding	4.41E-04	5.45E-03	2.26E+00
FLCN	protein_coding	1.79E-04	2.97E-03	2.28E+00
EFHC1	protein_coding	9.89E-04	9.32E-03	2.28E+00
PCNT	protein_coding	1.07E-04	2.09E-03	2.29E+00
SMARCA4	protein_coding	9.57E-04	9.14E-03	2.30E+00
NCOR1	protein_coding	2.55E-06	1.44E-04	2.30E+00
INTS3	protein_coding	2.70E-05	7.74E-04	2.30E+00
PXN	protein_coding	1.75E-06	1.07E-04	2.30E+00
ZKSCAN1	protein_coding	1.66E-06	1.03E-04	2.31E+00
CFLAR	protein_coding	5.57E-04	6.34E-03	2.31E+00
ZFAS1	lncRNA	1.08E-03	9.94E-03	2.31E+00

CEP152	protein_coding	8.29E-05	1.73E-03	2.32E+00
TUBGCP6	protein_coding	8.23E-04	8.21E-03	2.32E+00
CRYBG3	protein_coding	1.00E-03	9.39E-03	2.33E+00
TTC37	protein_coding	1.07E-06	7.32E-05	2.33E+00
AQR	protein_coding	3.70E-04	4.88E-03	2.33E+00
FER	protein_coding	1.18E-04	2.22E-03	2.35E+00
CDK10	protein_coding	2.19E-05	6.76E-04	2.36E+00
TBCD	protein_coding	1.03E-03	9.57E-03	2.37E+00
MPHOSPH8	protein_coding	1.64E-05	5.57E-04	2.37E+00
ING5	protein_coding	1.85E-04	3.04E-03	2.37E+00
FIRRE	lncRNA	1.02E-03	9.50E-03	2.37E+00
FRYL	protein_coding	4.98E-05	1.20E-03	2.38E+00
PNN	protein_coding	5.96E-04	6.62E-03	2.38E+00
SPG11	protein_coding	4.38E-04	5.45E-03	2.39E+00
DMTF1	protein_coding	2.45E-05	7.18E-04	2.40E+00
TAFAZZIN	protein_coding	3.31E-04	4.48E-03	2.41E+00
NSRP1	protein_coding	1.86E-04	3.04E-03	2.42E+00
ATG16L2	protein_coding	9.90E-04	9.32E-03	2.42E+00
HECTD4	protein_coding	1.95E-04	3.12E-03	2.42E+00
HERC4	protein_coding	7.77E-04	7.94E-03	2.43E+00
INTS4	protein_coding	3.84E-05	1.00E-03	2.44E+00
SMCHD1	protein_coding	1.74E-04	2.90E-03	2.44E+00
KANSL3	protein_coding	5.36E-06	2.52E-04	2.45E+00
MAGI3	protein_coding	6.12E-04	6.75E-03	2.45E+00
KAT6B	protein_coding	3.21E-06	1.70E-04	2.46E+00
PI4KAP2	transcribed_unitary_pseudogene	4.47E-04	5.49E-03	2.47E+00
SCML1	protein_coding	2.91E-04	4.10E-03	2.47E+00
NISCH	protein_coding	3.00E-04	4.18E-03	2.48E+00
ASXL1	protein_coding	5.40E-05	1.26E-03	2.49E+00
POGZ	protein_coding	6.48E-06	2.85E-04	2.49E+00
CENATAC	protein_coding	1.97E-04	3.15E-03	2.49E+00
OGT	protein_coding	1.00E-06	6.98E-05	2.49E+00
ALPK1	protein_coding	1.06E-03	9.78E-03	2.50E+00
ERCC5	protein_coding	1.20E-04	2.25E-03	2.50E+00
MAPKAPK5	protein_coding	1.03E-04	2.04E-03	2.52E+00
ARHGEF17	protein_coding	8.31E-06	3.39E-04	2.52E+00
TUT4	protein_coding	2.47E-05	7.22E-04	2.52E+00
LPIN1	protein_coding	5.55E-04	6.34E-03	2.53E+00

HCG18	lncRNA	2.11E-06	1.25E-04	2.54E+00
DMD	protein_coding	9.66E-05	1.94E-03	2.54E+00
CCDC57	protein_coding	8.82E-04	8.59E-03	2.55E+00
MIR4435-2HG	lncRNA	3.85E-06	1.96E-04	2.55E+00
CLCN6	protein_coding	9.23E-04	8.90E-03	2.56E+00
TAF1D	protein_coding	1.54E-04	2.67E-03	2.56E+00
AGO4	protein_coding	4.61E-04	5.64E-03	2.56E+00
CHD9	protein_coding	6.46E-08	7.70E-06	2.57E+00
MBD5	protein_coding	2.93E-04	4.12E-03	2.57E+00
ITPR2	protein_coding	1.04E-04	2.05E-03	2.57E+00
SP100	protein_coding	1.87E-04	3.06E-03	2.58E+00
CNTRL	protein_coding	2.65E-04	3.86E-03	2.58E+00
FARP1	protein_coding	7.70E-04	7.88E-03	2.58E+00
ZCCHC8	protein_coding	4.18E-04	5.29E-03	2.58E+00
PIGN	protein_coding	1.04E-03	9.70E-03	2.58E+00
HECW2	protein_coding	8.15E-04	8.15E-03	2.59E+00
ZNF639	protein_coding	5.29E-04	6.13E-03	2.59E+00
PHF6	protein_coding	5.16E-04	6.03E-03	2.60E+00
AFF3	protein_coding	2.80E-04	4.00E-03	2.60E+00
LUC7L2	protein_coding	3.15E-06	1.68E-04	2.60E+00
FAM193B	protein_coding	1.36E-05	4.91E-04	2.60E+00
SPG7	protein_coding	7.14E-10	2.21E-07	2.60E+00
CARS2	protein_coding	9.15E-04	8.86E-03	2.60E+00
CHD7	protein_coding	5.13E-05	1.22E-03	2.61E+00
PNISR	protein_coding	6.52E-05	1.44E-03	2.61E+00
CCDC18-AS1	lncRNA	1.94E-04	3.12E-03	2.61E+00
ARHGAP29	protein_coding	2.39E-04	3.60E-03	2.62E+00
ZSCAN30	protein_coding	3.17E-05	8.73E-04	2.63E+00
GABPB2	protein_coding	6.18E-04	6.79E-03	2.64E+00
ANKZF1	protein_coding	1.27E-04	2.34E-03	2.64E+00
CAPN10	protein_coding	1.06E-03	9.79E-03	2.65E+00
EIF4G3	protein_coding	8.16E-04	8.16E-03	2.65E+00
ZNF814	protein_coding	1.36E-04	2.44E-03	2.65E+00
LINC00882	lncRNA	1.83E-04	3.02E-03	2.65E+00
ZNF195	protein_coding	6.52E-05	1.44E-03	2.66E+00
CWC27	protein_coding	1.46E-05	5.17E-04	2.66E+00
FAM184A	protein_coding	5.03E-04	5.96E-03	2.66E+00

ZMYM6	protein_coding	3.94E-04	5.08E-03	2.66E+00
SENP7	protein_coding	9.23E-05	1.88E-03	2.67E+00
SREK1	protein_coding	3.51E-06	1.81E-04	2.67E+00
SRRM2	protein_coding	2.94E-04	4.12E-03	2.67E+00
TET2	protein_coding	6.46E-06	2.84E-04	2.68E+00
HERC1	protein_coding	1.34E-05	4.86E-04	2.68E+00
NEMF	protein_coding	2.17E-06	1.27E-04	2.69E+00
LARS1	protein_coding	1.05E-07	1.14E-05	2.69E+00
MYO10	protein_coding	5.90E-04	6.58E-03	2.69E+00
ERC1	protein_coding	5.17E-05	1.22E-03	2.70E+00
NCBP3	protein_coding	8.54E-07	6.15E-05	2.71E+00
BEND7	protein_coding	1.08E-03	9.91E-03	2.71E+00
EIF2AK3	protein_coding	6.32E-05	1.41E-03	2.71E+00
SNHG5	lncRNA	2.11E-04	3.31E-03	2.72E+00
ZNF506	protein_coding	1.15E-04	2.20E-03	2.72E+00
KIAA1109	protein_coding	1.74E-05	5.82E-04	2.73E+00
ITSN2	protein_coding	6.72E-08	7.96E-06	2.73E+00
CDK11A	protein_coding	1.08E-03	9.94E-03	2.74E+00
VPS53	protein_coding	4.40E-04	5.45E-03	2.74E+00
CROCCP2	transcribed_unprocessed_pseudogene	5.18E-04	6.04E-03	2.75E+00
FRMD4A	protein_coding	2.48E-06	1.41E-04	2.76E+00
PSD3	protein_coding	2.25E-05	6.86E-04	2.76E+00
WDR59	protein_coding	3.06E-05	8.48E-04	2.76E+00
CWC25	protein_coding	1.74E-05	5.82E-04	2.76E+00
TEP1	protein_coding	8.79E-05	1.81E-03	2.77E+00
GUSBP1	transcribed_unprocessed_pseudogene	6.43E-04	6.97E-03	2.77E+00
KLF8	protein_coding	1.81E-04	2.99E-03	2.77E+00
GON4L	protein_coding	9.37E-05	1.90E-03	2.78E+00
RPAP2	protein_coding	5.09E-06	2.42E-04	2.78E+00
EHBP1	protein_coding	9.17E-04	8.86E-03	2.78E+00
SNRNP48	protein_coding	1.06E-04	2.08E-03	2.78E+00
PARP6	protein_coding	7.66E-06	3.22E-04	2.78E+00
HERC2P2	transcribed_unprocessed_pseudogene	3.00E-04	4.18E-03	2.79E+00
ZBTB40	protein_coding	4.22E-05	1.07E-03	2.79E+00
LYST	protein_coding	5.94E-05	1.36E-03	2.83E+00
TTL	protein_coding	3.37E-04	4.55E-03	2.83E+00

SMAD3	protein_coding	1.65E-04	2.80E-03	2.83E+00
AGO3	protein_coding	2.52E-05	7.34E-04	2.84E+00
DDX46	protein_coding	8.17E-05	1.72E-03	2.85E+00
MFSD14C	transcribed_unprocessed_pseudogene	1.90E-04	3.09E-03	2.86E+00
SNTB2	protein_coding	5.43E-04	6.25E-03	2.86E+00
DOCK7	protein_coding	9.89E-05	1.98E-03	2.86E+00
NBPF15	protein_coding	2.05E-04	3.23E-03	2.86E+00
NFATC4	protein_coding	1.42E-04	2.51E-03	2.86E+00
AFDN	protein_coding	9.18E-05	1.88E-03	2.87E+00
TTC21B	protein_coding	7.19E-05	1.56E-03	2.88E+00
PDE4D	protein_coding	1.65E-04	2.80E-03	2.89E+00
CEP162	protein_coding	7.60E-05	1.62E-03	2.89E+00
ZBTB20	protein_coding	1.88E-05	6.08E-04	2.90E+00
ARID4A	protein_coding	2.48E-08	3.55E-06	2.90E+00
JARID2	protein_coding	5.22E-04	6.07E-03	2.91E+00
NSD2	protein_coding	6.87E-04	7.33E-03	2.91E+00
BRCA1	protein_coding	8.12E-04	8.14E-03	2.91E+00
TAOK3	protein_coding	2.02E-05	6.37E-04	2.92E+00
ZNF532	protein_coding	5.94E-06	2.71E-04	2.93E+00
CEP63	protein_coding	5.56E-06	2.57E-04	2.94E+00
SHROOM4	protein_coding	4.16E-04	5.28E-03	2.94E+00
TRIO	protein_coding	2.91E-04	4.10E-03	2.95E+00
H19	lncRNA	8.27E-04	8.23E-03	2.95E+00
LRCH3	protein_coding	4.75E-04	5.74E-03	2.98E+00
ANKRD11	protein_coding	2.29E-04	3.48E-03	2.98E+00
THADA	protein_coding	4.60E-05	1.14E-03	2.98E+00
PDE8A	protein_coding	2.94E-04	4.12E-03	2.98E+00
CHD6	protein_coding	2.21E-06	1.29E-04	3.00E+00
NPIPB5	protein_coding	3.13E-05	8.64E-04	3.00E+00
PSMG4	protein_coding	2.70E-05	7.74E-04	3.00E+00
RBM6	protein_coding	2.48E-04	3.69E-03	3.01E+00
CCNL2	protein_coding	4.85E-14	9.24E-11	3.01E+00
MYSM1	protein_coding	1.06E-06	7.31E-05	3.02E+00
GSDMB	protein_coding	6.11E-06	2.75E-04	3.02E+00
FANCI	protein_coding	4.02E-04	5.16E-03	3.03E+00
ZFP90	protein_coding	4.98E-04	5.91E-03	3.04E+00
ZC3H8	protein_coding	1.11E-04	2.14E-03	3.05E+00
TNK2	protein_coding	4.07E-04	5.20E-03	3.05E+00

LRRFIP1	protein_coding	1.53E-05	5.30E-04	3.06E+00
ZNF862	protein_coding	7.30E-04	7.63E-03	3.07E+00
KIAA0753	protein_coding	8.79E-04	8.57E-03	3.07E+00
ATP9B	protein_coding	1.62E-04	2.76E-03	3.08E+00
MZF1	protein_coding	1.14E-04	2.17E-03	3.08E+00
ANKRD26	protein_coding	1.98E-05	6.30E-04	3.08E+00
HACE1	protein_coding	1.99E-04	3.17E-03	3.08E+00
BDP1	protein_coding	1.98E-04	3.16E-03	3.09E+00
HIP1	protein_coding	4.58E-04	5.61E-03	3.09E+00
ATM	protein_coding	2.04E-05	6.41E-04	3.11E+00
DDX10	protein_coding	5.17E-06	2.44E-04	3.11E+00
PHF14	protein_coding	2.16E-04	3.35E-03	3.11E+00
RBL2	protein_coding	1.18E-07	1.23E-05	3.11E+00
GARS1-DT	lncRNA	3.40E-05	9.19E-04	3.12E+00
ZNF440	protein_coding	3.81E-04	4.97E-03	3.14E+00
SSH1	protein_coding	1.41E-04	2.50E-03	3.15E+00
TBCK	protein_coding	2.27E-06	1.32E-04	3.15E+00
ZNF44	protein_coding	2.36E-05	7.06E-04	3.17E+00
MYO5A	protein_coding	2.60E-05	7.48E-04	3.17E+00
UBA5	protein_coding	3.93E-04	5.07E-03	3.17E+00
WHAMM	protein_coding	5.41E-04	6.23E-03	3.18E+00
ZMIZ2	protein_coding	1.86E-04	3.04E-03	3.18E+00
PHF21A	protein_coding	3.91E-05	1.02E-03	3.18E+00
CAPRIN2	protein_coding	8.75E-04	8.55E-03	3.18E+00
AFG3L1P	transcribed_unitary_pseudogene	2.53E-07	2.33E-05	3.19E+00
RNASEH2B	protein_coding	1.57E-05	5.42E-04	3.19E+00
LRP6	protein_coding	1.90E-04	3.08E-03	3.19E+00
CEP170	protein_coding	4.62E-06	2.24E-04	3.20E+00
PRPF38B	protein_coding	1.98E-05	6.31E-04	3.20E+00
HERC2P9	transcribed_unprocessed_pseudogene	5.12E-06	2.42E-04	3.21E+00
LINC-PINT	lncRNA	3.34E-06	1.75E-04	3.21E+00
POLR1B	protein_coding	7.45E-04	7.73E-03	3.22E+00
KIAA0586	protein_coding	3.11E-04	4.27E-03	3.22E+00
CSPP1	protein_coding	2.33E-08	3.39E-06	3.23E+00
ZNF789	protein_coding	5.06E-05	1.21E-03	3.23E+00
NAV2	protein_coding	1.18E-06	7.88E-05	3.26E+00
NPIPB13	protein_coding	1.72E-05	5.78E-04	3.27E+00

CPLANE1	protein_coding	7.59E-07	5.65E-05	3.27E+00
DGKH	protein_coding	1.03E-04	2.04E-03	3.28E+00
MAGI1	protein_coding	2.41E-05	7.13E-04	3.28E+00
ARHGEF7	protein_coding	6.46E-09	1.17E-06	3.30E+00
CENPJ	protein_coding	7.33E-06	3.13E-04	3.31E+00
INPP5E	protein_coding	7.13E-04	7.53E-03	3.32E+00
DNAAF9	protein_coding	1.99E-04	3.17E-03	3.32E+00
TEPSIN	protein_coding	2.62E-04	3.84E-03	3.33E+00
POLR1A	protein_coding	1.93E-04	3.10E-03	3.34E+00
POLR2J3	protein_coding	2.05E-04	3.23E-03	3.35E+00
ZNF333	protein_coding	1.09E-04	2.11E-03	3.36E+00
SFMBT2	protein_coding	9.52E-04	9.12E-03	3.36E+00
SNX33	protein_coding	7.20E-04	7.56E-03	3.36E+00
SHROOM1	protein_coding	9.89E-04	9.32E-03	3.37E+00
TCHP	protein_coding	6.44E-05	1.43E-03	3.38E+00
ZNF514	protein_coding	8.42E-04	8.35E-03	3.38E+00
DDX60L	protein_coding	7.28E-07	5.47E-05	3.38E+00
MLXIP	protein_coding	4.27E-04	5.37E-03	3.38E+00
EVL	protein_coding	1.02E-05	3.93E-04	3.39E+00
ME2	protein_coding	7.18E-04	7.55E-03	3.39E+00
SEC31B	protein_coding	4.61E-04	5.63E-03	3.39E+00
RESF1	protein_coding	2.43E-06	1.38E-04	3.40E+00
SHPRH	protein_coding	5.26E-08	6.60E-06	3.41E+00
DST	protein_coding	3.64E-11	1.78E-08	3.41E+00
SGSM2	protein_coding	3.91E-06	1.98E-04	3.42E+00
SH3BP5-AS1	lncRNA	7.79E-04	7.96E-03	3.43E+00
KMT2A	protein_coding	1.51E-06	9.70E-05	3.44E+00
ANKS6	protein_coding	3.19E-04	4.35E-03	3.44E+00
PABPC1L	protein_coding	2.93E-05	8.23E-04	3.44E+00
ZNF37A	protein_coding	5.97E-06	2.71E-04	3.45E+00
FHIP1B	protein_coding	1.59E-05	5.45E-04	3.46E+00
DLEU1	lncRNA	4.96E-04	5.90E-03	3.46E+00
ZNF266	protein_coding	4.19E-07	3.52E-05	3.47E+00
SPATA5	protein_coding	4.73E-04	5.72E-03	3.47E+00
ARHGAP35	protein_coding	2.79E-04	3.98E-03	3.48E+00
KSR1	protein_coding	5.72E-05	1.33E-03	3.48E+00
ATXN3	protein_coding	4.91E-05	1.18E-03	3.48E+00

ATP6AP1L	transcribed_unitary_pseudogene	9.23E-04	8.90E-03	3.48E+00
KANTR	protein_coding	5.42E-04	6.24E-03	3.48E+00
MEIS2	protein_coding	2.00E-05	6.33E-04	3.49E+00
AMY2B	protein_coding	3.46E-04	4.64E-03	3.49E+00
ITSN1	protein_coding	3.46E-10	1.25E-07	3.49E+00
REV3L	protein_coding	1.15E-07	1.21E-05	3.51E+00
KRBA2	protein_coding	9.16E-04	8.86E-03	3.54E+00
SYNPO2	protein_coding	4.11E-04	5.24E-03	3.54E+00
HERC2P3	transcribed_unprocessed_pseudogene	5.61E-05	1.31E-03	3.55E+00
VPS13B	protein_coding	8.59E-07	6.16E-05	3.55E+00
NOTCH3	protein_coding	9.00E-04	8.74E-03	3.55E+00
EFCAB13	protein_coding	1.06E-03	9.78E-03	3.56E+00
ODF2L	protein_coding	2.24E-05	6.86E-04	3.56E+00
MDN1	protein_coding	6.95E-10	2.21E-07	3.56E+00
ELP1	protein_coding	3.10E-04	4.27E-03	3.57E+00
ARHGEF40	protein_coding	4.32E-04	5.41E-03	3.58E+00
CCDC14	protein_coding	2.87E-09	6.35E-07	3.58E+00
TANC1	protein_coding	6.91E-05	1.50E-03	3.59E+00
TTLL3	protein_coding	1.14E-04	2.18E-03	3.59E+00
CDK17	protein_coding	2.15E-08	3.16E-06	3.60E+00
MST1	protein_coding	7.50E-04	7.76E-03	3.60E+00
SAMD9L	protein_coding	2.22E-04	3.42E-03	3.60E+00
SESTD1	protein_coding	1.28E-04	2.35E-03	3.61E+00
SNHG17	lncRNA	3.99E-05	1.03E-03	3.61E+00
CCDC88A	protein_coding	5.32E-07	4.28E-05	3.61E+00
FGD5	protein_coding	7.93E-04	8.04E-03	3.62E+00
PCLO	protein_coding	4.91E-05	1.18E-03	3.63E+00
PEX26	protein_coding	4.40E-05	1.11E-03	3.64E+00
MYO18A	protein_coding	4.27E-06	2.10E-04	3.65E+00
FOXF1	protein_coding	7.13E-04	7.53E-03	3.65E+00
PRKD3	protein_coding	3.02E-06	1.62E-04	3.65E+00
DDX11	protein_coding	4.40E-04	5.45E-03	3.66E+00
AHI1	protein_coding	1.42E-19	9.02E-16	3.67E+00
UPF3A	protein_coding	1.14E-11	7.01E-09	3.67E+00
CEP112	protein_coding	8.55E-05	1.77E-03	3.68E+00
NLRC5	protein_coding	9.56E-04	9.14E-03	3.68E+00

SEPTIN7P2	transcribed_unprocessed_pseudogene	5.46E-08	6.77E-06	3.71E+00
DDHD1	protein_coding	1.11E-05	4.21E-04	3.73E+00
TBC1D4	protein_coding	1.18E-10	5.12E-08	3.74E+00
ZNF26	protein_coding	4.64E-05	1.14E-03	3.74E+00
NPHP3	protein_coding	5.20E-15	1.10E-11	3.74E+00
RPGR	protein_coding	2.73E-05	7.80E-04	3.75E+00
ZNF337	protein_coding	4.46E-05	1.12E-03	3.76E+00
LRRC37A2	protein_coding	2.88E-05	8.12E-04	3.76E+00
ALS2	protein_coding	1.52E-06	9.72E-05	3.78E+00
TRERF1	protein_coding	5.81E-04	6.51E-03	3.79E+00
MAGI2-AS3	lncRNA	7.42E-04	7.72E-03	3.79E+00
PDE3B	protein_coding	9.39E-05	1.90E-03	3.79E+00
PAK4	protein_coding	4.30E-04	5.39E-03	3.79E+00
CNTLN	protein_coding	2.70E-04	3.91E-03	3.80E+00
FAM228B	protein_coding	1.51E-06	9.72E-05	3.84E+00
D2HGDH	protein_coding	1.91E-04	3.09E-03	3.84E+00
FAM111A	protein_coding	1.11E-07	1.18E-05	3.85E+00
DOCK5	protein_coding	7.34E-06	3.13E-04	3.86E+00
FAT4	protein_coding	8.79E-04	8.57E-03	3.86E+00
NKTR	protein_coding	6.43E-09	1.17E-06	3.86E+00
RUSC2	protein_coding	1.28E-04	2.34E-03	3.87E+00
PPARA	protein_coding	9.92E-04	9.34E-03	3.89E+00
EPB41L4A	protein_coding	2.72E-04	3.93E-03	3.90E+00
ARAP3	protein_coding	2.39E-05	7.09E-04	3.90E+00
TTLL5	protein_coding	3.05E-05	8.48E-04	3.90E+00
LINC00472	lncRNA	3.03E-05	8.45E-04	3.91E+00
ZNF850	protein_coding	9.36E-05	1.90E-03	3.91E+00
WFDC1	protein_coding	5.67E-04	6.41E-03	3.92E+00
GPAM	protein_coding	7.81E-04	7.96E-03	3.93E+00
ICA1L	protein_coding	2.90E-04	4.10E-03	3.94E+00
ZNF577	protein_coding	6.09E-05	1.38E-03	3.94E+00
SYNE3	protein_coding	5.30E-05	1.25E-03	3.94E+00
FANCM	protein_coding	3.47E-06	1.80E-04	3.96E+00
ALMS1	protein_coding	1.44E-07	1.46E-05	3.96E+00
SLC7A11	protein_coding	7.83E-04	7.97E-03	3.99E+00
WDR27	protein_coding	4.93E-05	1.19E-03	3.99E+00
POLG2	protein_coding	3.39E-04	4.57E-03	4.00E+00
DLEU2	lncRNA	1.63E-04	2.77E-03	4.01E+00

ZNF783	protein_coding	5.40E-05	1.26E-03	4.01E+00
PRICKLE2	protein_coding	2.53E-04	3.75E-03	4.01E+00
CSAD	protein_coding	4.03E-06	2.02E-04	4.02E+00
NLN	protein_coding	1.02E-03	9.54E-03	4.05E+00
FGFR1	protein_coding	5.01E-06	2.38E-04	4.07E+00
MMS22L	protein_coding	3.06E-04	4.23E-03	4.07E+00
CRELD2	protein_coding	3.44E-05	9.22E-04	4.08E+00
SP110	protein_coding	2.65E-07	2.43E-05	4.08E+00
FAM241A	protein_coding	3.75E-05	9.78E-04	4.10E+00
ERCC6L2-AS1	lncRNA	5.13E-04	6.02E-03	4.11E+00
NPTN-IT1	lncRNA	4.68E-04	5.68E-03	4.14E+00
GCOM1	protein_coding	9.87E-04	9.31E-03	4.15E+00
GK5	protein_coding	1.52E-05	5.30E-04	4.16E+00
ARMC9	protein_coding	4.13E-04	5.25E-03	4.16E+00
SLC4A7	protein_coding	1.17E-04	2.22E-03	4.16E+00
GASK1A	protein_coding	1.45E-06	9.46E-05	4.16E+00
SLC1A2	protein_coding	1.11E-04	2.14E-03	4.18E+00
PEAK1	protein_coding	1.09E-08	1.80E-06	4.18E+00
PPFIBP2	protein_coding	2.07E-04	3.25E-03	4.19E+00
GTF2IP12	transcribed_unprocessed_pseudogene	3.58E-04	4.75E-03	4.20E+00
PCSK6	protein_coding	3.58E-04	4.75E-03	4.20E+00
TTC28	protein_coding	7.41E-05	1.59E-03	4.21E+00
DMPK	protein_coding	2.82E-04	4.00E-03	4.22E+00
CEP120	protein_coding	7.06E-04	7.49E-03	4.24E+00
TLCD2	protein_coding	4.02E-04	5.16E-03	4.27E+00
EMC3-AS1	transcribed_unprocessed_pseudogene	1.00E-03	9.41E-03	4.28E+00
NFIA	protein_coding	9.65E-10	2.75E-07	4.30E+00
PLEKHG2	protein_coding	1.00E-04	1.99E-03	4.32E+00
STK36	protein_coding	6.34E-06	2.83E-04	4.33E+00
SRSF11	protein_coding	2.89E-08	4.09E-06	4.33E+00
ZHX3	protein_coding	2.22E-04	3.42E-03	4.35E+00
PHTF1	protein_coding	3.99E-05	1.03E-03	4.36E+00
ZNF844	protein_coding	3.05E-04	4.23E-03	4.36E+00
PALD1	protein_coding	9.83E-04	9.28E-03	4.37E+00
CEP250	protein_coding	1.77E-05	5.88E-04	4.41E+00
LAT	protein_coding	1.69E-05	5.71E-04	4.41E+00

TRPS1	protein_coding	2.81E-04	4.00E-03	4.41E+00
ADAL	protein_coding	8.65E-04	8.50E-03	4.42E+00
FLYWCH1	protein_coding	2.19E-05	6.76E-04	4.42E+00
GRB10	protein_coding	7.26E-07	5.47E-05	4.43E+00
KAT2A	protein_coding	2.22E-11	1.15E-08	4.43E+00
ARMCX4	protein_coding	6.35E-06	2.83E-04	4.43E+00
OLMALINC	lncRNA	5.92E-04	6.60E-03	4.44E+00
CDC42BPA	protein_coding	4.22E-09	8.55E-07	4.49E+00
BICD1	protein_coding	1.26E-05	4.66E-04	4.51E+00
HPS4	protein_coding	5.72E-08	6.99E-06	4.52E+00
TNRC6C	protein_coding	4.14E-04	5.26E-03	4.53E+00
AP4B1	protein_coding	5.98E-05	1.37E-03	4.54E+00
GOT1-DT	lncRNA	3.90E-04	5.05E-03	4.54E+00
CARMN	lncRNA	1.15E-05	4.33E-04	4.55E+00
SFI1	protein_coding	1.89E-04	3.08E-03	4.55E+00
IFT81	protein_coding	3.78E-04	4.94E-03	4.55E+00
ARHGEF15	protein_coding	1.90E-04	3.08E-03	4.56E+00
BMS1P23	transcribed_unprocessed_pseudogene	2.48E-04	3.69E-03	4.57E+00
CCDC144CP	transcribed_processed_pseudogene	5.38E-05	1.26E-03	4.57E+00
RNF152	protein_coding	5.07E-04	5.99E-03	4.58E+00
CC2D2A	protein_coding	1.38E-05	4.95E-04	4.59E+00
MIR193BHG	lncRNA	9.76E-04	9.25E-03	4.64E+00
DNHD1	protein_coding	1.67E-04	2.82E-03	4.64E+00
ZNF704	protein_coding	1.38E-08	2.22E-06	4.66E+00
GPT2	protein_coding	1.56E-04	2.69E-03	4.67E+00
TNFRSF25	protein_coding	5.30E-07	4.28E-05	4.68E+00
CEP290	protein_coding	1.99E-12	1.52E-09	4.68E+00
THAP9	protein_coding	6.83E-04	7.30E-03	4.70E+00
NCKAP5L	protein_coding	2.23E-04	3.43E-03	4.71E+00
FADS3	protein_coding	5.45E-06	2.55E-04	4.71E+00
GFOD1	protein_coding	7.64E-06	3.22E-04	4.72E+00
AASS	protein_coding	3.32E-08	4.63E-06	4.72E+00
COPG2IT1	lncRNA	2.68E-04	3.88E-03	4.72E+00
ZNF470	protein_coding	6.69E-04	7.20E-03	4.73E+00
WBP2NL	protein_coding	5.05E-04	5.97E-03	4.74E+00
TMEM154	protein_coding	1.59E-05	5.44E-04	4.74E+00

AMT	protein_coding	2.25E-05	6.88E-04	4.75E+00
TRIML2	protein_coding	2.16E-06	1.27E-04	4.75E+00
EPN1	protein_coding	2.86E-05	8.08E-04	4.76E+00
AKNA	protein_coding	7.88E-05	1.67E-03	4.76E+00
SH3PXD2B	protein_coding	1.08E-03	9.89E-03	4.77E+00
ITPR1	protein_coding	1.69E-08	2.61E-06	4.82E+00
TTC39B	protein_coding	1.10E-05	4.18E-04	4.82E+00
FAM193A	protein_coding	3.71E-07	3.22E-05	4.86E+00
PLCE1	protein_coding	8.70E-06	3.49E-04	4.88E+00
LINC02055	lncRNA	5.20E-04	6.06E-03	4.88E+00
PALB2	protein_coding	5.17E-04	6.04E-03	4.89E+00
SLX1B-SULT1A4	lncRNA	4.01E-04	5.15E-03	4.90E+00
TYRO3	protein_coding	2.73E-04	3.93E-03	4.91E+00
NOPCHAP1	protein_coding	1.27E-04	2.33E-03	4.92E+00
MICAL3	protein_coding	4.40E-04	5.45E-03	4.92E+00
PPP4R1L	transcribed_unprocessed_pseudogene	8.26E-05	1.73E-03	4.92E+00
FHOD1	protein_coding	3.95E-06	1.99E-04	4.93E+00
FSIP2	protein_coding	3.75E-04	4.92E-03	4.93E+00
MAPK8IP3	protein_coding	2.47E-07	2.28E-05	4.95E+00
SLC16A1-AS1	lncRNA	8.28E-04	8.23E-03	4.95E+00
NRBP2	protein_coding	7.94E-07	5.82E-05	4.95E+00
GOLGA8B	protein_coding	4.38E-09	8.73E-07	4.96E+00
PDE8B	protein_coding	5.28E-04	6.12E-03	4.96E+00
PDCL3P4	transcribed_processed_pseudogene	1.18E-04	2.22E-03	4.96E+00
CCDC144B	transcribed_unprocessed_pseudogene	2.86E-18	1.36E-14	4.97E+00
WDPCP	protein_coding	6.90E-05	1.50E-03	4.97E+00
SYNE1	protein_coding	6.39E-06	2.83E-04	4.98E+00
BDNF	protein_coding	3.05E-04	4.23E-03	5.00E+00
USP32P3	transcribed_unprocessed_pseudogene	1.19E-04	2.24E-03	5.01E+00
SPECC1	protein_coding	2.46E-05	7.19E-04	5.02E+00
SRGAP1	protein_coding	3.77E-10	1.33E-07	5.02E+00
PCBD2	protein_coding	1.98E-04	3.16E-03	5.02E+00
NPDC1	protein_coding	3.06E-05	8.48E-04	5.03E+00
ALS2CL	protein_coding	2.80E-08	3.98E-06	5.03E+00

FLVCR1	protein_coding	3.14E-05	8.66E-04	5.04E+00
ADAMTS18	protein_coding	6.77E-04	7.26E-03	5.05E+00
ZNF331	protein_coding	5.86E-07	4.60E-05	5.05E+00
PLCB4	protein_coding	9.90E-08	1.08E-05	5.06E+00
CCDC150	protein_coding	9.92E-05	1.98E-03	5.07E+00
RRAD	protein_coding	4.42E-04	5.45E-03	5.07E+00
PCSK5	protein_coding	7.68E-04	7.88E-03	5.09E+00
SCAND2P	transcribed_unprocessed_pseudogene	9.94E-04	9.35E-03	5.10E+00
USP32P1	transcribed_unprocessed_pseudogene	2.20E-05	6.77E-04	5.10E+00
ANKS3	protein_coding	3.55E-05	9.44E-04	5.11E+00
RNF213-AS1	lncRNA	2.47E-04	3.69E-03	5.14E+00
GUSBP11	lncRNA	1.04E-05	3.99E-04	5.14E+00
LGI4	protein_coding	7.52E-04	7.76E-03	5.15E+00
KLF7	protein_coding	3.55E-05	9.44E-04	5.15E+00
HMCN1	protein_coding	9.12E-06	3.63E-04	5.16E+00
AGAP6	protein_coding	1.67E-12	1.39E-09	5.18E+00
ZNF445	protein_coding	2.36E-05	7.06E-04	5.18E+00
MYOM1	protein_coding	6.22E-04	6.82E-03	5.20E+00
NR2F2-AS1	lncRNA	6.37E-08	7.64E-06	5.21E+00
PPIEL	transcribed_unprocessed_pseudogene	5.18E-05	1.22E-03	5.25E+00
SLC35E3	protein_coding	1.43E-04	2.52E-03	5.27E+00
KIAA0895L	protein_coding	3.00E-06	1.62E-04	5.28E+00
RYR2	protein_coding	1.50E-04	2.62E-03	5.28E+00
UPF3AP2	transcribed_processed_pseudogene	3.24E-04	4.40E-03	5.28E+00
PGR	protein_coding	7.66E-04	7.87E-03	5.28E+00
NPIPA1	protein_coding	1.52E-05	5.30E-04	5.28E+00
KCNK6	protein_coding	6.19E-04	6.79E-03	5.30E+00
ADCY9	protein_coding	5.11E-04	6.01E-03	5.31E+00
ASAP3	protein_coding	1.04E-04	2.05E-03	5.31E+00
HSF4	protein_coding	4.02E-05	1.03E-03	5.31E+00
IFFO1	protein_coding	3.86E-04	5.01E-03	5.33E+00
LINC01415	lncRNA	1.90E-04	3.08E-03	5.33E+00
KANK3	protein_coding	2.18E-05	6.76E-04	5.34E+00
NDUFA9P1	processed_pseudogene	3.67E-04	4.85E-03	5.35E+00

JDP2	protein_coding	3.18E-04	4.35E-03	5.39E+00
ANKRD36B	protein_coding	2.07E-10	8.22E-08	5.41E+00
LUC7L3	protein_coding	1.23E-11	7.32E-09	5.42E+00
WDR19	protein_coding	1.54E-06	9.81E-05	5.43E+00
DIO3OS	lncRNA	4.79E-04	5.76E-03	5.43E+00
PTH1R	protein_coding	7.23E-04	7.58E-03	5.45E+00
DUXAP8	lncRNA	1.02E-03	9.51E-03	5.48E+00
MTND1P23	unprocessed_pseudogene	1.06E-03	9.81E-03	5.48E+00
SOX6	protein_coding	7.29E-10	2.21E-07	5.50E+00
TAL1	protein_coding	2.40E-05	7.12E-04	5.52E+00
GHRLOS	lncRNA	2.65E-04	3.86E-03	5.54E+00
TSEN2	protein_coding	5.10E-04	6.01E-03	5.55E+00
SNHG7	lncRNA	4.40E-06	2.15E-04	5.56E+00
DYNC2H1	protein_coding	2.81E-07	2.55E-05	5.56E+00
LLGL2	protein_coding	6.55E-05	1.45E-03	5.57E+00
GOLGA6L9	protein_coding	3.23E-09	7.00E-07	5.57E+00
ST3GAL5	protein_coding	8.56E-04	8.44E-03	5.58E+00
SLC27A5	protein_coding	2.51E-04	3.72E-03	5.63E+00
SGMS1-AS1	lncRNA	5.94E-04	6.61E-03	5.66E+00
RPGRIP1L	protein_coding	3.32E-04	4.48E-03	5.67E+00
LINC01881	transcribed_unprocessed_pseudogene	1.56E-06	9.91E-05	5.68E+00
SCAI	protein_coding	3.56E-04	4.73E-03	5.68E+00
PGGHG	protein_coding	4.63E-04	5.64E-03	5.68E+00
COL4A5	protein_coding	4.73E-04	5.72E-03	5.69E+00
TRDMT1	protein_coding	7.01E-05	1.52E-03	5.70E+00
MLYCD	protein_coding	3.03E-04	4.20E-03	5.71E+00
CACNA2D1	protein_coding	2.61E-06	1.47E-04	5.73E+00
DONSON	protein_coding	4.12E-06	2.05E-04	5.73E+00
CAPN3	protein_coding	3.45E-09	7.39E-07	5.74E+00
TRPM4	protein_coding	4.56E-06	2.21E-04	5.76E+00
RTTN	protein_coding	1.28E-05	4.69E-04	5.77E+00
GMDS-DT	lncRNA	2.73E-04	3.93E-03	5.77E+00
SLC12A2-DT	lncRNA	3.91E-04	5.06E-03	5.80E+00
PHLDB1	protein_coding	4.78E-04	5.76E-03	5.80E+00
AGAP9	protein_coding	6.80E-04	7.28E-03	5.82E+00
LSM11	protein_coding	2.99E-04	4.17E-03	5.85E+00

ADCY4	protein_coding	6.08E-04	6.72E-03	5.86E+00
ADARB1	protein_coding	1.56E-06	9.91E-05	5.88E+00
LINC01725	lncRNA	2.80E-05	7.95E-04	5.91E+00
C21orf62-AS1	lncRNA	2.09E-04	3.28E-03	5.93E+00
SPTBN2	protein_coding	9.97E-04	9.36E-03	5.95E+00
SPINK5	protein_coding	3.82E-07	3.27E-05	5.95E+00
TONSL	protein_coding	4.79E-04	5.76E-03	5.95E+00
TRAF5	protein_coding	1.87E-06	1.13E-04	5.98E+00
PDC-AS1	lncRNA	1.07E-03	9.86E-03	5.99E+00
CCM2L	protein_coding	3.33E-05	9.06E-04	5.99E+00
ZNF780B	protein_coding	6.84E-06	2.96E-04	6.01E+00
NCOR2	protein_coding	1.51E-05	5.30E-04	6.03E+00
MAPT	protein_coding	1.25E-04	2.31E-03	6.04E+00
MEG8	lncRNA	1.67E-07	1.64E-05	6.04E+00
LTB4R	protein_coding	3.59E-05	9.51E-04	6.05E+00
LARP6	protein_coding	4.51E-04	5.53E-03	6.06E+00
TTN	protein_coding	7.76E-20	7.40E-16	6.08E+00
GARIN1A	protein_coding	7.70E-04	7.89E-03	6.13E+00
NRXN3	protein_coding	9.56E-06	3.75E-04	6.13E+00
PPARGC1B	protein_coding	4.73E-05	1.16E-03	6.14E+00
ANKRD36C	protein_coding	8.29E-06	3.39E-04	6.14E+00
EBF3	protein_coding	4.98E-05	1.20E-03	6.16E+00
SLC25A29	protein_coding	5.14E-06	2.43E-04	6.16E+00
MYEF2	protein_coding	5.57E-04	6.34E-03	6.16E+00
SLC25A25-AS1	lncRNA	6.31E-05	1.41E-03	6.25E+00
GVQW3	protein_coding	1.06E-04	2.07E-03	6.26E+00
ESCO2	protein_coding	4.35E-04	5.43E-03	6.27E+00
NEGR1	protein_coding	1.11E-04	2.14E-03	6.29E+00
VIPR2	protein_coding	9.57E-05	1.93E-03	6.31E+00
COL12A1	protein_coding	9.67E-04	9.19E-03	6.34E+00
POU6F1	protein_coding	1.75E-05	5.84E-04	6.34E+00
LINC01588	lncRNA	1.58E-04	2.71E-03	6.38E+00
KLF3-AS1	lncRNA	4.93E-04	5.89E-03	6.40E+00
RASGRF2	protein_coding	3.80E-07	3.27E-05	6.40E+00
CDKN2B-AS1	lncRNA	1.03E-03	9.57E-03	6.41E+00
PKP4	protein_coding	5.06E-07	4.14E-05	6.43E+00

EPHA4	protein_coding	4.07E-04	5.21E-03	6.43E+00
LINC01205	lncRNA	4.01E-05	1.03E-03	6.44E+00
RBM25	protein_coding	1.62E-09	4.06E-07	6.45E+00
CELF6	protein_coding	1.91E-04	3.09E-03	6.45E+00
STARD9	protein_coding	1.54E-04	2.67E-03	6.46E+00
LRRFIP1P1	processed_pseudogene	1.23E-08	2.01E-06	6.47E+00
COLQ	protein_coding	8.02E-04	8.09E-03	6.51E+00
SLFN11	protein_coding	9.59E-06	3.76E-04	6.51E+00
TTC21A	protein_coding	8.76E-07	6.23E-05	6.53E+00
PKD2L2-DT	lncRNA	6.40E-05	1.42E-03	6.54E+00
GPRC5A	protein_coding	4.87E-04	5.84E-03	6.54E+00
LINC00645	lncRNA	3.98E-04	5.12E-03	6.54E+00
FEM1A	protein_coding	2.55E-04	3.76E-03	6.55E+00
ODAD4	protein_coding	1.77E-04	2.94E-03	6.59E+00
CFAP251	protein_coding	5.90E-04	6.58E-03	6.61E+00
COL27A1	protein_coding	6.40E-09	1.17E-06	6.62E+00
FRMD5	protein_coding	1.06E-03	9.81E-03	6.62E+00
LOXL1-AS1	lncRNA	5.29E-04	6.13E-03	6.63E+00
ZNF793	protein_coding	5.87E-05	1.35E-03	6.65E+00
HIVEP3	protein_coding	2.28E-04	3.48E-03	6.67E+00
RIMS3	protein_coding	2.27E-04	3.47E-03	6.70E+00
NTRK2	protein_coding	3.20E-04	4.36E-03	6.76E+00
MTND5P11	processed_pseudogene	9.65E-04	9.18E-03	6.78E+00
DOCK6	protein_coding	7.32E-05	1.58E-03	6.79E+00
STX18-AS1	lncRNA	1.01E-05	3.91E-04	6.83E+00
ADCY2	protein_coding	2.21E-04	3.41E-03	6.84E+00
EML6	protein_coding	1.27E-04	2.33E-03	6.86E+00
DCLK2	protein_coding	1.08E-04	2.10E-03	6.87E+00
TBX19	protein_coding	1.41E-04	2.50E-03	6.88E+00
CDRT4	protein_coding	1.44E-04	2.53E-03	6.88E+00
KIAA0825	protein_coding	6.05E-04	6.70E-03	6.89E+00
ZNF114	protein_coding	4.15E-04	5.27E-03	6.90E+00
ANKRD20A17P	unprocessed_pseudogene	4.26E-07	3.54E-05	6.92E+00
NLRP1	protein_coding	4.51E-06	2.19E-04	6.92E+00
PVT1	lncRNA	1.50E-06	9.68E-05	6.94E+00
CIT	protein_coding	1.31E-04	2.37E-03	6.94E+00

JPH2	protein_coding	7.19E-07	5.44E-05	6.94E+00
MAGI2	protein_coding	2.90E-06	1.58E-04	6.95E+00
ZNF334	protein_coding	5.09E-08	6.47E-06	6.97E+00
LRRC8B	protein_coding	6.43E-05	1.43E-03	6.98E+00
MEGF6	protein_coding	5.11E-05	1.22E-03	6.99E+00
SACS	protein_coding	6.03E-05	1.37E-03	6.99E+00
DUXAP10	transcribed_processed_pseudogene	6.06E-05	1.38E-03	7.07E+00
HULC	lncRNA	2.04E-05	6.41E-04	7.08E+00
WTIP	protein_coding	3.64E-04	4.81E-03	7.09E+00
HSP90AB4P	processed_pseudogene	1.27E-05	4.67E-04	7.09E+00
LRRC36	protein_coding	4.56E-04	5.58E-03	7.11E+00
ADA	protein_coding	1.27E-04	2.34E-03	7.12E+00
SYT15B	protein_coding	2.37E-05	7.09E-04	7.15E+00
GTF2IRD1	protein_coding	1.74E-04	2.91E-03	7.15E+00
MEG3	lncRNA	8.94E-07	6.34E-05	7.15E+00
KATNAL2	protein_coding	7.54E-04	7.78E-03	7.16E+00
PROSER3	protein_coding	4.89E-05	1.18E-03	7.16E+00
ZMAT1	protein_coding	1.71E-11	9.04E-09	7.19E+00
SLC25A15	protein_coding	8.96E-04	8.71E-03	7.20E+00
MIPOL1	protein_coding	5.63E-04	6.37E-03	7.20E+00
ANKRD36	protein_coding	4.98E-08	6.37E-06	7.20E+00
HOXB3	protein_coding	3.36E-04	4.53E-03	7.23E+00
CNKSR3	protein_coding	9.84E-06	3.84E-04	7.23E+00
SLC23A3	protein_coding	3.10E-04	4.27E-03	7.30E+00
SRGAP3	protein_coding	7.69E-04	7.88E-03	7.30E+00
SGIP1	protein_coding	5.75E-04	6.47E-03	7.31E+00
SYCP2	protein_coding	2.01E-09	4.92E-07	7.33E+00
ARHGEF10	protein_coding	2.94E-07	2.64E-05	7.34E+00
MMP16	protein_coding	1.62E-04	2.76E-03	7.40E+00
ZNF608	protein_coding	4.97E-04	5.91E-03	7.42E+00
DYNC2I1	protein_coding	1.57E-15	4.27E-12	7.43E+00
EYA4	protein_coding	1.13E-04	2.16E-03	7.45E+00
ADAMTS15	protein_coding	4.78E-05	1.16E-03	7.46E+00
COL18A1	protein_coding	4.47E-05	1.12E-03	7.46E+00
ESR1	protein_coding	5.96E-04	6.62E-03	7.47E+00
PPFIA4	protein_coding	3.13E-04	4.29E-03	7.49E+00
NOTCH4	protein_coding	3.40E-07	3.01E-05	7.49E+00

FLG-AS1	lncRNA	9.71E-04	9.22E-03	7.52E+00
PTCHD4	protein_coding	2.10E-05	6.54E-04	7.56E+00
HOXB-AS1	lncRNA	8.10E-04	8.14E-03	7.57E+00
ZBED3-AS1	lncRNA	7.26E-04	7.60E-03	7.59E+00
ABCA6	protein_coding	1.82E-04	3.00E-03	7.60E+00
RNF17	protein_coding	9.64E-04	9.17E-03	7.63E+00
PCNX2	protein_coding	1.34E-04	2.41E-03	7.63E+00
LINC01515	lncRNA	8.91E-06	3.55E-04	7.64E+00
ZNF37BP	transcribed_processed_pseudogene	5.86E-16	1.86E-12	7.65E+00
TMEM44	protein_coding	4.26E-06	2.10E-04	7.70E+00
ABCB9	protein_coding	3.06E-04	4.23E-03	7.73E+00
DNAH2	protein_coding	1.60E-04	2.74E-03	7.73E+00
ZNF483	protein_coding	3.74E-05	9.78E-04	7.73E+00
MIRLET7BHG	lncRNA	8.49E-04	8.41E-03	7.75E+00
LINC01134	lncRNA	1.03E-03	9.58E-03	7.82E+00
ICOSLG	protein_coding	2.44E-04	3.66E-03	7.83E+00
CCDC141	protein_coding	3.10E-04	4.27E-03	7.84E+00
MAPK12	protein_coding	3.44E-05	9.22E-04	7.85E+00
SPEG	protein_coding	2.64E-04	3.86E-03	7.87E+00
ZNF66	protein_coding	2.50E-05	7.29E-04	7.88E+00
KCTD15	protein_coding	6.91E-08	8.09E-06	7.88E+00
NSMF	protein_coding	6.45E-05	1.43E-03	7.90E+00
CEP164	protein_coding	6.32E-08	7.63E-06	7.91E+00
STOX2	protein_coding	1.59E-06	1.00E-04	7.94E+00
OR2G6	protein_coding	5.62E-04	6.36E-03	7.98E+00
MUC16	protein_coding	2.59E-05	7.48E-04	8.01E+00
ARL10	protein_coding	2.93E-05	8.23E-04	8.02E+00
CICP27	processed_pseudogene	5.82E-04	6.52E-03	8.03E+00
POU5F2	protein_coding	8.57E-06	3.45E-04	8.03E+00
PTOV1-AS1	lncRNA	4.90E-05	1.18E-03	8.06E+00
ZNF699	protein_coding	1.67E-04	2.82E-03	8.08E+00
NPIPB11	protein_coding	4.27E-05	1.08E-03	8.13E+00
LINC00578	lncRNA	4.43E-04	5.46E-03	8.13E+00
MRO	protein_coding	2.06E-04	3.24E-03	8.14E+00
GSTO2	protein_coding	3.50E-04	4.68E-03	8.16E+00
FAM88B	lncRNA	4.41E-04	5.45E-03	8.17E+00

ABCA9	protein_coding	7.35E-04	7.68E-03	8.18E+00
RNF217-AS1	lncRNA	4.64E-05	1.14E-03	8.19E+00
HERC2P4	transcribed_unprocessed_pseudogene	5.09E-04	6.00E-03	8.20E+00
FAM157C	lncRNA	2.88E-06	1.58E-04	8.20E+00
ANK2	protein_coding	7.80E-04	7.96E-03	8.21E+00
TDRD10	protein_coding	5.08E-04	5.99E-03	8.22E+00
XACT	lncRNA	2.08E-04	3.26E-03	8.23E+00
CIART	protein_coding	3.50E-04	4.68E-03	8.26E+00
PCAT1	lncRNA	6.13E-04	6.76E-03	8.36E+00
RIPOR3	protein_coding	2.73E-04	3.93E-03	8.37E+00
ATP8A2	protein_coding	1.14E-05	4.30E-04	8.40E+00
CHDH	protein_coding	7.81E-05	1.66E-03	8.46E+00
PPP1CB-DT	lncRNA	8.56E-04	8.44E-03	8.46E+00
PPM1F-AS1	lncRNA	2.01E-04	3.19E-03	8.53E+00
LINC01695	lncRNA	7.18E-04	7.55E-03	8.53E+00
TWSG1-DT	lncRNA	4.13E-04	5.25E-03	8.54E+00
WHAMMP1	transcribed_processed_pseudogene	1.65E-05	5.57E-04	8.54E+00
SEMA4F	protein_coding	9.08E-04	8.79E-03	8.55E+00
ZFAND2A-DT	lncRNA	4.53E-05	1.13E-03	8.57E+00
L2HGDH	protein_coding	9.54E-04	9.14E-03	8.60E+00
PPP5D1P	transcribed_unprocessed_pseudogene	1.57E-05	5.42E-04	8.62E+00
SAP30L-AS1	lncRNA	4.81E-05	1.17E-03	8.63E+00
VAC14-AS1	lncRNA	1.95E-05	6.24E-04	8.63E+00
LRRC34	protein_coding	7.81E-04	7.96E-03	8.64E+00
SOBP	protein_coding	5.97E-06	2.71E-04	8.71E+00
MTMR9LP	transcribed_unprocessed_pseudogene	6.18E-05	1.39E-03	8.74E+00
EIF1B-AS1	lncRNA	1.02E-03	9.55E-03	8.74E+00
ZNF660	protein_coding	6.89E-06	2.98E-04	8.74E+00
SCN1A-AS1	lncRNA	5.16E-04	6.03E-03	8.75E+00
GOLGA6L4	protein_coding	4.32E-05	1.09E-03	8.75E+00
LINC00506	lncRNA	3.37E-06	1.77E-04	8.76E+00
STXBP5-AS1	lncRNA	7.95E-04	8.05E-03	8.77E+00

HSPA12A	protein_coding	1.79E-05	5.89E-04	8.85E+00
GOLGA8A	protein_coding	1.79E-15	4.27E-12	8.86E+00
ZNF99	protein_coding	7.66E-04	7.87E-03	8.88E+00
ZNF284	protein_coding	1.63E-05	5.55E-04	8.90E+00
SLC9A3-AS1	lncRNA	2.26E-05	6.88E-04	8.99E+00
SATB2	protein_coding	5.62E-06	2.58E-04	8.99E+00
HELLPAR	lncRNA	1.10E-08	1.80E-06	9.00E+00
LINC01524	lncRNA	9.49E-04	9.11E-03	9.01E+00
PTPRG-AS1	lncRNA	3.01E-04	4.19E-03	9.01E+00
MYLK3	protein_coding	9.75E-04	9.24E-03	9.02E+00
SNHG14	lncRNA	3.58E-09	7.58E-07	9.03E+00
SMARCD3	protein_coding	4.35E-05	1.10E-03	9.05E+00
MIR646HG	lncRNA	9.54E-06	3.75E-04	9.06E+00
GRIP2	protein_coding	3.83E-05	9.99E-04	9.07E+00
KCNMB1	protein_coding	5.20E-04	6.06E-03	9.10E+00
KCNQ1OT1	lncRNA	2.20E-20	4.20E-16	9.14E+00
CROCCP3	transcribed_unprocessed_pseudogene	1.19E-06	7.94E-05	9.19E+00
NFASC	protein_coding	1.45E-07	1.47E-05	9.30E+00
APRG1	lncRNA	7.27E-05	1.57E-03	9.32E+00
CNTNAP3	protein_coding	3.60E-05	9.51E-04	9.33E+00
CCDC30	protein_coding	4.70E-08	6.09E-06	9.34E+00
BCO2	protein_coding	3.59E-07	3.13E-05	9.35E+00
EPIC1	lncRNA	2.39E-05	7.09E-04	9.35E+00
ZNF618	protein_coding	2.68E-05	7.69E-04	9.47E+00
CCDC144A	protein_coding	2.01E-07	1.92E-05	9.49E+00
GABBR1	protein_coding	3.68E-08	5.08E-06	9.51E+00
GAPDHP43	processed_pseudogene	7.18E-04	7.55E-03	9.51E+00
KIAA1671	protein_coding	1.31E-04	2.37E-03	9.62E+00
HLCS-AS1	lncRNA	2.82E-04	4.01E-03	9.62E+00
CAMK2A	protein_coding	3.74E-04	4.91E-03	9.63E+00
ANXA2R-AS1	lncRNA	1.42E-10	6.03E-08	9.64E+00
GRIK2	protein_coding	4.01E-06	2.02E-04	9.68E+00
TRPV3	protein_coding	2.82E-05	7.99E-04	9.69E+00
OR52B5P	transcribed_unprocessed_pseudogene	8.13E-04	8.14E-03	9.71E+00
TTLL7	protein_coding	3.29E-04	4.45E-03	9.76E+00

LINC01485	lncRNA	1.02E-03	9.55E-03	9.79E+00
UCKL1-AS1	lncRNA	3.32E-04	4.48E-03	9.80E+00
LINC01948	lncRNA	6.36E-04	6.93E-03	9.81E+00
BDH1	protein_coding	2.03E-06	1.21E-04	9.85E+00
CNTNAP1	protein_coding	8.28E-05	1.73E-03	9.90E+00
AKAP6	protein_coding	3.02E-05	8.45E-04	9.95E+00
NEPRO-AS1	lncRNA	8.24E-05	1.73E-03	9.97E+00
LRRC7	protein_coding	6.18E-04	6.79E-03	1.00E+01
LINC01285	lncRNA	3.00E-04	4.18E-03	1.00E+01
LINC02895	lncRNA	4.68E-06	2.26E-04	1.01E+01
DCST2	protein_coding	1.25E-04	2.31E-03	1.01E+01
ABCC9	protein_coding	2.63E-06	1.47E-04	1.01E+01
QRICH2	protein_coding	1.48E-04	2.58E-03	1.01E+01
ZFAND4	protein_coding	8.22E-04	8.21E-03	1.01E+01
L3MBTL2-AS1	lncRNA	2.93E-04	4.11E-03	1.01E+01
ATP8B2	protein_coding	3.04E-05	8.47E-04	1.01E+01
CRYGS	protein_coding	1.45E-04	2.54E-03	1.02E+01
CCDC168	protein_coding	6.10E-05	1.38E-03	1.02E+01
SPRY3	protein_coding	9.43E-06	3.72E-04	1.02E+01
PYHIN1	protein_coding	1.81E-04	2.99E-03	1.02E+01
LINC01226	lncRNA	6.34E-04	6.92E-03	1.03E+01
LINC02969	lncRNA	2.84E-06	1.57E-04	1.03E+01
POT1-AS1	lncRNA	2.29E-04	3.48E-03	1.03E+01
IDO2	protein_coding	9.57E-08	1.05E-05	1.03E+01
MEFV	protein_coding	9.56E-04	9.14E-03	1.03E+01
TSPOAP1	protein_coding	7.19E-04	7.55E-03	1.03E+01
KDM1B	protein_coding	1.88E-04	3.06E-03	1.03E+01
POTEF	protein_coding	1.86E-04	3.04E-03	1.03E+01
ALDH1L2	protein_coding	5.87E-04	6.56E-03	1.03E+01
CYP2E1	protein_coding	3.55E-06	1.83E-04	1.03E+01
BMS1P1	transcribed_unprocessed_pseudogene	6.79E-06	2.95E-04	1.04E+01
BVES	protein_coding	7.49E-05	1.60E-03	1.04E+01
SLC24A4	protein_coding	9.95E-06	3.87E-04	1.04E+01
WEE2-AS1	lncRNA	4.98E-04	5.91E-03	1.05E+01
LINC01012	lncRNA	5.63E-05	1.31E-03	1.06E+01
RFPL1S	lncRNA	8.86E-04	8.63E-03	1.06E+01
TMED2-DT	lncRNA	2.31E-06	1.33E-04	1.06E+01

GINS4	protein_coding	1.18E-04	2.22E-03	1.06E+01
CASC19	lncRNA	5.52E-06	2.56E-04	1.07E+01
TEX41	lncRNA	5.61E-04	6.36E-03	1.07E+01
TRIM73	protein_coding	5.31E-04	6.13E-03	1.07E+01
PRPH2	protein_coding	4.01E-04	5.15E-03	1.07E+01
C6orf132	protein_coding	1.10E-04	2.13E-03	1.07E+01
BMP8A	protein_coding	1.47E-05	5.18E-04	1.08E+01
FRG1-DT	lncRNA	5.45E-04	6.26E-03	1.08E+01
LINC00632	lncRNA	6.63E-04	7.15E-03	1.08E+01
AGXT	protein_coding	1.86E-04	3.04E-03	1.08E+01
FLRT2	protein_coding	3.17E-04	4.33E-03	1.08E+01
LINC02882	lncRNA	7.31E-05	1.58E-03	1.09E+01
MYO15B	protein_coding	1.43E-09	3.69E-07	1.09E+01
BASP1-AS1	lncRNA	4.29E-04	5.39E-03	1.09E+01
TTC22	protein_coding	3.49E-04	4.67E-03	1.09E+01
PKD1L2	protein_coding	1.70E-06	1.05E-04	1.09E+01
LINC00534	lncRNA	7.90E-04	8.02E-03	1.09E+01
LINC02708	lncRNA	1.35E-04	2.42E-03	1.09E+01
MTCO3P12	unprocessed_pseudogene	3.99E-07	3.40E-05	1.10E+01
SLFN14	protein_coding	5.34E-04	6.17E-03	1.10E+01
GARNL3	protein_coding	2.22E-04	3.42E-03	1.10E+01
KRT18P61	processed_pseudogene	4.17E-08	5.64E-06	1.11E+01
EPM2A	protein_coding	1.81E-06	1.10E-04	1.11E+01
ITGA7	protein_coding	9.68E-04	9.19E-03	1.11E+01
CACNB4	protein_coding	9.04E-08	1.00E-05	1.11E+01
LTK	protein_coding	3.42E-10	1.25E-07	1.12E+01
FAR2P1	transcribed_unprocessed_pseudogene	4.24E-04	5.35E-03	1.12E+01
NLGN2	protein_coding	1.71E-05	5.74E-04	1.13E+01
USP32P2	transcribed_unprocessed_pseudogene	5.47E-04	6.28E-03	1.13E+01
ANKRD30B	protein_coding	6.85E-05	1.49E-03	1.13E+01
KRT18P18	transcribed_processed_pseudogene	3.76E-04	4.93E-03	1.13E+01
DNM1	protein_coding	4.76E-04	5.75E-03	1.13E+01
C2orf92	protein_coding	2.15E-04	3.35E-03	1.14E+01
SFTPD-AS1	lncRNA	4.63E-04	5.64E-03	1.14E+01
SEMA5B	protein_coding	1.40E-04	2.48E-03	1.14E+01

BBS5	protein_coding	9.82E-04	9.28E-03	1.14E+01
AGBL4	protein_coding	7.45E-04	7.73E-03	1.14E+01
ZDHHC11	protein_coding	8.70E-04	8.53E-03	1.14E+01
KDM4A-AS1	lncRNA	5.36E-05	1.26E-03	1.15E+01
CRACR2A	protein_coding	6.71E-04	7.21E-03	1.15E+01
CDC20P1	processed_pseudogene	8.92E-04	8.67E-03	1.15E+01
CHRNA10	protein_coding	4.48E-05	1.12E-03	1.15E+01
KIF26A	protein_coding	2.28E-04	3.48E-03	1.16E+01
ADAMTS9-AS2	lncRNA	2.31E-10	8.83E-08	1.16E+01
NMNAT3	protein_coding	6.80E-05	1.48E-03	1.16E+01
COL5A3	protein_coding	1.05E-06	7.23E-05	1.16E+01
WDR11-DT	lncRNA	8.61E-04	8.46E-03	1.17E+01
GRM6	protein_coding	9.55E-04	9.14E-03	1.17E+01
KC6	lncRNA	1.94E-05	6.19E-04	1.17E+01
PCDH9	protein_coding	9.59E-05	1.93E-03	1.17E+01
ZNF696	protein_coding	7.86E-04	7.99E-03	1.17E+01
TF	protein_coding	6.08E-04	6.72E-03	1.17E+01
LINC02028	lncRNA	1.27E-04	2.33E-03	1.18E+01
DNAH17	protein_coding	9.49E-04	9.11E-03	1.18E+01
MKRN9P	transcribed_processed_pseudogene	2.94E-04	4.12E-03	1.18E+01
NPFFR1	protein_coding	3.72E-04	4.89E-03	1.19E+01
SULT1C2	protein_coding	7.11E-04	7.53E-03	1.19E+01
CSPG4P12	transcribed_unprocessed_pseudogene	4.27E-04	5.37E-03	1.19E+01
MARK4	protein_coding	3.28E-05	8.94E-04	1.20E+01
COL4A3	protein_coding	5.22E-04	6.07E-03	1.20E+01
SPATA33	protein_coding	1.27E-05	4.68E-04	1.20E+01
JPH1	protein_coding	2.49E-04	3.70E-03	1.20E+01
TNS1-AS1	lncRNA	6.45E-04	6.99E-03	1.20E+01
MTCO2P12	unprocessed_pseudogene	3.79E-06	1.93E-04	1.21E+01
TMC3-AS1	lncRNA	1.36E-04	2.44E-03	1.21E+01
DGCR5	lncRNA	4.33E-04	5.42E-03	1.22E+01
CPLANE1-AS1	lncRNA	4.63E-04	5.64E-03	1.22E+01
CELSR1	protein_coding	7.40E-04	7.70E-03	1.22E+01

NUDT16-DT	lncRNA	2.25E-04	3.44E-03	1.22E+01
ZNF534	protein_coding	6.43E-04	6.97E-03	1.22E+01
BMP8B	protein_coding	1.94E-04	3.12E-03	1.23E+01
MCF2L	protein_coding	1.54E-05	5.33E-04	1.23E+01
MTCO2P2	processed_pseudogene	5.41E-06	2.53E-04	1.23E+01
AMZ1	protein_coding	6.59E-05	1.45E-03	1.23E+01
TP53TG3D	protein_coding	1.38E-04	2.45E-03	1.24E+01
KCNB1	protein_coding	2.56E-04	3.78E-03	1.24E+01
PRELID2	protein_coding	9.64E-04	9.17E-03	1.24E+01
EBF4	protein_coding	3.84E-04	5.00E-03	1.24E+01
NSRP1P1	processed_pseudogene	8.44E-12	5.37E-09	1.24E+01
SLFN12L	protein_coding	5.79E-04	6.50E-03	1.25E+01
FAM153B	lncRNA	6.17E-04	6.79E-03	1.26E+01
KCNJ6	protein_coding	2.27E-04	3.47E-03	1.27E+01
RNF165	protein_coding	8.61E-04	8.46E-03	1.27E+01
LINC01772	lncRNA	4.36E-05	1.10E-03	1.28E+01
ITPR3	protein_coding	8.30E-06	3.39E-04	1.28E+01
DAPK2	protein_coding	8.07E-04	8.13E-03	1.29E+01
OR52A1	protein_coding	6.38E-04	6.94E-03	1.29E+01
PRKY	transcribed_unitary_pseudogene	2.43E-04	3.65E-03	1.30E+01
CCR12P	transcribed_unprocessed_pseudogene	1.01E-03	9.43E-03	1.31E+01
RBFOX1	protein_coding	4.81E-05	1.17E-03	1.31E+01
CTBP2P8	processed_pseudogene	1.10E-06	7.45E-05	1.31E+01
AR	protein_coding	3.96E-05	1.02E-03	1.32E+01
TTTY10	lncRNA	1.89E-05	6.09E-04	1.32E+01
ZNF727	protein_coding	1.47E-04	2.57E-03	1.32E+01
HABP4	protein_coding	5.49E-06	2.55E-04	1.33E+01
SLC51A	protein_coding	7.98E-04	8.07E-03	1.33E+01
GOLGA6L10	protein_coding	2.91E-04	4.10E-03	1.34E+01
NANOG	protein_coding	9.61E-04	9.16E-03	1.34E+01
SLC4A8	protein_coding	2.26E-07	2.13E-05	1.34E+01
IL6R-AS1	lncRNA	8.67E-04	8.51E-03	1.35E+01
LINC02388	lncRNA	2.34E-04	3.55E-03	1.35E+01
GDNF-AS1	lncRNA	1.10E-04	2.12E-03	1.35E+01

PURPL	lncRNA	5.30E-04	6.13E-03	1.35E+01
RNASEH2B-AS1	lncRNA	3.21E-04	4.37E-03	1.35E+01
OR4D9	protein_coding	7.72E-04	7.90E-03	1.36E+01
C2orf50	protein_coding	6.69E-04	7.20E-03	1.36E+01
KCNMA1	protein_coding	1.70E-04	2.86E-03	1.36E+01
RBM17P4	processed_pseudogene	1.59E-04	2.74E-03	1.37E+01
POTEM	protein_coding	6.71E-05	1.47E-03	1.37E+01
LINC00687	lncRNA	4.24E-04	5.35E-03	1.37E+01
FLJ43315	transcribed_unprocessed_pseudogene	5.66E-07	4.48E-05	1.38E+01
TLCD3B	protein_coding	8.12E-04	8.14E-03	1.38E+01
SEC24B-AS1	lncRNA	4.37E-04	5.45E-03	1.39E+01
LINC01206	lncRNA	4.26E-04	5.37E-03	1.39E+01
SOX2-OT	lncRNA	1.82E-04	3.00E-03	1.39E+01
KIF5C	protein_coding	1.07E-04	2.08E-03	1.40E+01
NBEA	protein_coding	2.58E-05	7.45E-04	1.40E+01
SLC2A11	protein_coding	3.11E-08	4.36E-06	1.40E+01
PPP1R3G	protein_coding	2.81E-04	4.00E-03	1.40E+01
OR8B1P	transcribed_unprocessed_pseudogene	3.26E-05	8.93E-04	1.41E+01
MTND2P28	unprocessed_pseudogene	6.00E-06	2.71E-04	1.41E+01
LINC01550	lncRNA	1.33E-04	2.41E-03	1.42E+01
LINC01962	lncRNA	4.65E-04	5.66E-03	1.42E+01
SHANK3	protein_coding	8.14E-06	3.35E-04	1.42E+01
LACTB2-AS1	lncRNA	2.59E-04	3.81E-03	1.43E+01
OR10H5	protein_coding	3.81E-04	4.97E-03	1.43E+01
SNAP25	protein_coding	9.58E-04	9.14E-03	1.43E+01
DNAH9	protein_coding	1.05E-03	9.77E-03	1.43E+01
SCN1A	protein_coding	3.44E-05	9.22E-04	1.45E+01
LINC02397	lncRNA	1.98E-04	3.16E-03	1.45E+01
NR5A2	protein_coding	4.77E-04	5.76E-03	1.46E+01
GOLGA8R	protein_coding	5.26E-04	6.10E-03	1.46E+01
LINC00607	lncRNA	1.64E-06	1.02E-04	1.47E+01
CACNA1C	protein_coding	1.02E-12	9.68E-10	1.48E+01
CACNA1D	protein_coding	4.09E-04	5.22E-03	1.49E+01

FAT3	protein_coding	6.96E-04	7.41E-03	1.49E+01
HSPA6	protein_coding	2.92E-05	8.23E-04	1.49E+01
CACNG8	protein_coding	9.15E-04	8.86E-03	1.49E+01
OPRD1	protein_coding	6.04E-04	6.70E-03	1.50E+01
C8orf34	protein_coding	8.53E-04	8.43E-03	1.50E+01
TNFRSF9	protein_coding	1.09E-03	9.95E-03	1.51E+01
C2orf16	protein_coding	4.42E-04	5.46E-03	1.51E+01
LINC02241	lncRNA	9.62E-04	9.17E-03	1.52E+01
AQP4-AS1	lncRNA	5.70E-04	6.43E-03	1.52E+01
LINC02614	lncRNA	2.40E-04	3.62E-03	1.52E+01
OPRM1	protein_coding	4.62E-04	5.64E-03	1.53E+01
LINC02551	lncRNA	1.01E-03	9.45E-03	1.53E+01
LINC03000	lncRNA	2.09E-04	3.28E-03	1.53E+01
MIR4500HG	lncRNA	7.69E-04	7.88E-03	1.53E+01
CACNA1A	protein_coding	1.21E-04	2.27E-03	1.53E+01
OR2A25	protein_coding	8.26E-04	8.23E-03	1.54E+01
TSIX	lncRNA	8.76E-04	8.56E-03	1.54E+01
LETR1	lncRNA	1.88E-04	3.07E-03	1.54E+01
CYP46A1	protein_coding	2.23E-05	6.83E-04	1.54E+01
SKA1	protein_coding	1.07E-04	2.08E-03	1.55E+01
USP6	protein_coding	5.37E-10	1.77E-07	1.55E+01
SPN	protein_coding	7.07E-04	7.49E-03	1.56E+01
LINC00342	lncRNA	5.43E-16	1.86E-12	1.57E+01
SLIT3-AS2	lncRNA	4.93E-04	5.89E-03	1.57E+01
OR7D2	protein_coding	7.99E-04	8.07E-03	1.58E+01
LINC00907	lncRNA	2.45E-04	3.67E-03	1.59E+01
LINC02242	lncRNA	2.45E-04	3.67E-03	1.59E+01
KCNN3	protein_coding	8.67E-04	8.51E-03	1.59E+01
TALAM1	lncRNA	1.63E-05	5.55E-04	1.60E+01
TNNI2	protein_coding	5.58E-04	6.34E-03	1.60E+01
LRRIQ1	protein_coding	3.52E-04	4.69E-03	1.61E+01
LINC00504	lncRNA	9.27E-04	8.93E-03	1.64E+01
LINC00824	lncRNA	5.23E-05	1.23E-03	1.65E+01
TCF23	protein_coding	1.60E-04	2.74E-03	1.65E+01
LINC01931	lncRNA	1.88E-05	6.08E-04	1.65E+01
DSG3	protein_coding	3.62E-04	4.79E-03	1.65E+01
OR1D2	protein_coding	1.64E-04	2.79E-03	1.66E+01
KLHL33	protein_coding	7.55E-06	3.19E-04	1.67E+01

NEXN-AS1	lncRNA	1.04E-03	9.67E-03	1.67E+01
OR7E25P	transcribed_unprocessed_pseudogene	4.95E-04	5.90E-03	1.67E+01
CEACAM22P	transcribed_unprocessed_pseudogene	5.82E-04	6.51E-03	1.67E+01
CFAP69	protein_coding	1.08E-06	7.37E-05	1.67E+01
OVCH1-AS1	lncRNA	6.76E-04	7.26E-03	1.69E+01
ZC3H12D	protein_coding	2.42E-05	7.13E-04	1.69E+01
UBQLNL	protein_coding	4.66E-07	3.85E-05	1.70E+01
ADAM28	protein_coding	6.49E-07	4.99E-05	1.70E+01
PRR9	protein_coding	2.21E-04	3.41E-03	1.71E+01
RIMS2	protein_coding	2.28E-05	6.92E-04	1.71E+01
KCNH2	protein_coding	3.46E-04	4.64E-03	1.71E+01
USP2-AS1	lncRNA	6.11E-04	6.75E-03	1.71E+01
ZFP57	protein_coding	5.81E-04	6.51E-03	1.71E+01
HOXC8	protein_coding	1.87E-04	3.05E-03	1.73E+01
ASCL2	protein_coding	1.04E-04	2.05E-03	1.73E+01
VEGFA	protein_coding	8.28E-08	9.40E-06	1.74E+01
LIX1L-AS1	lncRNA	7.05E-04	7.48E-03	1.75E+01
ACSL6	protein_coding	4.33E-04	5.42E-03	1.76E+01
BCL2L14	protein_coding	5.18E-05	1.22E-03	1.77E+01
SLC14A2	protein_coding	2.65E-04	3.86E-03	1.77E+01
SCN5A	protein_coding	6.38E-04	6.94E-03	1.79E+01
ADAM29	protein_coding	2.56E-05	7.44E-04	1.79E+01
DUOX2	protein_coding	7.59E-04	7.82E-03	1.79E+01
PSORS1C1	protein_coding	1.01E-03	9.45E-03	1.80E+01
EIF3EP2	processed_pseudogene	1.32E-04	2.39E-03	1.81E+01
FAM27C	lncRNA	4.65E-05	1.14E-03	1.81E+01
GCAWKR	lncRNA	9.90E-04	9.32E-03	1.81E+01
ASS1P2	processed_pseudogene	4.13E-04	5.25E-03	1.83E+01
CSMD3	protein_coding	2.85E-04	4.04E-03	1.85E+01
EFCAB6	protein_coding	1.56E-04	2.69E-03	1.85E+01
PDE11A	protein_coding	1.22E-04	2.27E-03	1.85E+01
PWRN2	lncRNA	7.23E-04	7.58E-03	1.86E+01
OR10A2	protein_coding	4.27E-04	5.37E-03	1.86E+01
DNAH7	protein_coding	7.84E-05	1.66E-03	1.86E+01
APCDD1L-DT	lncRNA	8.37E-04	8.30E-03	1.87E+01

ADGRF1	protein_coding	8.04E-06	3.34E-04	1.87E+01
HECW1	protein_coding	2.15E-04	3.35E-03	1.87E+01
GABRB2	protein_coding	6.34E-05	1.42E-03	1.88E+01
OR2A1-AS1	lncRNA	3.65E-04	4.82E-03	1.88E+01
PCA3	lncRNA	1.92E-05	6.15E-04	1.89E+01
LINC02362	lncRNA	8.49E-04	8.41E-03	1.89E+01
LINC01727	lncRNA	2.50E-04	3.71E-03	1.91E+01
LINC02054	lncRNA	2.02E-04	3.20E-03	1.91E+01
HRK	protein_coding	3.45E-05	9.22E-04	1.93E+01
CCBE1	protein_coding	1.77E-04	2.94E-03	1.93E+01
LINC00682	lncRNA	8.10E-04	8.14E-03	1.93E+01
GLYATL1	protein_coding	7.94E-05	1.68E-03	1.94E+01
OR7E122P	transcribed_unprocessed_pseudogene	8.56E-04	8.44E-03	1.96E+01
FBXW10B	protein_coding	1.04E-09	2.88E-07	1.96E+01
TENM1	protein_coding	5.03E-04	5.96E-03	1.96E+01
TDRD1	protein_coding	1.04E-06	7.18E-05	1.96E+01
TPH1	protein_coding	7.16E-07	5.44E-05	1.98E+01
ONECUT2	protein_coding	6.63E-04	7.15E-03	1.99E+01
CPB2-AS1	lncRNA	7.20E-06	3.09E-04	2.00E+01
ESRRB	protein_coding	3.48E-04	4.66E-03	2.00E+01
TRDN-AS1	lncRNA	6.09E-05	1.38E-03	2.01E+01
LINC02348	lncRNA	4.43E-04	5.46E-03	2.02E+01
BBS7-DT	lncRNA	6.89E-04	7.34E-03	2.03E+01
TEX26-AS1	lncRNA	6.89E-04	7.34E-03	2.03E+01
ST8SIA1	protein_coding	2.29E-05	6.95E-04	2.04E+01
LHX9	protein_coding	7.43E-04	7.72E-03	2.04E+01
POU5F1B	protein_coding	5.62E-06	2.58E-04	2.05E+01
KIF5A	protein_coding	2.86E-04	4.05E-03	2.05E+01
ZNF774	protein_coding	1.63E-06	1.02E-04	2.05E+01
LINC02984	lncRNA	9.49E-05	1.92E-03	2.07E+01
LINC01506	lncRNA	2.41E-05	7.13E-04	2.09E+01
AIPL1	protein_coding	2.83E-04	4.02E-03	2.10E+01
DLEC1	protein_coding	2.02E-04	3.20E-03	2.10E+01
FAM135B	protein_coding	6.71E-04	7.21E-03	2.12E+01
GCNT3	protein_coding	8.96E-05	1.84E-03	2.12E+01
MTATP6P1	unprocessed_pseudogene	1.13E-09	3.04E-07	2.14E+01
HTR3B	protein_coding	1.28E-04	2.34E-03	2.15E+01

LINC02271	lncRNA	6.15E-04	6.77E-03	2.15E+01
LINC01978	lncRNA	6.90E-04	7.36E-03	2.15E+01
MTND4P12	processed_pseudogene	4.71E-11	2.19E-08	2.15E+01
COL10A1	protein_coding	2.36E-04	3.57E-03	2.19E+01
XKR4	protein_coding	2.65E-06	1.48E-04	2.20E+01
LINC00499	lncRNA	3.02E-05	8.45E-04	2.20E+01
AGAP13P	unprocessed_pseudogene	1.31E-04	2.38E-03	2.20E+01
L3MBTL4	protein_coding	1.53E-05	5.30E-04	2.22E+01
GABRA2	protein_coding	1.31E-04	2.38E-03	2.23E+01
SLC26A4-AS1	lncRNA	5.06E-04	5.97E-03	2.26E+01
LINC02250	lncRNA	6.28E-04	6.87E-03	2.26E+01
MCHR2	protein_coding	5.86E-04	6.55E-03	2.26E+01
OLA1P2	processed_pseudogene	7.63E-04	7.86E-03	2.27E+01
KRT8P32	processed_pseudogene	9.40E-05	1.90E-03	2.28E+01
OR9K1P	transcribed_unitary_pseudogene	5.11E-04	6.01E-03	2.29E+01
KRT18P22	processed_pseudogene	2.64E-04	3.85E-03	2.31E+01
JAKMIP2-AS1	lncRNA	1.57E-04	2.70E-03	2.33E+01
SPAG8	protein_coding	1.55E-08	2.45E-06	2.34E+01
PCDHB9	protein_coding	1.40E-05	5.02E-04	2.34E+01
MUC3A	protein_coding	8.82E-04	8.59E-03	2.34E+01
FAM83F	protein_coding	3.87E-05	1.01E-03	2.37E+01
TAAR5	protein_coding	6.83E-04	7.30E-03	2.38E+01
SCOC-AS1	lncRNA	2.57E-05	7.45E-04	2.38E+01
LINC02346	lncRNA	3.37E-05	9.12E-04	2.39E+01
CACNA1G	protein_coding	4.95E-04	5.90E-03	2.41E+01
PRAL	TEC	1.83E-05	6.00E-04	2.42E+01
LINC01151	lncRNA	5.13E-04	6.02E-03	2.43E+01
RNF157-AS1	lncRNA	2.39E-04	3.60E-03	2.43E+01
LINC01570	lncRNA	1.37E-04	2.44E-03	2.43E+01
ANKRD18A	protein_coding	1.07E-06	7.32E-05	2.44E+01

PCDHGB8P	transcribed_unitary_pseudogene	2.40E-04	3.62E-03	2.46E+01
PGA3	protein_coding	2.15E-04	3.35E-03	2.47E+01
CFHR3	protein_coding	9.44E-04	9.08E-03	2.49E+01
STK32A	protein_coding	1.79E-05	5.89E-04	2.49E+01
ADIPOQ	protein_coding	4.46E-04	5.48E-03	2.50E+01
AADACL3	protein_coding	6.83E-04	7.30E-03	2.51E+01
CD109-AS1	lncRNA	1.09E-06	7.38E-05	2.52E+01
TNR	protein_coding	1.49E-07	1.49E-05	2.54E+01
MTNR1A	protein_coding	8.31E-04	8.25E-03	2.54E+01
GRAMD4P3	processed_pseudogene	7.80E-04	7.96E-03	2.55E+01
STX1B	protein_coding	9.81E-04	9.28E-03	2.56E+01
ARHGEF4	protein_coding	6.77E-06	2.95E-04	2.56E+01
CFAP97D1	protein_coding	2.80E-05	7.95E-04	2.58E+01
PMCHL2	transcribed_unprocessed_pseudogene	4.72E-04	5.72E-03	2.58E+01
NOS1	protein_coding	2.03E-04	3.21E-03	2.58E+01
KLK2	protein_coding	3.59E-04	4.76E-03	2.58E+01
PSMD7-DT	lncRNA	1.09E-04	2.11E-03	2.62E+01
HS6ST3	protein_coding	2.65E-04	3.86E-03	2.63E+01
LINC01441	lncRNA	4.94E-04	5.89E-03	2.63E+01
OR6J1	protein_coding	1.17E-04	2.22E-03	2.64E+01
PPARGC1A	protein_coding	1.41E-04	2.50E-03	2.66E+01
LINC02453	lncRNA	2.29E-04	3.48E-03	2.67E+01
IGSF9B	protein_coding	1.47E-06	9.52E-05	2.67E+01
AJAP1	protein_coding	5.49E-04	6.29E-03	2.67E+01
OR10A3	protein_coding	1.78E-04	2.96E-03	2.68E+01
MTATP8P1	unprocessed_pseudogene	7.12E-04	7.53E-03	2.68E+01
KRT18P35	processed_pseudogene	5.48E-06	2.55E-04	2.69E+01
FBXO15	protein_coding	8.24E-04	8.22E-03	2.70E+01
XRCC6P5	processed_pseudogene	1.17E-04	2.21E-03	2.74E+01
POTEB3	protein_coding	3.66E-05	9.63E-04	2.75E+01
LINC02044	lncRNA	3.73E-04	4.90E-03	2.77E+01
MTND4P35	processed_pseudogene	5.03E-04	5.96E-03	2.77E+01
TMEM178B	protein_coding	1.07E-03	9.86E-03	2.78E+01

LINC01366	lncRNA	5.62E-04	6.36E-03	2.79E+01
ASB18	protein_coding	3.37E-05	9.12E-04	2.79E+01
RET	protein_coding	2.34E-04	3.55E-03	2.81E+01
OR9Q1	protein_coding	3.41E-04	4.59E-03	2.82E+01
LINC00951	lncRNA	3.67E-05	9.64E-04	2.82E+01
ZRANB2-DT	lncRNA	1.46E-06	9.50E-05	2.83E+01
BCL2L2-PABPN1	protein_coding	1.30E-04	2.37E-03	2.83E+01
LRRC37A4P	transcribed_unprocessed_pseudogene	1.22E-05	4.52E-04	2.83E+01
MSRA-DT	lncRNA	9.82E-04	9.28E-03	2.84E+01
TEDC2-AS1	lncRNA	1.06E-03	9.81E-03	2.88E+01
SV2C	protein_coding	1.47E-05	5.18E-04	2.89E+01
LINC02023	lncRNA	2.80E-04	3.99E-03	2.91E+01
LINC01551	lncRNA	2.14E-04	3.35E-03	2.92E+01
LINC00598	lncRNA	6.79E-06	2.95E-04	2.97E+01
DAAM2-AS1	lncRNA	5.66E-08	6.96E-06	3.00E+01
LINC00461	lncRNA	2.50E-04	3.71E-03	3.01E+01
DAW1	protein_coding	2.07E-04	3.25E-03	3.02E+01
OR51A3P	unprocessed_pseudogene	1.21E-04	2.27E-03	3.03E+01
PGM5P2	transcribed_unprocessed_pseudogene	1.13E-05	4.28E-04	3.05E+01
FHOD3	protein_coding	3.25E-04	4.41E-03	3.06E+01
LINC02338	lncRNA	8.13E-04	8.14E-03	3.06E+01
LBX1-AS1	lncRNA	3.13E-04	4.29E-03	3.09E+01
RN7SL37P	misc_RNA	1.17E-04	2.22E-03	3.09E+01
PADI1	protein_coding	7.89E-05	1.67E-03	3.10E+01
ZSWIM5P3	processed_pseudogene	3.16E-04	4.32E-03	3.11E+01
LINC01697	lncRNA	5.31E-07	4.28E-05	3.14E+01
GRIN2A	protein_coding	7.98E-07	5.83E-05	3.15E+01
NPAP1	protein_coding	4.99E-05	1.20E-03	3.15E+01
FAM133CP	processed_pseudogene	3.47E-07	3.05E-05	3.16E+01
CSPG4P11	transcribed_unprocessed_pseudogene	7.50E-04	7.76E-03	3.16E+01
ANKRD30BP2	transcribed_unprocessed_pseudogene	2.99E-04	4.18E-03	3.17E+01

TECTA	protein_coding	2.06E-04	3.24E-03	3.22E+01
PCDHA4	protein_coding	6.74E-05	1.48E-03	3.25E+01
MARCHF11-DT	lncRNA	1.03E-04	2.04E-03	3.28E+01
ATP11B-DT	lncRNA	8.66E-05	1.79E-03	3.30E+01
CDYLP1	unprocessed_pseudogene	4.13E-06	2.05E-04	3.35E+01
AKR7L	protein_coding	3.00E-04	4.18E-03	3.45E+01
GRIN2B	protein_coding	9.74E-07	6.83E-05	3.48E+01
LINC02008	lncRNA	9.68E-05	1.95E-03	3.50E+01
SOX2	protein_coding	5.95E-04	6.62E-03	3.51E+01
HSPA7	unprocessed_pseudogene	8.53E-10	2.50E-07	3.57E+01
LINC01606	lncRNA	4.00E-04	5.15E-03	3.60E+01
E2F3P2	processed_pseudogene	5.74E-04	6.46E-03	3.63E+01
ZNF676	protein_coding	5.78E-04	6.50E-03	3.65E+01
TET1P1	processed_pseudogene	2.38E-04	3.60E-03	3.66E+01
LINC00895	lncRNA	1.46E-05	5.17E-04	3.66E+01
VWC2	protein_coding	5.67E-05	1.32E-03	3.69E+01
ALOX12P2	transcribed_unprocessed_pseudogene	7.38E-05	1.59E-03	3.81E+01
RHPN2P1	unprocessed_pseudogene	1.99E-05	6.31E-04	3.84E+01
OR4C1P	transcribed_unprocessed_pseudogene	1.37E-04	2.44E-03	3.85E+01
TCP10L	protein_coding	2.98E-06	1.61E-04	3.89E+01
LINC01471	lncRNA	1.61E-04	2.76E-03	3.93E+01
LRRC38	protein_coding	1.23E-07	1.27E-05	3.96E+01
LINC02664	lncRNA	4.43E-05	1.11E-03	3.98E+01
MTND4LP30	processed_pseudogene	4.70E-05	1.15E-03	4.01E+01
OR10G3	protein_coding	7.33E-05	1.58E-03	4.02E+01
DNM1P46	transcribed_unprocessed_pseudogene	2.83E-04	4.02E-03	4.06E+01
STEAP3-AS1	lncRNA	2.97E-04	4.16E-03	4.11E+01
COL13A1	protein_coding	3.94E-05	1.02E-03	4.14E+01
CNNM1	protein_coding	1.41E-05	5.04E-04	4.18E+01

NTRK3	protein_coding	2.38E-07	2.21E-05	4.24E+01
OR13I1P	unprocessed_pseudogene	1.74E-04	2.90E-03	4.27E+01
LINC01127	lncRNA	2.00E-04	3.18E-03	4.29E+01
CLDN18	protein_coding	2.76E-04	3.95E-03	4.40E+01
OR4K13	protein_coding	3.92E-06	1.98E-04	5.24E+01
LINC03025	lncRNA	2.66E-04	3.87E-03	5.29E+01
OR51M1	protein_coding	6.01E-06	2.71E-04	5.38E+01
HMGA2-AS1	lncRNA	2.42E-05	7.13E-04	5.51E+01
EMSLR	lncRNA	9.70E-05	1.95E-03	5.76E+01
RNF212	protein_coding	3.81E-07	3.27E-05	5.83E+01
KIAA0895LP1	transcribed_processed_pseudogene	1.31E-05	4.79E-04	5.94E+01
LINC02465	lncRNA	9.49E-06	3.74E-04	7.45E+01
OR5AS1	protein_coding	4.44E-08	5.92E-06	1.08E+02
